# Room temperature stable multitalent: highly reactive and versatile copper guanidine complexes in oxygenation reactions

**DOI:** 10.1007/s00775-021-01849-9

**Published:** 2021-02-17

**Authors:** Melanie Paul, Alexander Hoffmann, Sonja Herres-Pawlis

**Affiliations:** Institute of Inorganic Chemistry, RWTH Aachen University, Landoltweg 1, 52074 Aachen, Germany

**Keywords:** Copper catalysis, Dioxygen activation, Guanidine, Phenazine, Tyrosinase

## Abstract

Inspired by the efficiency of natural enzymes in organic transformation reactions, the development of synthetic catalysts for oxygenation and oxidation reactions under mild conditions still remains challenging. Tyrosinases serve as archetype when it comes to hydroxylation reactions involving molecular oxygen. We herein present new copper(I) guanidine halide complexes, capable of the activation of molecular oxygen at room temperature. The formation of the reactive bis(µ-oxido) dicopper(III) species and the influence of the anion are investigated by UV/Vis spectroscopy, mass spectrometry, and density functional theory. We highlight the catalytic hydroxylation activity towards diverse polycyclic aromatic alcohols under mild reaction conditions. The selective formation of reactive quinones provides a promising tool to design phenazine derivatives for medical applications.

**Supplementary Information:**

The online version contains supplementary material available at 10.1007/s00775-021-01849-9.

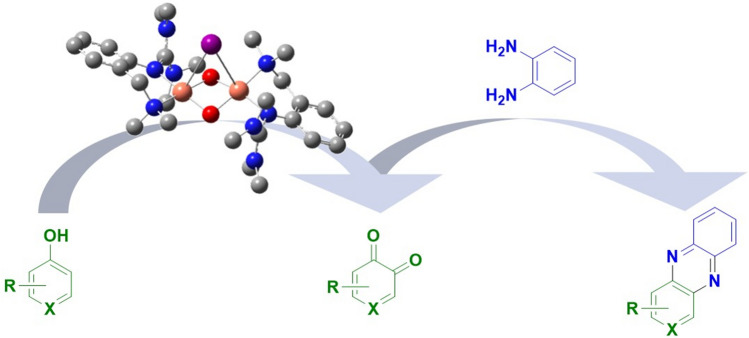

## Introduction

Designing environmentally friendly, selective and efficient oxidation catalysts is one major goal in chemical research [1–3]. Copper-based enzymes demonstrated impressively their ability to activate molecular oxygen, forming catalytically active copper-dioxygen species [4–6]. The type III copper enzyme tyrosinase binds molecular oxygen between two copper centers in a side-on peroxido motif. Tyrosinase catalyzes the *ortho*-hydroxylation of l-tyrosine to l-Dopa as well as the subsequent oxidation to l-Dopaquinone in melanin biosynthesis [7–9].

**Fig. 1:**
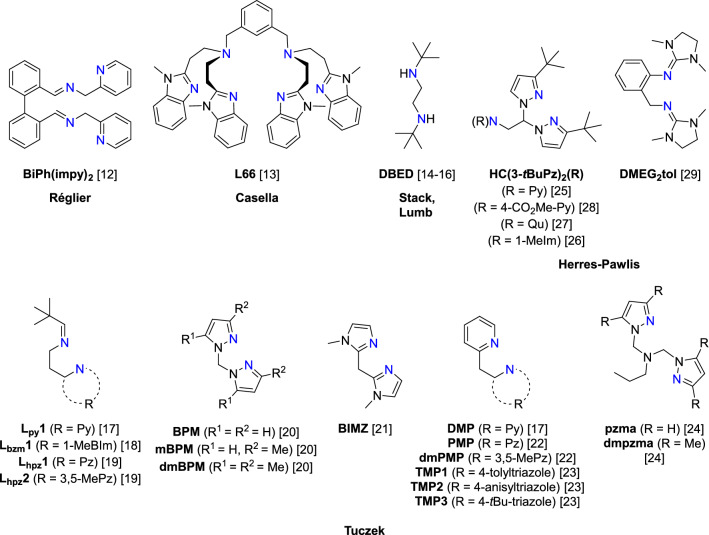
Ligand design in synthetic model systems capable of tyrosinase-like oxygenation reactions [5, 10–29]

Bioinorganic chemistry provided a deep insight into the molecular mechanisms of copper-based enzymes by developing synthetic model systems with tailored oxygenation and oxidation abilities. Over the last years, such small-molecule systems mimicking the active site and functionality of tyrosinase were studied by many research groups [5, 10, 11]. Surprisingly, only a few examples of catalytically active model systems have been reported until now. In 1990, Réglier and co-workers developed the first model system [Cu_2_(MeCN)_4_(BiPh(impy)_2_](PF_6_)_2_, catalyzing the oxygenation reaction of 2,4-di-*tert*-butyl phenol to 3,5-di-*tert*-butyl quinone [12]. Since then, further systems were reported by the working groups of Casella [13], Lumb and Ottenwaelder [14–16], Tuczek [17–24] and Herres-Pawlis [25–29], demonstrating that very different supporting ligand systems feature catalytic transformations of phenolic substrates (Fig. 1).

Among these N-donor ligands, which frequently consist of pyridinyl, pyrazolyl, imine or amine units, guanidines represent strong N-donor ligands due to their high basicity. Guanidines are known to stabilize reactive bis(µ-oxido) dicopper(III) cores and even superoxido species [30–39]. Recently, we have shown that a moderate stability of hybrid guanidine-stabilized bis(µ-oxido) species at low temperatures in the presence of weakly coordinating anions is accompanied by a high tyrosinase-like activity in oxygenation reactions of a large number of phenolic substrates [40]. The reported quinones were found to form exclusively bent phenazines, as predicted by density functional theory (DFT) calculations using the Fukui function. Synthetic access to phenazine derivatives is of major interest, as they feature antibacterial, antitumor, and antimalarial reactivities [41–48].

Herein, we report the synthesis of three novel copper(I) complexes, stabilized by the hybrid guanidine ligand 2-{2-((dimethylamino)methyl)phenyl}-1,1,3,3-tetramethylguanidine (TMGbenza, **L1**), and its activity in activation and transfer reactions of molecular oxygen. We focus on the present anion dependency regarding the stability of the formed bis(µ-oxido) complex, enabling the catalytic oxygenation of polycyclic aromatic alcohols and subsequent condensation of the resulting quinones into phenazines in a one-pot reaction under mild conditions.

## Materials and methods

### General remarks

All synthetic procedures were performed under an inert atmosphere of nitrogen with the use of standard Schlenk or glovebox techniques. All chemicals were purchased commercially (Table S1 in the Supporting Information) and used without further purification unless otherwise noted. Solvents were purified under nitrogen atmosphere via distillation from CaH_2_ or sodium/benzophenone ketyl radical. Some copper salts [49, 50] and hybrid guanidine ligand TMGbenza [40] were synthesized according to literature procedures. Triethylamine was purified by distillation from CaH_2_. Molecular sieves (3 Å, AppliChem) were flame-dried prior to use. Thin-layer chromatography sheets were purchased from MACHEREY–NAGEL (SiO_2_, layer thickness 0.20 mm, fluorescent indicator). Column chromatography was performed on Geduran Si 60 (40–63 µm, Merck).

### Instruments

^1^H and ^13^C{^1^H} NMR spectra were recorded on a Bruker Avance II 400 and Bruker Avance III HD 400 spectrometer at 25 °C in NMR tubes, respectively. Resonances were referenced to the residual solvent signal, relative to TMS. Chemical shifts were assigned with the use of two-dimensional NMR experiments (COSY, HSQC, HMBC). DOSY NMR measurements were performed using ^1^H NMR standard processing applied on pseudo-2D datasets (si = 8 k, lb = 0.3, xf2, abs2). Self-diffusion constants were evaluated by using Dynamics Center. Integration areas were defined manually according to the proton shifts, and its diffusion constants were arithmetically averaged. All NMR data were deposited as original data in Chemotion Repository and are published under an Open Access model. The link to the original data is given in the analytical description.

Elemental analyses were carried out on an elementar vario EL and an elementar vario EL cube instrument.

ESI mass spectra were recorded on a Thermo Fisher Scientific LTQ Orbitrap XL spectrometer at a source voltage of 4.49 kV and a capillary temperature of 299.54 °C.

Cryospray-ionization mass spectrometry (CSI-MS) measurements were performed on an UHR-TOF Bruker Daltonik maXis II, an ESI-quadrupole time-of-flight (qToF) mass spectrometer capable of a resolution of at least 80.000 FWHM, which was coupled to a Bruker Daltonik Cryospray unit. Detection was either in the positive or in the negative ion mode; the source voltage was 3.5 kV. The drying gas (N_2_), to achieve solvent removal, and the spray gas were both held at − 80 °C. The mass spectrometer was calibrated subsequently to every experiment via direct infusion of a l-proline sodium salt solution, which provided a m/z range of singly charged peaks up to 3000 Da in both ion modes.

FT-IR spectra were recorded on a Shimadzu IR Tracer 100 equipped with a CsI beam splitter in combination with an ATR unit (Quest model from Specac utilizing a robust monolithic crystalline diamond) in a resolution of 2 cm^−1^ and on a ThermoFisher Avatar™ 360 spectrometer with the use of KBr pellets or NaCl plates in a resolution of 2 cm^−1^.

UV/Vis spectroscopic measurements were carried out on a Cary 60 spectrophotometer of Agilent Technologies connected via a Cary 50 fiber optic coupler and combined with a fiber-optic quartz glass immersion probe (Hellma, 1 mm) and a tailored Schlenk cell.

The single-crystal diffraction data for **C1a**-**C3a** are presented in Table S2 in the Supporting Information. The data for **C1a**-**C3a** were collected on a Stadivari diffractometer of Stoe with an Eulerian cradle and Dectris Pilatus3 R 200 K hybrid pixel detector with GeniX 3D high flux Mo-Kα radiation (0.71073 Å) at 100 K. The temperature was controlled by using an Oxford Cryostream 800. Crystals were mounted with grease on glass fibers. Data were collected with X-Area Pilatus and integrated with X-Area Integrate and X-Area Recipe. The absorption correction was performed by Gaussian integration with X-Red32. Scaling of reflections was carried out by using X-Area LANA [51–54].

The structures were solved by direct and conventional Fourier methods and all non-hydrogen atoms were refined anisotropically with full-matrix least-squares based on F^2^ (XPREP [55], SHELXT [56], SHELXL [57] and ShelXle [58]). Hydrogen atoms were derived from difference Fourier maps and placed at idealized positions, riding on their parent C atoms, with isotropic displacement parameters U_iso_(H) = 1.2 U_eq_(C) and 1.5 U_eq_(C methyl). All methyl groups were allowed to rotate but not to tip.

Full crystallographic data have been deposited with the Cambridge Crystallographic Data Centre as supplementary no. CCDC–2003620 for **C1a**, CCDC–2003621 for **C2a** and CCDC–2003622 for **C3a**. Copies of the data can be obtained free of charge on application to CCDC, 12 Union Road, Cambridge CB2 1EZ, UK (fax: (+ 44)1223–336-033; e-mail: deposit@ccdc.cam.ac.uk).

### Computational details

Density functional theory (DFT) calculations were performed by using Gaussian 16, Revision B.01 [59]. The geometry optimizations were started based on the geometry of the solid-state structures, using the TPSSh functional [60–62] and the Ahlrichs type basis set def2-TZVP [63–66] as implemented in Gaussian 16, Revision B.01 [59]. For the heavier atom I, effective core potentials (ECP) were used, which were obtained from the TURBOMOLE basis set library [67, 68]. Polarizable Continuum Model (PCM) was utilized as solvent model implemented in Gaussian 16, Revision B.01. The D3 dispersion with Becke–Johnson damping was applied for empirical dispersion correction, which is implemented in Gaussian 16, Revision B.01 [69–72].

## Synthetic procedures

### Synthesis of [Cu(L1)X] (X = I, Br, Cl)

A solution of TMGbenza (**L1**) (24.8 mg, 0.10 mmol, 1.0 eq) in dried acetonitrile (2.0 mL) was added dropwise to a stirring suspension of CuX (0.10 mmol, 1.0 eq) in dried acetonitrile (3.0 mL) during a period of 10 min. The resulting solution was stirred for 15 min and evaporated to dryness (Caution! The complex is very sensitive to oxygen which is indicated by the partial coloration of the precipitate or oil to light green). The residue was washed with dried diethyl ether (3 × 1.0 mL), with dried pentane (3 × 1.0 mL) and dried in vacuo. Single crystals suitable for X-ray diffraction were grown by slow diffusion of diethyl ether into the acetonitrile solution.


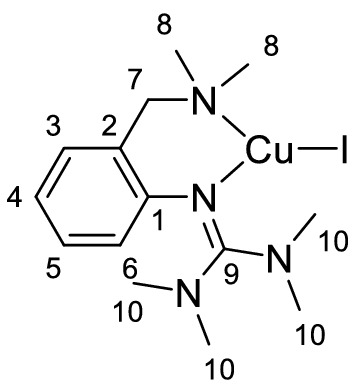
** [Cu(L1)I] (C1a):** The title compound was isolated as a colorless solid (43 mg, 99 µmol, 99%). ^**1**^**H NMR** (400 MHz, Acetonitrile-*d*_3_, 25 °C): *δ* [ppm] = 7.23 (ddd, *J* = 7.8, 7.4, 1.7 Hz, 1H, H6), 7.19–7.16 (m, 1H, H4), 6.92 (td, *J* = 7.4, 1.3 Hz, 1H, H5), 6.47 (dd, *J* = 7.9, 1.3 Hz, 1H, H3), 3.55 (s, 2H, H7), 2.77 (s, 12H, H10), 2.27 (s, 6H, H8). ^**13**^**C{**^**1**^**H} NMR** (101 MHz, Acetonitrile-*d*_3_, 25 °C): *δ* [ppm] = 165.0 (C9), 151.2 (C1), 132.9 (C4), 130.1 (C6), 129.0 (C2), 123.2 (C3), 122.1 (C5), 64.3 (C7), 47.6 (C8), 40.7 (C10). **CHN anal.** calc. for C_14_H_24_CuIN_4_: C, 38.32%; H, 5.51%; N, 12.77%; found: C, 38.25%; H, 5.32%; N, 12.71%. **HRMS-ESI + (MeCN)**: m/z calc. for [(C_14_H_24_N_4_)Cu]^+^: 311.1297, found: 311.1292. **IR (KBr):** *ṽ* [cm^−1^] = 3007 (w, C-H_arom_), 2975 (w, C-H_arom_), 2943 (m, C-H_arom_), 2865 (m, C-H_aliph_), 2829 (m, C-H_aliph_), 2797 (m, C-H_aliph_), 2722 (w), 1594 (m, C = N), 1542 (s), 1522 (vs), 1481 (s), 1463 (s), 1449 (m), 1419 (s), 1406 (s), 1392 (s), 1372 (m), 1330 (m), 1267 (m), 1250 (m), 1230 (m), 1208 (m), 1191 (m), 1175 (m), 1154 (m), 1110 (m), 1062 (m), 1029 (m), 1002 (m), 945 (w), 925 (w), 868 (m), 841 (m), 832 (m), 789 (m), 758 (m), 730 (m), 695 (m), 627 (m). Additional information on the NMR spectra of the target compound **C1a** including original data files is available via Chemotion Repository: https://dx.doi.org/10.14272/DTVVDHUKUBSTJW-UHFFFAOYSA-M.1


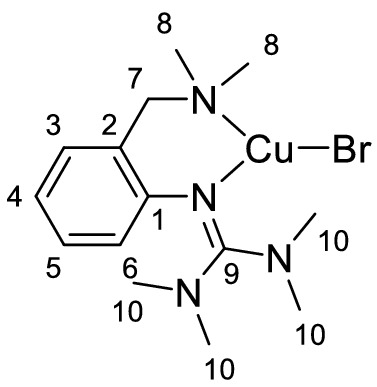
**[Cu(L1)Br] (C2a):** The title compound was isolated as a colorless solid (38 mg, 97 µmol, 97%). ^**1**^**H NMR** (400 MHz, Acetonitrile-*d*_3_, 25 °C): *δ* [ppm] = 7.21–7.17 (m, 1H, H6), 7.13 (td, *J* = 7.7, 1.6 Hz, 1H, H4), 6.86 (td, *J* = 7.4, 1.2 Hz, 1H, H5), 6.44 (dd, *J* = 8.0, 1.1 Hz, 1H, H3), 3.42 (s, 2H, H7), 2.69 (s, 12H, H10), 2.20 (s, 6H, H8). ^**13**^**C{**^**1**^**H} NMR** (101 MHz, Acetonitrile-*d*_3_, 25 °C): *δ* [ppm] = 164.9 (C9), 151.2 (C1), 132.7 (C6), 129.9 (C2), 129.1 (C4), 123.2 (C3), 122.2 (C5), 64.2 (C7), 46.9 (C8), 40.4 (C10). **CHN anal.** calc. for C_14_H_24_CuBrN_4_: C 42.92%; H 6.17%; N 14.30%; found: C 42.81%; H 6.02%; N 14.27%. **HRMS-ESI + (MeCN)**: m/z calc. for [(C_14_H_24_N_4_)Cu]^+^: 311.1297, found: 311.1293. **IR (KBr):** *ṽ* [cm^−1^] = 2961 (w, C-H_arom_), 2924 (m, C-H_arom_), 2852 (w, C-H_aliph_), 1543 (m, C = N), 1518 (s), 1481 (m), 1420 (m), 1406 (m), 1391 (m), 1373 (m), 1331 (w), 1261 (m), 1230 (vw), 1154 (m), 1097 (m), 1065 (m), 1029 (s), 1006 (s), 870 (m), 842 (m), 789 (vs), 756 (s), 730 (m), 694 (m), 630 (vw), 554 (w), 484 (m). Additional information on the NMR spectra of the target compound **C2a** including original data files is available via Chemotion Repository: https://dx.doi.org/10.14272/XYBUOSNKFWKMII-UHFFFAOYSA-M.1


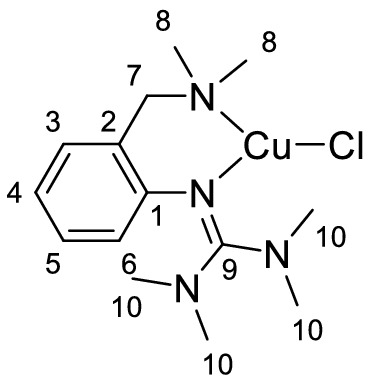
** [Cu(L1)Cl] (C3a):** The title compound was isolated as a colorless solid (33 mg, 95 µmol, 95%). ^**1**^**H NMR** (400 MHz, Acetonitrile-*d*_3_, 25 °C): *δ* [ppm] = 7.22–7.12 (m, 2H, H4 + H6), 6.94–6.85 (m, 1H, H5), 6.44 (d, *J* = 7.8 Hz, 1H, H3), 3.48 (s, 2H, H7), 2.74 (s, 12H, H10), 2.24 (s, 6H, H8). ^**13**^**C{**^**1**^**H} NMR** (101 MHz, Acetonitrile-*d*_3_, 25 °C): *δ* [ppm] = 164.1 (C9), 151.1 (C1), 132.3 (C6), 129.7 (C2), 129.6 (C4), 123.3 (C3), 122.0 (C5), 63.4 (C7), 46.4 (C8), 40.2 (C10). **CHN anal.** calc. for C_14_H_24_CuClN_4_: C 48.41%; H 6.96%; N 16.13%; found: C 48.30%; H 6.89%; N 15.98%. **HRMS-ESI + (MeCN)**: m/z calc. for [(C_14_H_24_N_4_)Cu]^+^: 311.1297, found: 311.1295. **IR (KBr):** *ṽ* [cm^−1^] = 2961 (m, C-H_arom_), 2922 (m, C-H_arom_), 2853 (m, C-H_aliph_), 2811 (w, C-H_aliph_), 1538 (m, C = N), 1530 (m), 1483 (w), 1417 (m), 1401 (m), 1393 (m), 1370 (w), 1335 (vw), 1259 (m), 1232 (vw), 1149 (w), 1098 (m), 1062 (m), 1028 (s), 1005 (s), 869 (m), 843 (m), 791 (vs), 757 (s), 734 (vw), 700 (vw), 553 (vw), 476 (vw), 458 (vw). Additional information on the NMR spectra of the target compound **C3a** including original data files is available via Chemotion Repository: https://dx.doi.org/10.14272/DHTDCYWQWFEGRN-UHFFFAOYSA-M.1

### Synthesis of [Cu_2_(µ-O)_2_(L1)_2_]X_2_ ([O1]X_2_)

Dried and degassed tetrahydrofuran (9.5 mL) was saturated with dioxygen at the respective temperature. The copper(I) complex (0.010 mmol, 2.0 eq) in acetonitrile (0.5 mL) was prepared under inert conditions and added rapidly via a Hamilton syringe. The formation of the bis(µ-oxido) species was followed by UV/Vis spectroscopy.

### Titration of [O1I]^+^ with Copper Salt CuI

Complex **C1a** was oxygenated to give [**O1I**]^+^ (0.5 mM, 1.0 eq) as described above. Excess of O_2_ was removed by three cycles of evacuation and purging with N_2_. A tenfold stock solution of CuI (9.5 mg, 50.0 µmol, 10.0 eq) in acetonitrile (1.0 mL) was prepared and one-tenth of it positioned in a Hamilton syringe. The titrant was added stepwise in 0.1 mL (1.0 eq) steps. The titration experiment was followed by UV/Vis spectroscopy. After stabilization of the optical spectrum, the next aliquot of CuI was injected.

### Salt Metathesis of [O1](PF_6_)_2_ with Bu_4_NX (X = I, Br, Cl)

[**O1**](PF_6_)_2_ in tetrahydrofuran (0.5 mM, 1.0 eq) was synthesized according to our previously reported protocol [40]. Excess of O_2_ was removed by three cycles of evacuation and purging with N_2_. A tenfold stock solution of Bu_4_NX (50.0 µmol, 10.0 eq) in acetonitrile (1.0 mL) was prepared and one-tenth of it positioned in a Hamilton syringe. The titrant was added stepwise in 0.1 mL (1.0 eq) steps. The titration experiment was followed by UV/Vis spectroscopy. After stabilization of the optical spectrum, the next aliquot of Bu_4_NX was injected.

### Competitive oxygenation of C1a and [Cu(L1)(MeCN)]PF_6_

Dried and degassed tetrahydrofuran (9.5 mL) was saturated with molecular oxygen at − 90 °C. The colorless precursor complexes **C1a** (0.005 mmol, 2.0 eq) and [Cu(**L1**)(MeCN)]PF_6_ [40] (0.005 mmol, 2.0 eq) each in acetonitrile (0.25 mL) were prepared under inert conditions and added simultaneously via a Hamilton syringe. The competitive oxygenation of **C1a** and [Cu(**L1**)(MeCN)]PF_6_ was followed by UV/Vis spectroscopy.

### Catalytic oxygenation reactions of phenolic substrates

Flame-dried molecular sieves (400 mg, 3 Å) were placed in a flask. Dried and degassed tetrahydrofuran (18 mL) was saturated with molecular oxygen at room temperature. The colorless precursor complex **C1a** or **C1a**·CuI (0.10 mmol, 2 eq) in acetonitrile (2 mL) was added rapidly via a Hamilton syringe and the bis(µ-oxido) complex [**O1I**](CuI_2_) was formed within two hours. The substrate solution was prepared by dissolving the substrate (1.25 mmol, 25 eq) in dried solvent (tetrahydrofuran or methanol) and subsequent adding of triethylamine (0.35 mL, 2.50 mmol, 50 eq). The solution was injected in one portion into the reaction mixture and stirred at room temperature for at least three hours. 1,2-Phenylenediamine solution (270.3 mg, 2.50 mmol, 50 eq) was prepared by dissolving it in dried tetrahydrofuran (2 mL) and then added to the reaction mixture. After stirring overnight at room temperature, the reaction was quenched by using hydrochloric acid (0.5 M, 50 mL) and EDTA (one spatula). The organic solvents were removed under reduced pressure. The aqueous phase was extracted with methylene chloride (4 × 100 mL). The combined organic layers were dried over Na_2_SO_4_ and evaporated to dryness. The crude product was purified via column chromatography and/or by sublimation. The product was analyzed by NMR spectroscopy.

#### Benzo[a]phenazine (P1) [40, 73]

The title compound was purified by column chromatography (**R**_**f**_ = 0.64; ethyl acetate/n-hexane 15:85) as well as by sublimation and isolated as a yellow solid (121 mg, 0.525 mmol, 42%). ^**1**^**H NMR** (400 MHz, DMSO-*d*_6_, 25 °C): *δ* [ppm] = 9.29–9.23 (m, 1H), 8.38–8.31 (m, 1H), 8.30–8.25 (m, 1H), 8.20 (d, *J* = 9.3 Hz, 1H), 8.11–8.06 (m, 1H), 8.02–7.93 (m, 3H), 7.90–7.82 (m, 2H). ^**13**^**C{**^**1**^**H} NMR** (101 MHz, DMSO-*d*_6_, 25 °C): *δ* [ppm] = 143.1, 142.2, 141.7, 141.1, 133.4, 132.9, 130.5, 130.5, 130.2, 130.1, 129.2, 128.9, 128.5, 128.1, 126.8, 124.6.

#### Quinolino[3,4-b]quinoxaline (P2) [40]

The title compound was purified by column chromatography (**R**_**f**_ = 0.46; ethyl acetate/n-hexane 20:80) and isolated as a yellow solid (162 mg, 0.700 mmol, 56%). ^**1**^**H NMR** (400 MHz, DMSO-*d*_6_; 25 °C): *δ* [ppm] = 9.60 (s, 1H), 9.16 (ddd, *J* = 8.0, 1.6, 0.6 Hz, 1H), 8.44–8.40 (m, 2H), 8.26–8.19 (m, 1H), 8.17–8.06 (m, 2H), 8.01 (ddd, *J* = 8.1, 7.2, 1.6 Hz, 1H), 7.94–7.89 (m, 1H). ^**13**^**C{**^**1**^**H} NMR** (101 MHz, DMSO-*d*_6_, 25 °C): *δ* [ppm] = 155.5, 145.0, 143.8, 142.9, 142.2, 136.6, 133.0, 131.7, 131.5, 129.9, 129.7, 129.3, 128.6, 124.0, 123.9.

#### Pyrido[3,2-a]phenazine (P3) [40]

The title compound was purified by column chromatography (**R**_**f**_ = 0.27; ethyl acetate/n-hexane 20:80) and isolated as a yellow solid (177 mg, 0.765 mmol, 61%). ^**1**^**H NMR** (400 MHz, DMSO-*d*_6_, 25 °C): *δ* [ppm] = 9.51 (dd, *J* = 8.2, 1.7 Hz, 1H), 9.12 (dd, *J* = 4.5, 1.7 Hz, 1H), 8.39–8.29 (m, 2H), 8.23 (s, 2H), 8.05–8.00 (m, 2H), 7.86 (dd, *J* = 8.2, 4.5 Hz, 1H). ^**13**^**C{**^**1**^**H} NMR** (101 MHz, DMSO-*d*_6_, 25 °C): *δ* [ppm] = 152.3, 149.3, 142.5, 142.5, 141.3, 140.9, 134.2, 132.5, 131.2, 131.1, 130.8, 129.3, 129.2, 126.0, 123.1.

#### Pyrrolo[3,2-a]phenazine (P4) [40]

The title compound was purified by column chromatography (**R**_**f**_ = 0.60; ethyl acetate/n-hexane 75:25) and isolated as a yellow solid (112 mg, 0.511 mmol, 41%). ^**1**^**H NMR** (400 MHz, DMSO-*d*_6_, 25 °C): *δ* [ppm] = 12.08 (s, 1H), 8.30–8.22 (m, 2H), 8.06 (dd, *J* = 9.2, 0.9 Hz, 1H), 7.93–7.84 (m, 2H), 7.79 (dd, *J* = 9.2, 0.4 Hz, 1H), 7.58–7.55 (m, 1H), 7.34 (ddd, *J* = 3.0, 2.1, 0.9 Hz, 1H). ^**13**^**C{**^**1**^**H} NMR** (101 MHz, DMSO-*d*_6_, 25 °C): *δ* [ppm] = 141.8, 141.3, 140.7, 140.0, 133.8, 129.8, 129.2, 128.6, 128.6, 124.4, 122.2, 121.4, 121.3, 103.9.

#### Pyrrolo[2,3-a]phenazine (P5) [40]

The title compound was purified by column chromatography (**R**_**f**_ = 0.49; ethyl acetate/n-hexane 50:50) as well as by sublimation and isolated as a yellow solid (159 mg, 0.725 mmol, 58%). ^**1**^**H NMR** (400 MHz, DMSO-*d*_6_, 25 °C): *δ* [ppm] = 12.88 (s, 1H), 8.30–8.23 (m, 2H), 8.13 (d, *J* = 9.1 Hz, 1H), 7.91 (m, 2H), 7.70 (d, *J* = 9.0 Hz, 1H), 7.57 (t, *J* = 2.7 Hz, 1H), 6.80–6.77 (m, 1H). ^**13**^**C{**^**1**^**H}** **NMR** (101 MHz, DMSO-*d*_6_, 25 °C): *δ* [ppm] = 142.2, 140.9, 140.8, 134.8, 130.0, 129.3, 128.7, 128.2, 128.1, 128.0, 126.1, 126.0, 120.5, 104.9.

## Results and discussion

### Synthesis of Cu^I^ complexes

**Scheme 1:**
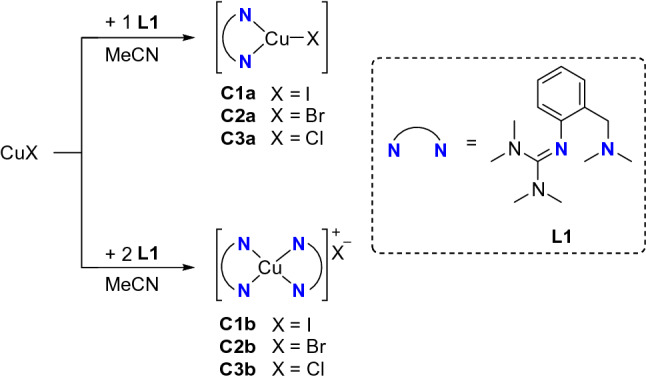
Synthesis of copper(I) hybrid guanidine complexes [Cu(**L1**)X] and [Cu(**L1**)_2_]X (X = I, Br, Cl)

The hybrid guanidine ligand TMGbenza (**L1**) was synthesized according to a procedure reported previously [40]. Depending on the copper salt to ligand ratio, monochelate and bischelate copper(I) complexes were formed (Scheme 1). Reaction of equimolar amounts of copper(I) halides CuX (X = I, Br, Cl) with TMGbenza resulted in the formation of neutral monochelate copper(I) complexes [Cu(**L1**)I] (**C1a**), [Cu(**L1**)Br] (**C2a**), and [Cu(**L1**)Cl] (**C3a**) in high yields. All complexes were analyzed by NMR and IR spectroscopy as well as mass spectrometry. Interestingly, proton shifts in the aromatic and aliphatic region are broadened from **C1a** to **C3a** (Figs. S1, S10, and S15 in the Supporting Information), which was assigned to the increasing ionic character of the Cu-X bond from iodide to chloride.

**Fig. 2:**
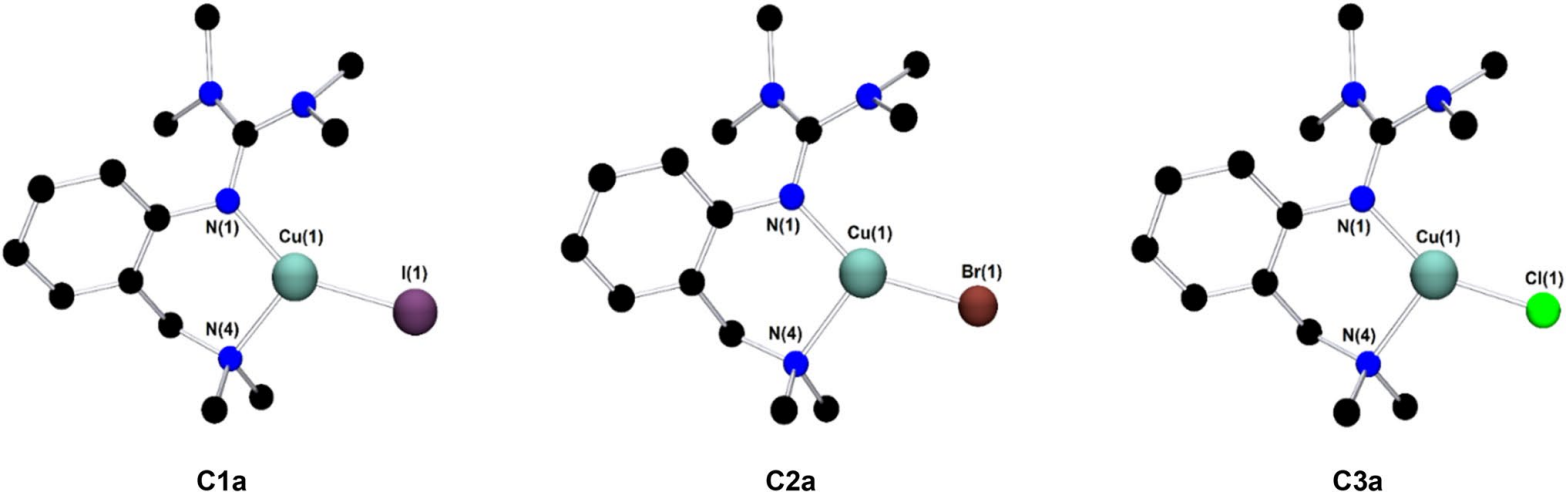
Molecular structures of the complexes **C1a**-**C3a** in the solid state. Hydrogen atoms are omitted for clarity

Single crystals of **C1a**-**C3a** suitable for X-ray diffraction were grown by slow diffusion of diethyl ether into a saturated solution of the complex in acetonitrile (Fig. 2 and Table 1). Complexes **C1a**-**C3a** crystallize orthorhombic in the space group *Pbca* (Table S2 in the Supporting Information). The central copper atom Cu(1) is coordinated in a distorted trigonal-planar fashion by the bidentate hybrid guanidine ligand **L1** and one halide donor. The Cu–N(1) bond lengths are significantly shorter than the Cu–N(4) bond lengths, revealing the higher donor strength of the guanidine moiety compared to the amine donor function. The decreasing Cu-X bond length from complex **C1a** to **C3a** correlates with an increasing Cu(1)-N(4) bond length. Complex **C1a** exhibits an approximately Y-shaped geometry with an N(1)-Cu(1)-X bond angle of 147.63(7)°, which deviates from the ideal 120° angle due to the restricted bite angle of **L1** (97°-99°). The coordination geometry in **C2a** and **C3a** is slightly more distorted Y-shaped, which was also observed in other trigonal-planar complexes [74]. The structure parameter ρ was calculated from the C-N bond lengths within the guanidine moiety, showing a good delocalization of the double bond within the guanidine unit.

**Table 1:** Selected bond lengths [Å] and angles [°] of the Cu^I^ complexes **C1a**-**C3a**

	**C1a**	**C2a**	**C3a**
Cu(1)-N(1)	1.958 (2)	1.943 (3)	1.941 (2)
Cu(1)-N(4)	2.108 (3)	2.130 (3)	2.151 (2)
Cu(1)-X(1)	2.4486 (4)	2.2821 (7)	2.1592 (8)
N(1)-Cu(1)-N(4)	99.34 (10)	98.71 (12)	97.81 (8)
N(1)-Cu(1)-X	147.63 (7)	149.68 (9)	151.66 (6)
ρ^a^	0.98	0.98	0.99

^a^ρ = 2a/(b + c) [75]

Besides monochelate complexes, bischelate copper(I) complexes [Cu(**L1**)_2_]I (**C1b**), [Cu(**L1**)_2_]Br (**C2b**), and [Cu(**L1**)_2_]Cl (**C3b**) can be formed in solution. Temperature-dependent ^1^H DOSY NMR measurements of ligand **L1**, monochelate complex **C1a**, bischelate complex **C1b** and acetonitrile showed a linear dependency of the diffusion constant and the temperature (Fig. S8). The monochelate species **C1a** displays a significantly higher diffusion constant than its related bischelate species **C1b** within the error tolerances (*D*(**C1a**) = 1.81 ± 0.03 10^–9^ m^2^ s^−1^ and *D*(**C1b**) = 1.65 ± 0.04 10^–9^ m^2^ s^−1^ at 299.7 K), revealing a higher dynamic behavior of **C1a** due to its smaller molecular size and concomitantly ruling out the presence of a dimeric species.

### Oxygenation reactions

**Scheme 2:**
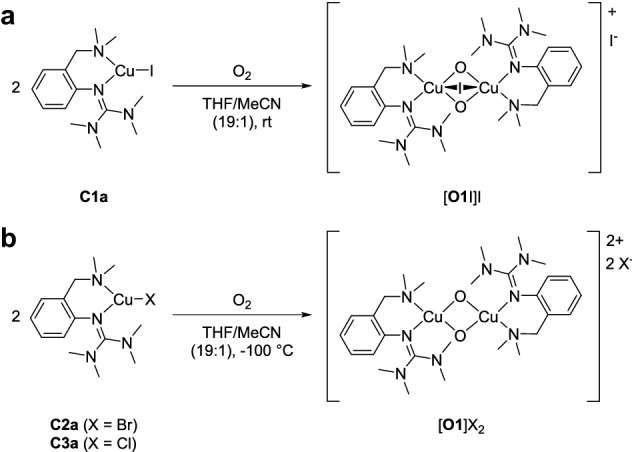
Oxygenation of monochelate complexes **C1a-C3a**

Dioxygen activation was achieved by injecting a solution of copper(I) species **C1a**-**C3a** in acetonitrile in an oxygen-saturated THF solution (Scheme 2).

### UV/Vis spectroscopy

**Fig. 3:**
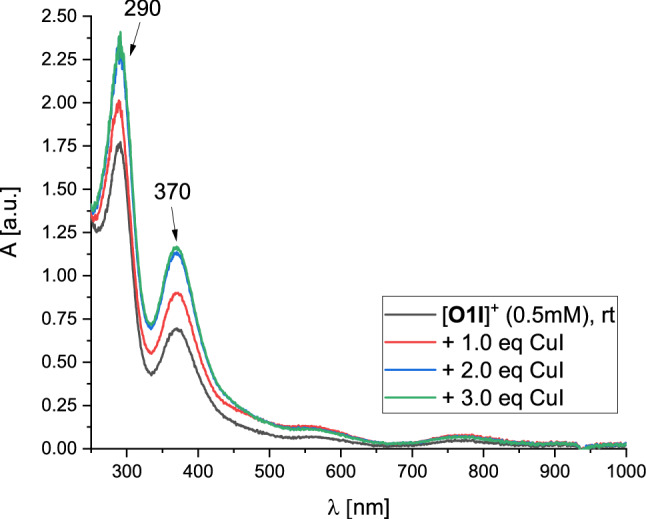
UV/Vis spectra of the oxygenated **C1a** to give [**O1I**]^+^ (black) and the titration with CuI in tetrahydrofuran at room temperature

Oxygenation of monochelate species **C1a** in tetrahydrofuran was accomplished at room temperature, leading to an immediate color change from colorless to reddish-brown generating a bis(µ-oxido) species within two hours (Fig. 3, black line). The oxygenated species of **C1a** was stable at room temperature for at least a week, demonstrating its high stability. By titration experiments and DFT calculations (vide infra), we deduce this bis(µ-oxido) species to contain a [**O1I**]^+^ cation.

The black UV/Vis spectrum in Fig. 3 exhibits two distinct absorption bands at 290 nm and 370 nm after two hours at room temperature, which are in the typical range of bis(µ-oxido) species [5, 6, 10]. But the absorption bands document a blueshift by 20 nm (for the 370 nm absorption) or by 10 nm (for the 290 nm absorption) relative to our recently reported system [**O1**](PF_6_)_2_, which showed spectral features at 280 nm (40,000 M^−1^ cm^−1^) and 392 nm (21,000 M^−1^ cm^−1^) [40]. Same observations were made when **C1a** was oxygenated at − 80 °C (Fig. S20 in the Supporting Information).

Compared to bis(µ-oxido) complex [**O1**](PF_6_)_2_, however, it is conspicuous that the extinction of the absorption bands is significantly lower than expected from previous data, indicating no full formation of the bis(µ-oxido) complex (65%). Since copper iodide species are known for the formation of iodidocuprate clusters [Cu_y_I_x+y_]^x−^ [76–78], several titration experiments were performed to investigate the formed bis(µ-oxido) species and its present anions. Upon titration of [**O1I**]^+^ with hybrid guanidine ligand **L1** the absorption bands of [**O1I**]^+^ remained constant (Fig. S32), showing no effect of additional ligand on the bis(µ-oxido) complex. Similar observations were made by using iodide source tetrabutylammonium iodide as titrant (Fig. S33), revealing no influence of the amount of iodide on [**O1I**]^+^. The titration of [**O1I**]^+^ with one and two equivalents of copper salt [Cu(MeCN)_4_]PF_6_ resulted in a slight decrease of the absorption band at 370 nm (Fig.  S34), resulting from the occurrence of a slight diluting effect while copper salt addition.

When [**O1I**]^+^ is titrated with equivalents of copper iodide (Fig. 3), a slight color change to deeper reddish-brown was observed. The absorption band at 370 nm increased significantly upon addition of one and two equivalents of copper iodide and remained approximately constant upon addition of a third equivalent of copper iodide. As a result, two equivalents of copper iodide are necessary to achieve the complete formation of the bis(µ-oxido) species, indicating the formation of iodidocuprate anions with a copper-to-iodide ratio of 1:2 in the outer sphere of the complex cation [**O1I**]^+^. Therefore, a Cu-I-ratio of the anion of 1:2 is required to achieve a quantitative formation of the bis(µ-oxido) complex, implying small iodidocuprate anions with the stoichiometry of twice [CuI_2_]^−^ or [Cu_2_I_4_]^2−^.

Direct oxygenation of the Cu^I^ adduct **C1a**·CuI in tetrahydrofuran confirmed the previous titration results, leading to similar absorption features at 290 nm (50,000 M^−1^ cm^−1^) and 370 nm (22,000 M^−1^ cm^−1^) within two hours at room temperature (Fig. 3, blue line). ^1^H NMR measurements revealed only marginal discrepancies between **C1a** and **C1a**·CuI (Fig. S7), which results from a very similar chemical environment to enable the same formed bis(µ-oxido) complex [**O1I**](CuI_2_). The extinction coefficient of [**O1I**](CuI_2_) is slightly higher than the one of [**O1**](PF_6_)_2_. The absorption bands were also stable for at least seven days. The CT bands at 450 nm (4300 M^−1^ cm^−1^) and 570 nm (1700 M^−1^ cm^−1^) were found to be more intense compared to the oxygenation of **C1a** as expected. This agrees with the more intense red color of the reaction solution, resulting from the interaction of the guanidine π-system and the iodidocuprate anion (see DFT section, vide infra). The high stability of [**O1I**](CuI_2_) is remarkable because the stability of most bis(µ-oxido) species is limited to very low temperatures [5, 6]. Furthermore, complex [**O1I**](CuI_2_) represents to the best of our knowledge the first example of an iodidocuprate-stabilized bis(µ-oxido) species.

In analogy to complex **C1a**, oxygenation of the monochelate complexes **C2a** and **C3a** resulted in the formation of a bis(µ-oxido) species, exhibiting the characteristic absorption features (Table 2). Nevertheless, the oxygenation process required low temperatures at − 100 °C due to the high reactivity of the greenish bis(µ-oxido) intermediate, resembling the characteristics of [**O1**](PF_6_)_2_ [40]. Both [**O1**]Br_2_ and [**O1**]Cl_2_ were formed within seconds at − 100 °C and decayed very quickly afterwards (Scheme 2b and Sects. 3.2–3.3 in the Supporting Information). The UV/Vis spectra of [**O1**]Br_2_ and [**O1**]Cl_2_ are very similar to [**O1**](PF_6_)_2_ (see Table 2) and show no hint for a halide-bridged species as [**O1I**]^+^. This is in accordance with the smaller anion size of chloride and bromide.

**Table 2:** Experimental UV/Vis transitions of the bis(µ-oxido) species [**O1**](PF_6_)_2_, [**O1I**](CuI_2_), [**O1**]Br_2_ and [**O1**]Cl_2_

	*λ* [nm] (ε [M^−1^ cm^−1^])	
[**O1**](PF_6_)_2_ [40]	392 (21,000)	280 (40,000)
[**O1I**](CuI_2_)	370 (22,000)	290 (50,000)
[**O1**]Br_2_	399 (21,000)	270 (50,000)
[**O1**]Cl_2_	386 (21,000)	270 (50,000)

Surprisingly, similar to the oxygenation of the monochelate species **C1a**-**C3a**, a solution of bischelate complex **C1b**-**C3b** can be oxygenated at low temperatures as well. The resulting bis(µ-oxido) species [**O1**]^2+^ depicted similar absorption features compared to the oxygenated complexes **C1a**-**C3a**, except the extinction was significantly lower. With a maximum of approximately 50% quantity of the formed bis(µ-oxido) species, the oxygenation process indicates an existing dynamic equilibrium between monochelate and bischelate species in solution, in which only the monochelate complex reacts with dioxygen. The bischelate species is resistant to dioxygen due to the saturated coordination sites of its copper center by two equivalents of ligand **L1**.

### Mass spectrometry

**Fig. 4:**
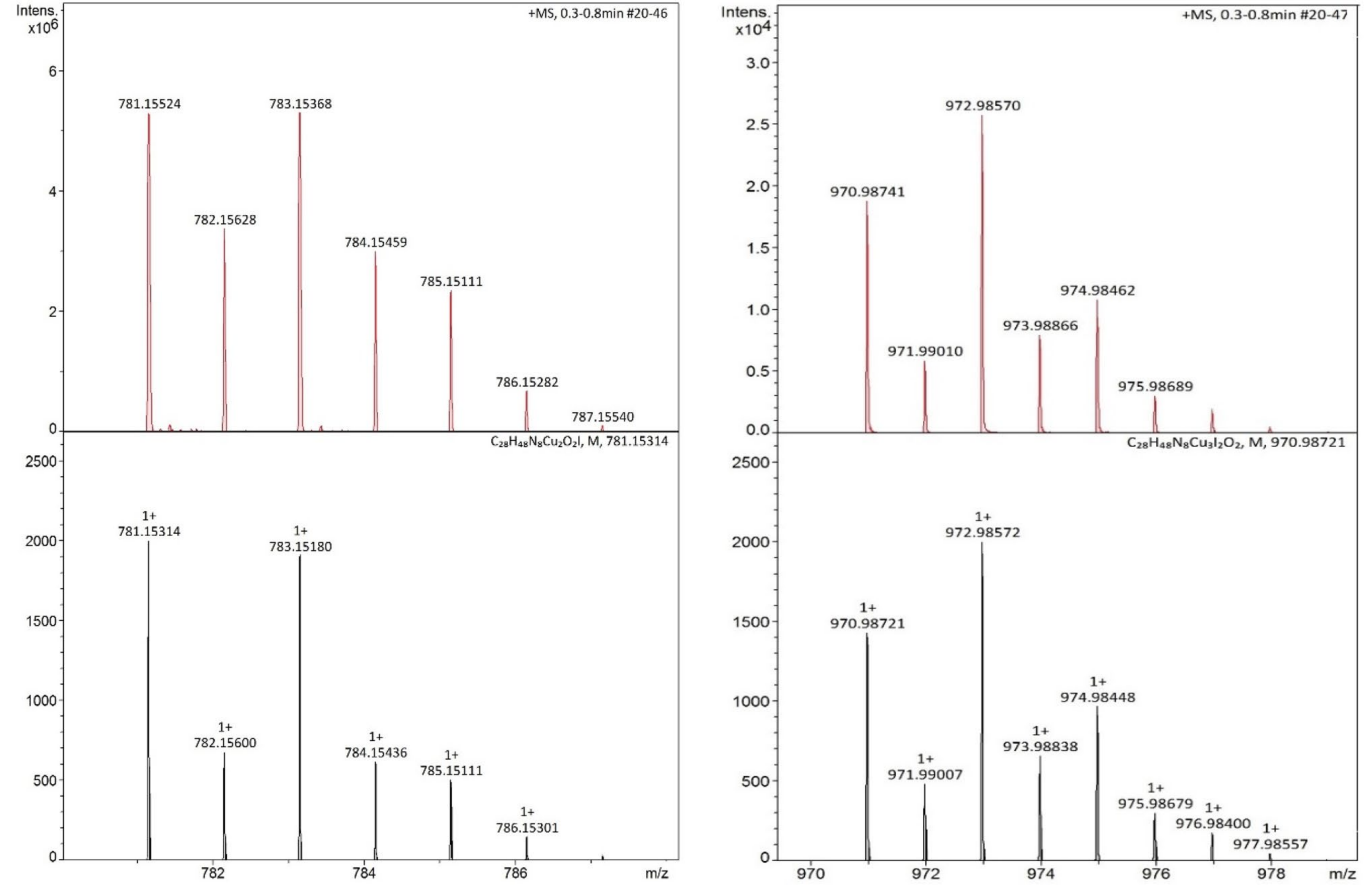
Cryo-UHR-ESI mass spectrometry of [**O1I**]^+^ (left) and [**O1**](CuI_2_)^+^ (right) in tetrahydrofuran at − 80 °C, which were observed in the oxygenation of **C1a** and **C1a**·CuI (red: experimental, black: calculated)

This dynamic equilibrium was also observed by using cryo-UHR-ESI mass spectrometry: measurements of the oxygenation reactions of **C1a**-**C3a** were performed at − 100 °C immediately after the injection of copper(I) complex solution and again after approximately three minutes. Oxygenating **C1a**-**C3a**, the isotopic pattern and corresponding m/z value of [Cu(**L1**)_2_]^+^ were found immediately after the injection in the positive mode (Fig. S23). Accordingly, the isotopic pattern and corresponding m/z values of [CuX_2_]^−^ were found in the negative mode in all cases. The intensity of these signals decreased after a few minutes in favor of the formed corresponding bis(µ-oxido) species [**O1**](X)^+^ (X = I, Br, Cl). Measurements of the oxygenation reaction of both **C1a** and **C1a**·CuI in tetrahydrofuran exhibited the isotopic pattern of the monocationic species [**O1I**]^+^ and [**O1**](CuI_2_)^+^ in the positive mode (Fig. 4), confirming the occurrence of iodidocuprate anions stabilizing the bis(µ-oxido) core. By using the negative mode, the isotopic pattern and corresponding m/z values of iodide and [CuI_2_]^−^ were detected in both oxygenation reactions (Figs. S24 and S25). The isotopic pattern and corresponding m/z values of the oxygenation of **C2a** and **C3a** were also observed, exhibiting the mass spectrum of the monocationic species [**O1**](Br)^+^ and [**O1**](Cl)^+^ in the positive mode (Figs. S26 and S28). Interestingly, only bromido- and chloridocuprate anions were detected in the negative mode during both measurement times when oxygenating **C2a** and **C3a** (Figs. S27 and S29). Bromide and chloride were observed in neither case, which is in accordance with the occurring equilibrium and the very fast formation of the bis(µ-oxido) species [**O1**]Br_2_ and [**O1**]Cl_2_. It is also worth to mention that only the halidocuprate anions with the composition of [CuX_2_]^−^ (X = I, Br, Cl) were detected during the reaction (Figs. S24, S27, S29), presumably due to the high reactivity of halidocuprates in solution compared to often observed larger clusters in the solid-state [76–78].

### DFT study on [O1I]^+^ and [O1]^2+^

**Fig. 5:**
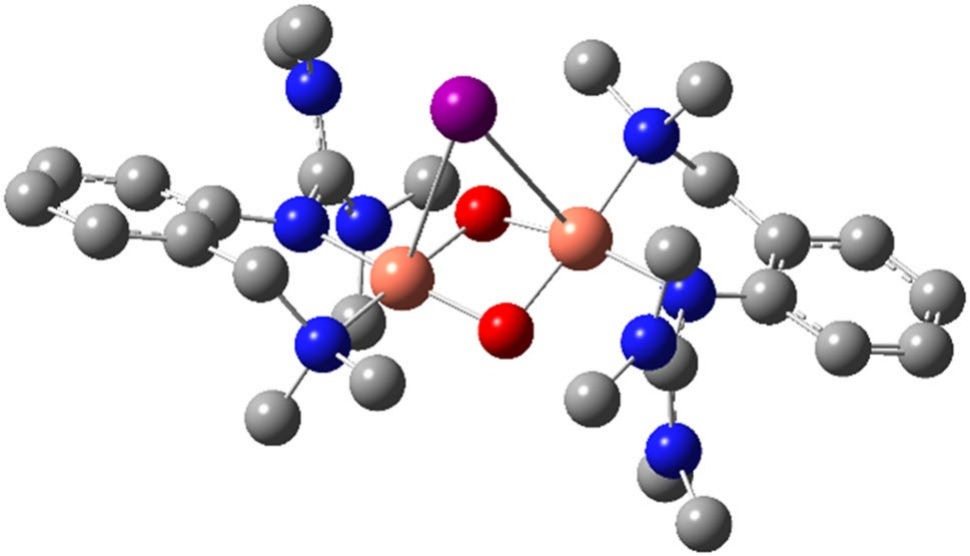
Calculated structure of [**O1I**]^+^ (TPSSh/def2-TZVP, THF-PCM, GD3BJ)

DFT calculations were performed to investigate the influence of the iodide in the bis(µ-oxido) species [**O1I**]^+^. Simulations confirm the presence of an iodide-bridged bis(µ-oxido) species (Fig. 5). Selected bond lengths and the Cu···Cu vector are summarized in Table 3. This structure motif was also observed for the bis(µ-alkoxido) dicopper(II) complex of another guanidine system [39]. The iodide bridge enforces a slight butterfly distortion of the Cu_2_O_2_ moiety. The selected bond lengths are slightly smaller than for a typical bis(µ-oxido) species [5, 6].

**Table 3:** Selected bond lengths [Å] and Cu···Cu distance [Å] of [**O1I**]^+^ (TPSSh/def2-TZVP, THF-PCM, GD3BJ)

	**[O1I]**^**+**^
Cu–N(gua)	1.930/1.925
Cu–N(amine)	1.963/1.9661
Cu–O	1.810/1.805//1.805/1.803
Cu–I	3.395/3.511
Cu···Cu	2.689

**Fig. 6:**
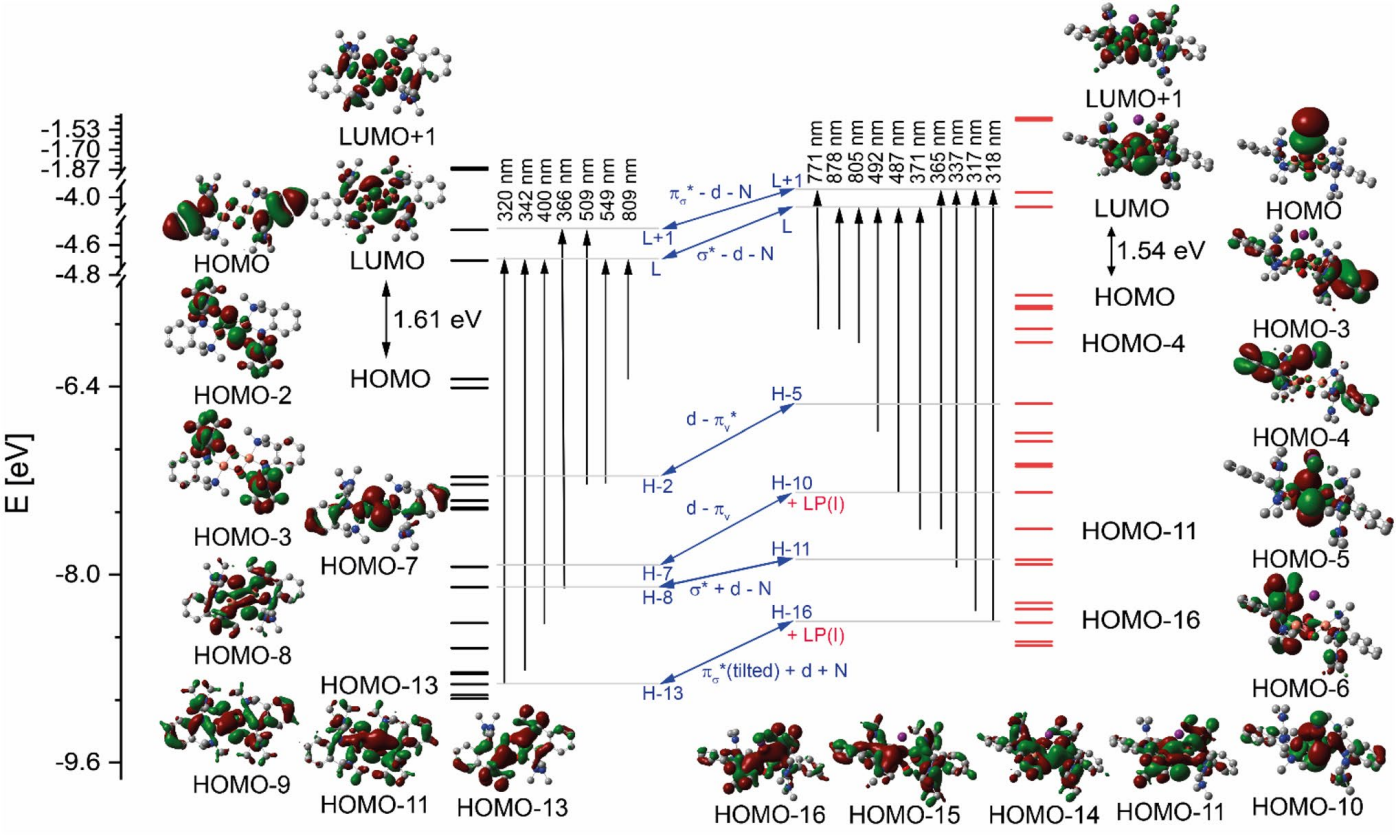
Molecular orbital and energy correlation of the [**O1**]^2+^ species (left) and of the [**O1I**]^+^ species (right). Main excitations are summarized (calculated by TD-DFT). The corresponding interactions of the MO and the influence of the iodide of the UV/vis spectra (middle) are presented

TD-DFT calculations were performed to analyze the observed blueshift of the two characteristic bands in the UV/Vis spectra of [**O1**]^2+^ and [**O1I**]^+^. In the bis(µ-oxido) species [**O1**](PF_6_)_2_, the experimental absorption band at 392 nm (TD-DFT: 366 nm) results from a transition of the bonding interaction of the Cu d orbitals with the σ* orbital (HOMO-8 in Fig. 6, left) to the antibonding interaction of Cu d orbitals with the π_σ_* orbital (LUMO + 1 in Fig. 6, left). The experimentally found transition at 280 nm (TD-DFT: 320 nm) represents the transition from the bonding interaction of the Cu d orbitals and the π_σ_* orbital (HOMO-13 in Fig. 6, left) to the antibonding interaction of the Cu d orbitals with the σ* orbital (LUMO in Fig. 6, left). These classical transitions of bis(µ-oxido) species were also found for other guanidine-stabilized bis(µ-oxido) species [31, 79]. Moreover, two additional transitions to the LUMO were calculated: one from the HOMO-9 (bonding interaction of the Cu d orbitals and the σ of the bis(µ-oxido) unit; TD-DFT: 400 nm) and the second from the HOMO-11 (antibonding interaction of the Cu d orbitals and the σ* orbital of the bis(µ-oxido) moiety, TD-DFT: 342 nm). The weaker transitions at higher wavelengths are transitions from the lone pair of the N(amine) to the LUMO or LUMO + 1 or at even higher wavelengths of the π* orbitals of the ligand to the LUMO.

The iodide in [**O1I**]^+^ possesses three lone pairs (HOMO – HOMO-2), which cause a shift of three orbitals in comparison to the [**O1**]^2+^ species. Therefore, the antibonding interaction of the Cu d orbitals and the π_v_* orbital of the bis(µ-oxido) moiety represent the HOMO-2 in [**O1**]^2+^ and the HOMO-5 in [**O1I**]^+^ (Fig. 6, Table S4 and S5). The iodide LP is involved in other molecular orbitals, resulting in a more complex MO scheme of the iodide-bridged bis(µ-oxido) core in comparison to the MO of the “simpler” bis(µ-oxido) core [**O1**]^2+^. In [**O1I**]^+^, only one classical transition was observed: the bonding interaction of the Cu d orbitals with the σ* orbital of the bis(µ-oxido) moiety (HOMO-11 in Fig. 6, right) to antibonding interaction of Cu d orbitals with the π_σ_* orbital (LUMO + 1 in Fig. 6, right). The calculated transitions at 318 nm and 337 nm contain iodide contributions. The transition at 318 nm is a bonding interaction of the π_σ_* orbital and Cu d orbitals and the LP of the iodide (HOMO-16, Fig. 6, right) with the LUMO + 1. The intense transition at 337 nm is the transition from the HOMO-14 (antibonding interaction of the Cu d orbitals and the σ of the bis(µ-oxido) core, Fig. 6, right) and additionally the HOMO-16 to the LUMO. There are two other transitions in the classical region: the transition at 317 nm, the transition of the HOMO-15 (bonding interaction of the linear combination of π_v_ with π_σ_ with Cu d orbitals, Fig. 6, right) to the LUMO + 1 and the transition at 371 nm, the HOMO-11 to the LUMO. The tailing of the 379 nm band in the experimental UV/Vis spectrum is caused by two transitions: a transition from the HOMO-10 (antibonding interaction of the Cu d orbitals with the π_v_ and bonding interaction with the LP of the iodide, Fig. 6, right) to the LUMO and a transition of the π* orbitals of the guanidine moiety (HOMO-6) to the LUMO + 1. The transitions at higher wavelengths are all interactions of the ligand **L1**. The TD-DFT calculations predict two interactions of the π* orbitals of the whole ligand system (HOMO-3, Fig. 6, right) to the LUMO + 1 (771 nm) and the LUMO (878 nm) and one of the π* orbitals of **L1** and the LP of the iodide (HOMO-4, Fig. 6, right) to the LUMO (805 nm). These calculated transitions from the TD-DFT study explain the different UV/Vis spectra of [**O1**]^2+^ and [**O1I**]^+^.

### Salt metathesis

**Fig. 7:**
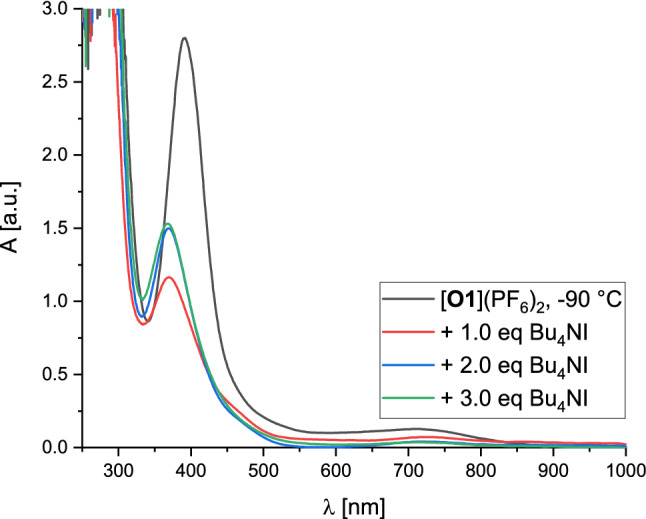
UV/Vis spectra of the salt metathesis of [**O1**](PF_6_)_2_ (1.25 mM) with Bu_4_NI in tetrahydrofuran at − 90 °C

Since the bis(µ-oxido) species [**O1I**](CuI_2_) showed an unusual high stability at room temperature, especially compared to its very similar relative [**O1**](PF_6_)_2_, titration experiments were performed to interconvert both species (Fig. 7). Starting from the khaki-colored bis(µ-oxido) complex [**O1**](PF_6_)_2_ [40] in tetrahydrofuran at − 90 °C, aliquots of the iodide source Bu_4_NI were added stepwise.

Upon addition of one equivalent of Bu_4_NI the reaction solution changed its color from khaki to greenish-brown. The absorption band at 390 nm shifted to 370 nm within minutes under an immense loss in intensity and remained constant after 40 min. The iodide-bridged bis(µ-oxido) core and the iodidocuprate anion are formed according to a different stoichiometry causing the loss in intensity in the UV/Vis spectrum (indicating the decay of the original [**O1**](PF_6_)_2_ species). The formation of iodidocuprates provide an additional supporting ligand for the reactive bis(µ-oxido) core. Adding a second equivalent of Bu_4_NI to the reaction solution led to a color change to reddish-brown, as observed for the direct oxygenation of **C1a**. The bis(µ-oxido) band at 370 nm increased significantly, which stabilized after one hour. No further changes in color and spectral features were observed by adding a third equivalent of Bu_4_NI, revealing a completed salt metathesis after the addition of two equivalents of the iodide source. Upon warming up the reaction solution to room temperature the absorption band at 370 nm remained constant, demonstrating that an easy exchange of the present anion increases the stability of the bis(µ-oxido) species by over 100 °C. These salt metatheses were also performed by using halide sources Bu_4_NX (X = Br, Cl) leading to the bis(µ-oxido) species [**O1**]Br_2_ and [**O1**]Cl_2_ (Figs. S33 and S34).

### Competitive oxygenation of C1a and [Cu(L1)(MeCN)]PF_6_

**Fig. 8:**
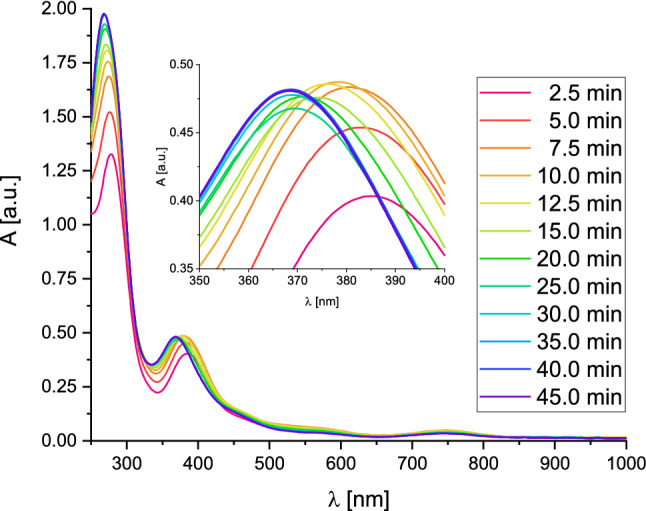
UV/Vis spectra of the formation of [**O1I**](CuI_2_) (0.25 mM) in tetrahydrofuran at − 90 °C (Inset: expansion of the formation of [**O1I**](CuI_2_))

Although the formation of [**O1**](PF_6_)_2_ takes just a few minutes at − 90 °C [40], the oxygenation process of **C1a** is only completed after two hours at room temperature. Therefore, copper(I) species **C1a** and [Cu(**L1**)(MeCN)]PF_6_ were oxygenated simultaneously at − 90 °C to investigate the competitive formation of the resulting bis(µ-oxido) species (Fig. 8).

Initial formation of [**O1**](PF_6_)_2_ was observed within the first three minutes (absorption band at 390 nm), as expected [40]. A wavelength shift to 380 nm after 10 min, which is stable for a few minutes, indicated the occurrence of both species [**O1**](PF_6_)_2_ and [**O1I**](CuI_2_). After 30 min the absorption band was shifted further to 370 nm and remained constant, henceforth resulting from the formation of the more stable bis(µ-oxido) dicopper(III) iodidocuprate complex [**O1I**](CuI_2_). The absorbance in the UV/Vis spectrum remained constant upon transition to [**O1I**](CuI_2_) due to the stabilizing effects of the iodidocuprates on the bis(µ-oxido) core. The coordination of these anions to a copper center caused the wavelength shift of the complex cation. Furthermore, it has to be highlighted that the complexes [**O1**](PF_6_)_2_ and [**O1I**](CuI_2_) are formed in totally different time scales: [**O1**](PF_6_)_2_ within minutes and [**O1I**](CuI_2_) within hours.

### Catalytic oxygenation reactions of phenolic substrates

The bis(µ-oxido) complex [**O1**](PF_6_)_2_ has already proven its remarkable ability to activate C-H bonds in hydroxylation reactions towards many different substrate classes [40]. Only few tyrosinase model systems are known to promote catalytic substrate conversion and even fewer catalysts exhibited room temperature stability and catalytic activity [10].

Since a simple switch of the present anion achieved a great leap in stability, bis(µ-oxido) species [**O1I**](CuI_2_) was tested in catalytic oxygenation reactions of phenolic substrates at room temperature. In this reaction, polycyclic aromatic alcohols were oxygenated in *ortho*-position and the resulting quinones were captured by 1,2-phenylenediamine in a condensation reaction to give stable phenazines (Table 4). This strategy was already used earlier to capture the reactive quinones [40, 80]. From a library of possible substrates, polycyclic aromatic alcohols with two quinone product possibilities were chosen to investigate the influence of the iodide-bridged bis(µ-oxido) species on the selectivity of the hydroxylation reaction. Upon two substrates leading to the same phenazine product, only one of them were tested. The reaction procedure was adapted by a protocol which originated from Bulkowski and was modified by Tuczek and co-workers [17, 81]. A catalyst concentration of 4 mol% referred to the full formation of [**O1I**](CuI_2_) was chosen according to our study on [**O1**](PF_6_)_2_.[40] Triethylamine was used as auxiliary base in two equivalents referred to the substrate. Flame-dried molecular sieve (3 Å) serve as an intercepting agent for produced water molecules during the reaction to avoid catalyst decomposition since bis(µ-oxido) complex [**O1I**](CuI_2_) is only stable towards a small amount of water (Fig. S35 in the Supporting Information).

**Table 4:**
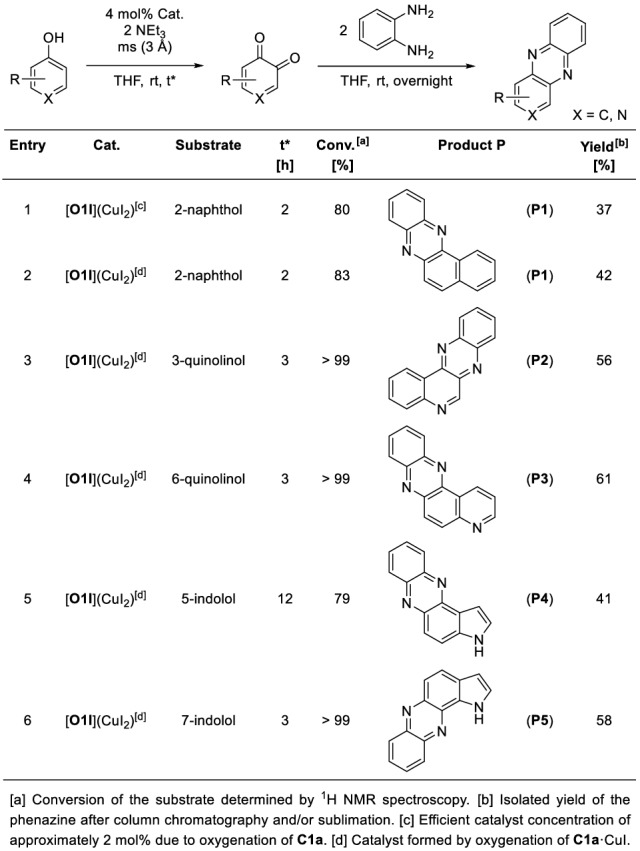
Catalytic oxygenation reactions of polycyclic aromatic alcohols mediated by bis(µ-oxido) species [**O1I**](CuI_2_)

The conversion of 2-naphthol was investigated by comparing the oxygenation process in the presence of both precursor species **C1a** (Table 2, entry 1) and **C1a**·CuI (entry 2), as [**O1I**](CuI_2_) was not formed quantitatively when **C1a** was oxygenated. Although 2-naphthol has two possible hydroxylation positions, the location of the electrophilic attack was predicted by a large value of the negative Fukui function, leading to bent phenazines [40]. 2-Naphthol was transformed into its quinone form within two hours in > 80% conversion and subsequently converted into benzo[a]phenazine (**P1**), which was purified by column chromatography to afford 37–42% isolated yield (entries 1–2). Catalysts [**O1I**](CuI_2_) showed higher activity in the hydroxylation reaction when oxygenated **C1a**·CuI was used. Therefore, this bis(µ-oxido) species was evaluated hereinafter towards other substrate classes. When copper iodide was oxygenated without a supporting ligand system, only a very small amount of 2-naphthol was converted, only leading to traces of **P1** as expected (Sect. 5.3 in the Supporting Information).

3-quinolinol was fully converted by [**O1I**](CuI_2_) within three hours at room temperature to give quinolino[3,4-b]quinoxaline (**P2**) after condensation with 1,2-phenylenediamine (entry 3). **P2** was purified by column chromatography as well as by sublimation and isolated in 56% yield. Similarly, 6-quinolinol was transformed quantitatively to afford pyrido[3,2-a]phenazine (**P3**) in 61% isolated yield (entry 4). C-H functionalization of the pyrrole ring of indolols were achieved by [**O1I**](CuI_2_) leading to pyrrolophenazines **P4** and **P5** in 41–58% yield (entries 5–6). An increase of the hydroxylation reaction time to 12 h did not lead to undesired side reactions, underlining the high selectivity in the oxygenation reaction mediated by bis(µ-oxido) complex [**O1I**](CuI_2_). In comparison to our previously reported system [**O1**](PF_6_)_2_, bis(µ-oxido) species [**O1I**](CuI_2_) revealed an overall higher activity in hydroxylation reactions. The additional steric demand of the iodide bridge between the copper centers depicted no influence on the Cu_2_O_2_ core accessibility towards exogenous substrates. Instead, the achieved room temperature stability of [**O1I**](CuI_2_) enhances the activity of the catalyst. As a result, the accessibility of the reactive center from one direction appears to be sufficient to successfully perform catalytic oxygenation reactions.

The observed high reactivity of the bis(µ-oxido) species poses the question, how this formal “oxido” species can exert an electrophilic hydroxylation reactivity. The concept of the inverted ligand field [82] helps to explain this apparent contradiction: the bis(µ-oxido) species possesses electron holes on the formal bis(µ-oxido) moieties since LUMO and LUMO + 1 have large oxygen p character (see Fig. 6 and Ref. [82]). Besides, another question points to the bridging iodide which “survives” the close proximity to two Cu(III) centers, or at least an oxidative species. We relate this stability to the diminished outer-sphere oxidative ability of [**O1**]^2+^ and [**O1I**]^+^ since we did never observe C–C coupling products. Under these circumstances, we propose that I_2_ cannot be formed.

## Conclusion

In conclusion, we presented the synthesis and characterization of a bis(µ-oxido) dicopper(III) iodidocuprate species, which was stabilized by the hybrid guanidine ligand **L1**. The bis(µ-oxido) complex exhibited surprisingly high stability at room temperature. Titration experiments of the bis(µ-oxido) species indicated the formation of iodidocuprate anions which were also found in cryo-UHR-ESI measurements.

The bis(µ-oxido) system showed its great flexibility with respect to its stability and spectroscopic features, providing a toolbox for tailored dioxygen transfer reactions. A simple salt metathesis from weakly coordinating anions to coordinating anions caused a wavelength shift of the bis(µ-oxido) complex and allowed a great leap in stability from − 90 °C to room temperature, underlining the influence of an additional halide ligand on the bis(µ-oxido) dicopper(III) core. Even in a competitive oxygenation reaction, the more stable bis(µ-oxido) species was formed. In addition, compound [**O1I**](CuI_2_) revealed a higher activity in C-H functionalization reactions towards different classes of polycyclic aromatic alcohols than its relative [**O1**](PF_6_)_2_. Resulting quinones were directly transformed into their stable phenazine form and were isolated in good yield. Achieved mild reaction conditions in a catalytic one-pot reaction pave the way for future transformation reactions applied on a larger scale on the route to industrial applications.

## Supplementary Information

Below is the link to the electronic supplementary material.

Supplementary file1 (PDF 2976 KB)
