# Supplementary material for: Room temperature stable multitalent: highly reactive and versatile copper guanidine complexes in oxygenation reactions
Source: J Biol Inorg Chem. 2021 Feb 17;26(2):249–63. doi: 10.1007/s00775-021-01849-9 (PMC8068697; doi:10.1007/s00775-021-01849-9)
Supplement: Supplementary file 1 — Supplementary file1 (PDF 2976 KB) [file 775_2021_1849_MOESM1_ESM.pdf]

Supporting Information

Room Temperature Stable Multitalent: Highly Reactive and Versatile Copper Guanidine Complexes in Oxygenation Reactions

Melanie Paul[1], Alexander Hoffmann[1], Sonja Herres-Pawlis*[1]

DOI: 10.1007/XXXXX

Table of content

1 General Remarks 3

2 Characterization of the Copper(I) Precursor Species 4

2.1 Spectra of [Cu(**L1**)I] (**C1a**), [Cu(**L1**)I]·CuI (**C1a**·CuI) and [Cu(**L1**)$_2$]I (**C1b**) 4

2.2 Spectra of [Cu(**L1**)Br] (**C2a**) and [Cu(**L1**)$_2$]Br (**C2b**) 8

2.3 Spectra of [Cu(**L1**)Cl] (**C3a**) and [Cu(**L1**)$_2$]Cl (**C3b**) 11

2.4 Crystallographic Data of [Cu(**L1**)X] (**C1a-C3a**) 14

3 Characterization of Bis(μ-oxido) Dicopper(III) Species 15

3.1 Oxygenation of **C1a** and **C1b** 15

3.2 Oxygenation of **C2a** and **C2b** 16

3.3 Oxygenation of **C3a** and **C3b** 17

3.4 Cryo-UHR-ESI Mass Spectrometry of the Oxygenation Reactions 18

3.5 Titration of [**O1I**]$^+$ with Ligand **L1** 25

3.6 Titration of [**O1I**]$^+$ with Iodide Source Bu_4NI 26

3.7 Titration of [**O1I**]$^+$ with Copper Source $[Cu(MeCN)_4]PF_6$ 27

3.8 Titration of [**O1**]$(PF_6)_2$ with Bromide Source Bu_4NBr 28

3.9 Titration of [**O1**]$(PF_6)_2$ with Chloride Source Bu_4NCl 29

3.10 Stability of [**O1I**](CuI_2) towards H_2O 30

4 Catalytic Reactivity of [**O1I**](CuI_2) 31

4.1 Reaction of [**O1I**](CuI_2) with Phenols 31

5 Control Experiments 32

5.1 Reaction of [**O1I**](CuI_2) with Triethylamine 32

5.2 Reaction of [**O1I**](CuI_2) with 1,2-Phenylenediamine 33

5.3 Reaction of Copper Iodide with 2-Naphthol in the Presence of O_2 and Subsequent Reaction with 1,2-Phenylenediamine 34

6 TD-DFT Calculations on [**O1**]$^{2+}$ and [**O1I**]$^+$ 35

7 References 41

1 General Remarks

All chemicals were purchased commercially (Table S1) and used without further purification unless otherwise noted.

Table S1: Used chemicals.

chemicals	supplier
1,2-phenylenediamine	Fluka
2-naphthol	Sigma-Aldrich
3-quinolinol	abcr
4-methoxy phenol	Sigma-Aldrich
5-indolol	abcr
6-quinolinol	Sigma-Aldrich
7-indolol	abcr
acetonitrile-d_3	Sigma-Aldrich
benzophenone	Alfa Aesar
calcium hydride (0-2 mm)	Acros Organics
copper(I) bromide	Fluka
copper(II) chloride	Fluka
copper(I) iodide	Fluka
dimethyl sulfoxide-d_6	Sigma-Aldrich
ethylenediaminetetraacetic acid (EDTA)	Fluka
hydrochloric acid	Fisher Scientific
sodium	Sigma-Aldrich
sodium sulfate	Grüssing
tetrabutylammonium bromide	abcr
tetrabutylammonium chloride	abcr
tetrabutylammonium iodide	abcr
triethylamine	abcr

2 Characterization of the Copper(I) Precursor Species

2.1 Spectra of [Cu(L1)I] (C1a), [Cu(L1)I]·CuI (C1a·CuI) and [Cu(L1)$_2$]I (C1b)

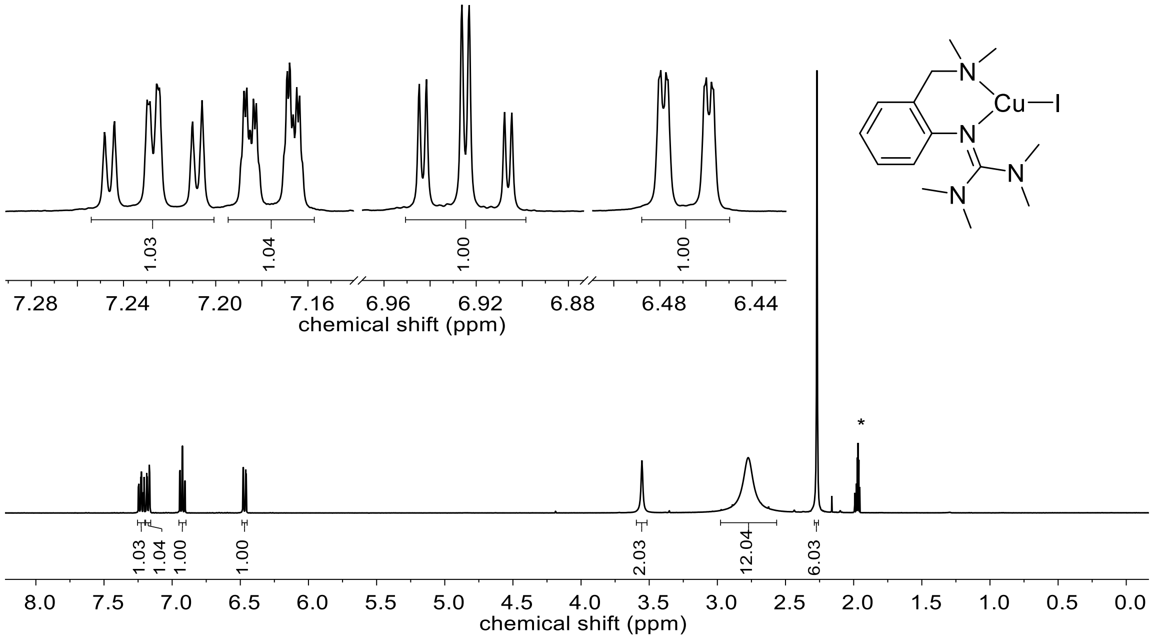

Figure S1: ^{1}H NMR spectrum of [Cu(**L1**)I] (**C1a**) (* = CD_3CN).

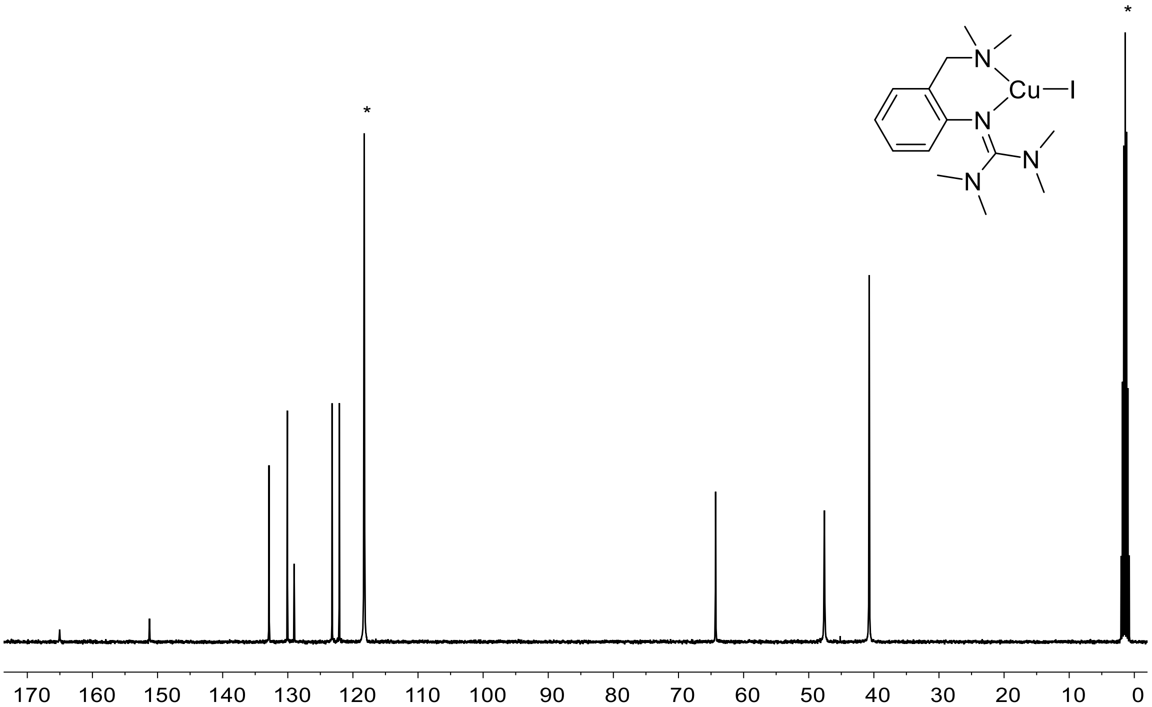

Figure S2: $^{13}C\{^{1}H\}$ NMR spectrum of [Cu(**L1**)I] (**C1a**) (* = CD_3CN).

Additional information on the NMR spectra of the target compound **C1a** including original data files is available *via* Chemotion Repository: https://dx.doi.org/10.14272/DTVVDHUKUBSTJW-UHFFFAOYSA-M.1

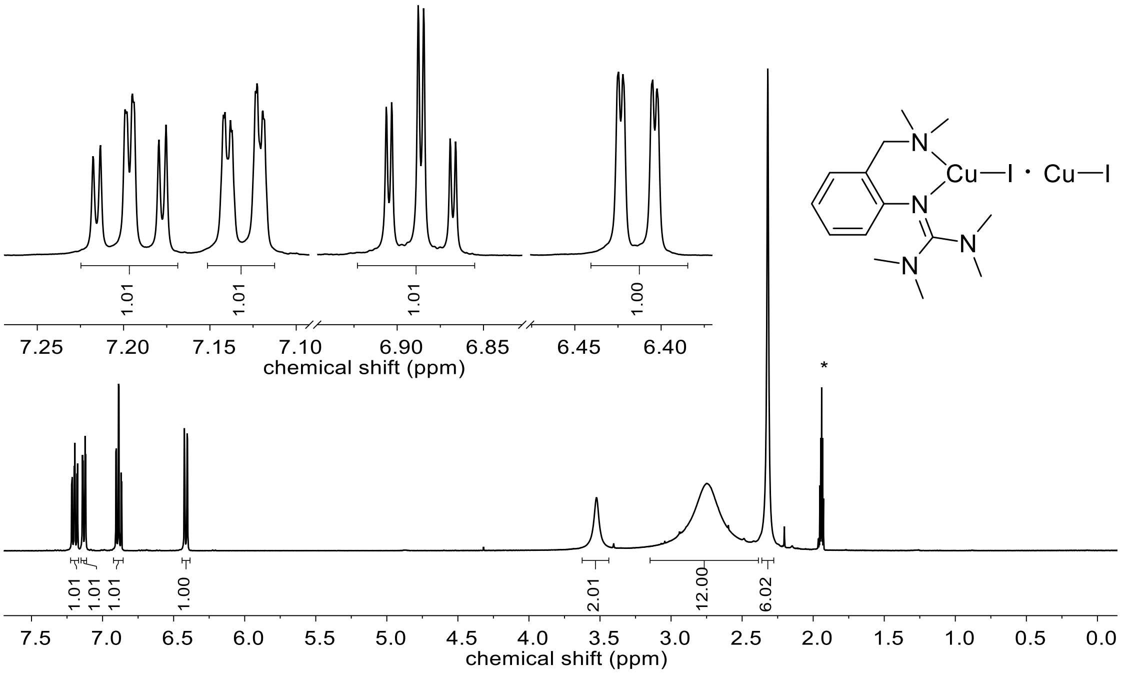

Figure S3: ^{1}H NMR spectrum of [Cu(**L1**)I]•CuI (**C1a**•CuI) (* = CD_3CN).

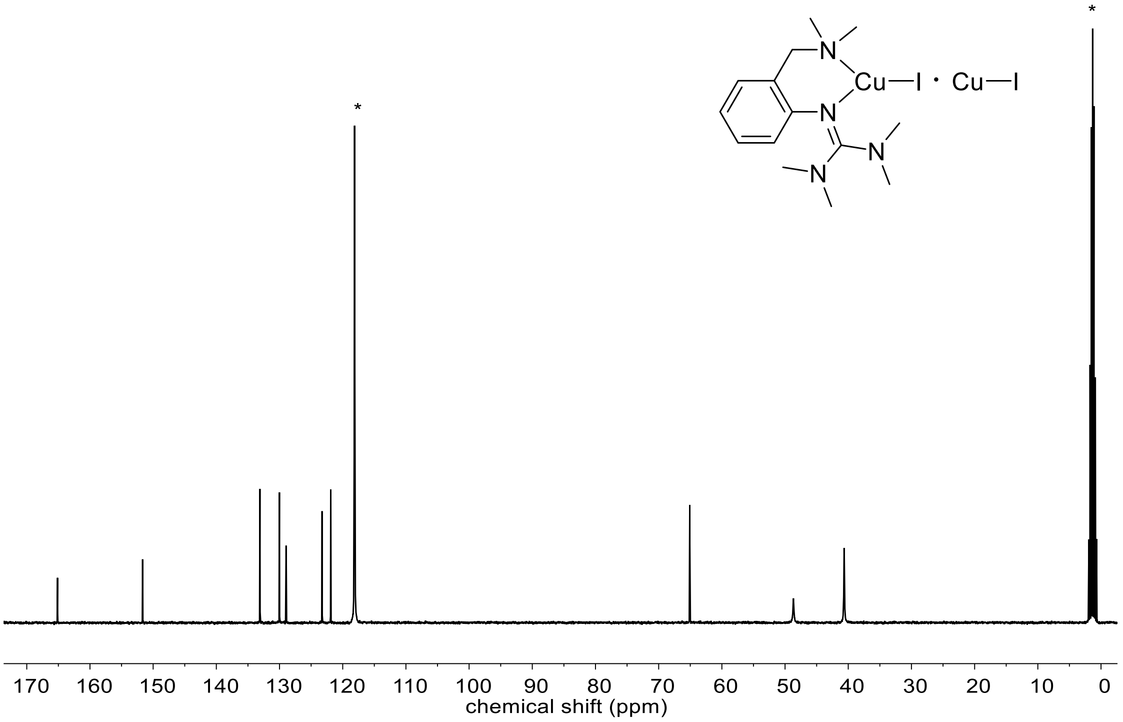

Figure S4: ^{13}C NMR spectrum of [Cu(**L1**)I]•CuI (**C1a**•CuI) (* = CD_3CN).

Additional information on the NMR spectra of the target compound **C1a**•CuI including original data files is available *via* Chemotion Repository: https://dx.doi.org/10.14272/XJVUTLXAFQMECW-UHFFFAOYSA-L.1

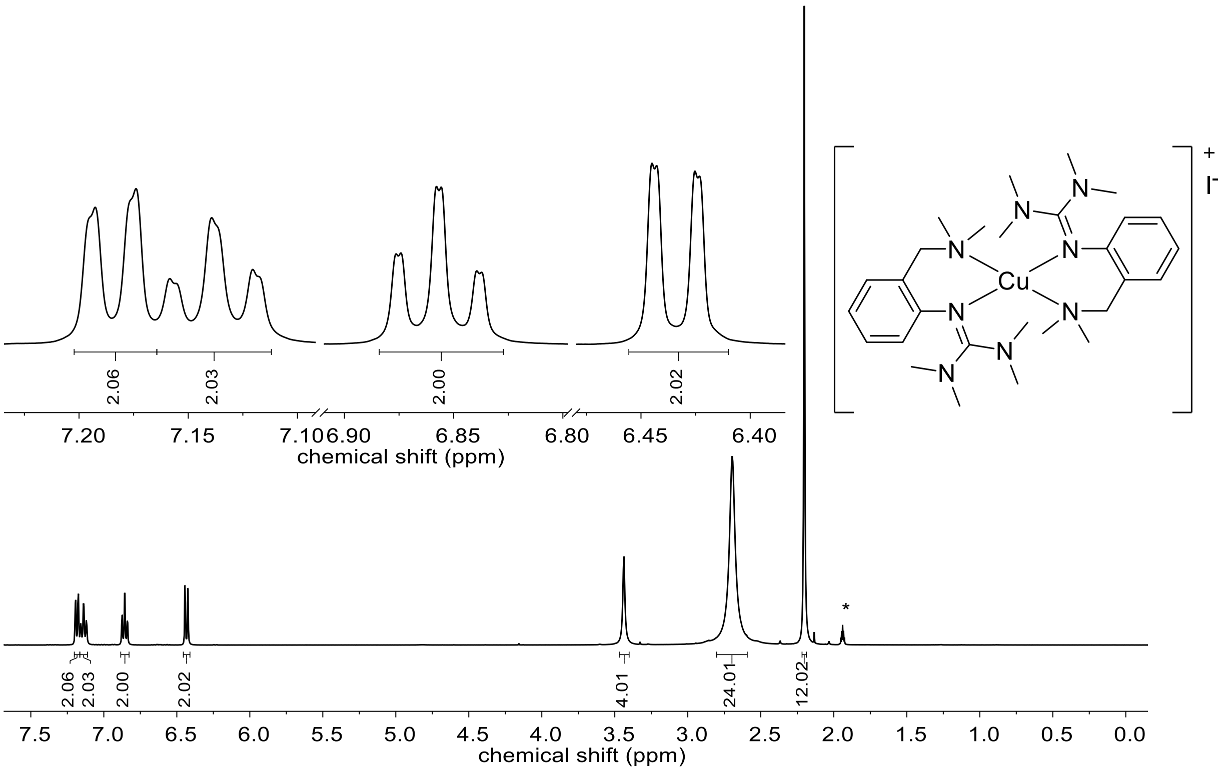

Figure S5: ^{1}H NMR spectrum of [Cu(**L1**)$_2$]I (**C1b**) (* = CD_3CN).

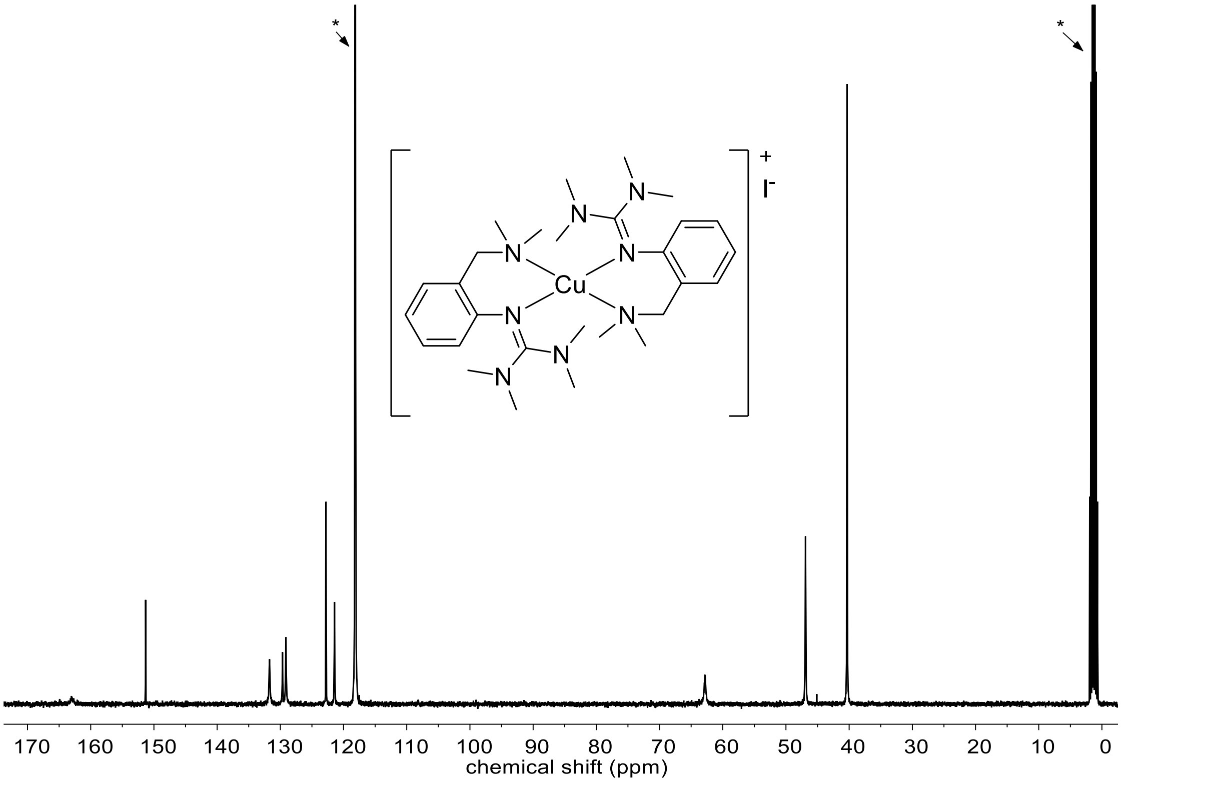

Figure S6: ^{13}C NMR spectrum of [Cu(**L1**)$_2$]I (**C1b**) (* = CD_3CN).

Additional information on the NMR spectra of the target compound **C1b** including original data files is available *via* Chemotion Repository: https://dx.doi.org/10.14272/VGPGJDRLIBJZAR-UHFFFAOYSA-M.1

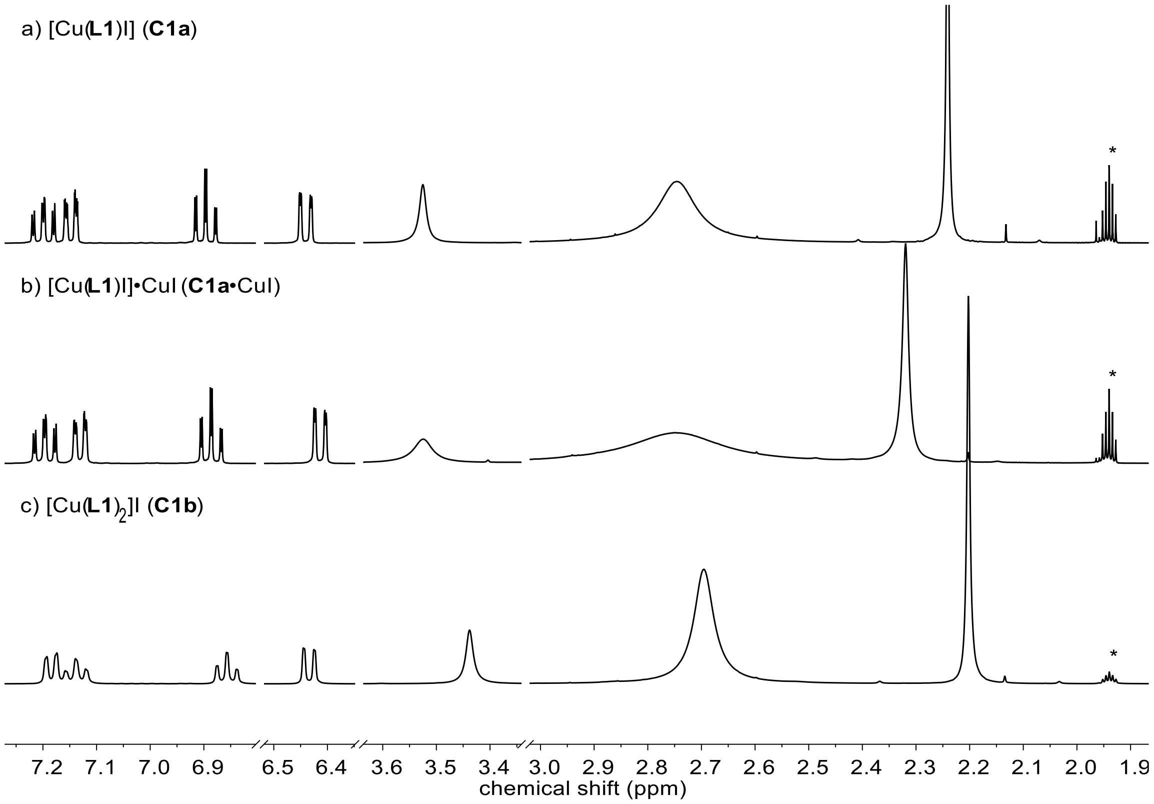

Figure S7: ^{1}H NMR spectra of a) [Cu(**L1**)I] (**C1a**), b) [Cu(**L1**)I]•CuI (**C1a**•CuI) and c) [Cu(**L1**)$_2$]I (**C1b**) (* = CD_3CN).

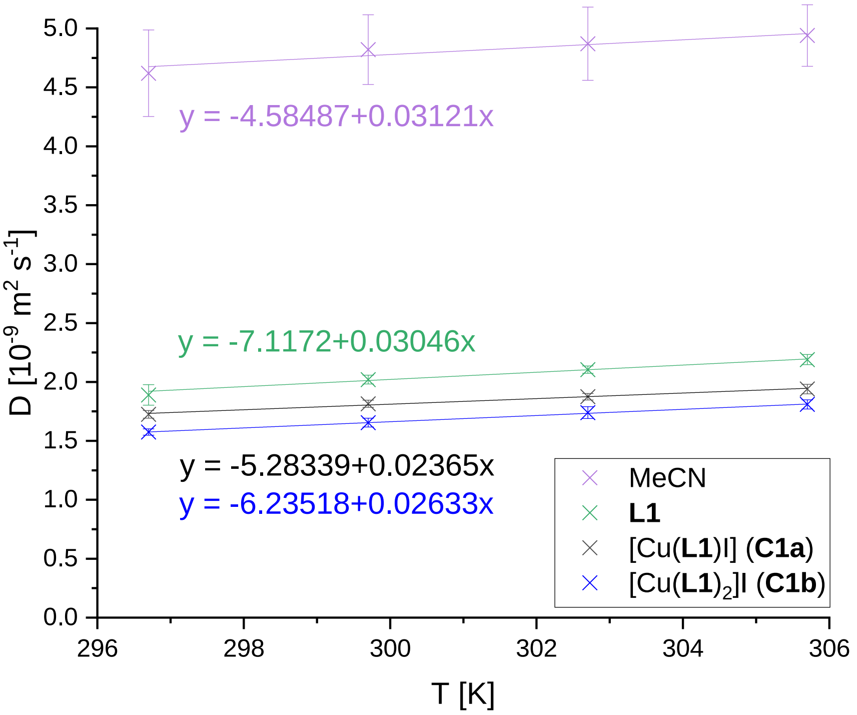

Figure S8: Diffusion constant D of acetonitrile (violet), **L1** (green), monochelate complex **C1a** (black) and bischelate complex **C1b** (blue) in dependence of temperature T (derived from ^{1}H DOSY NMR spectra).

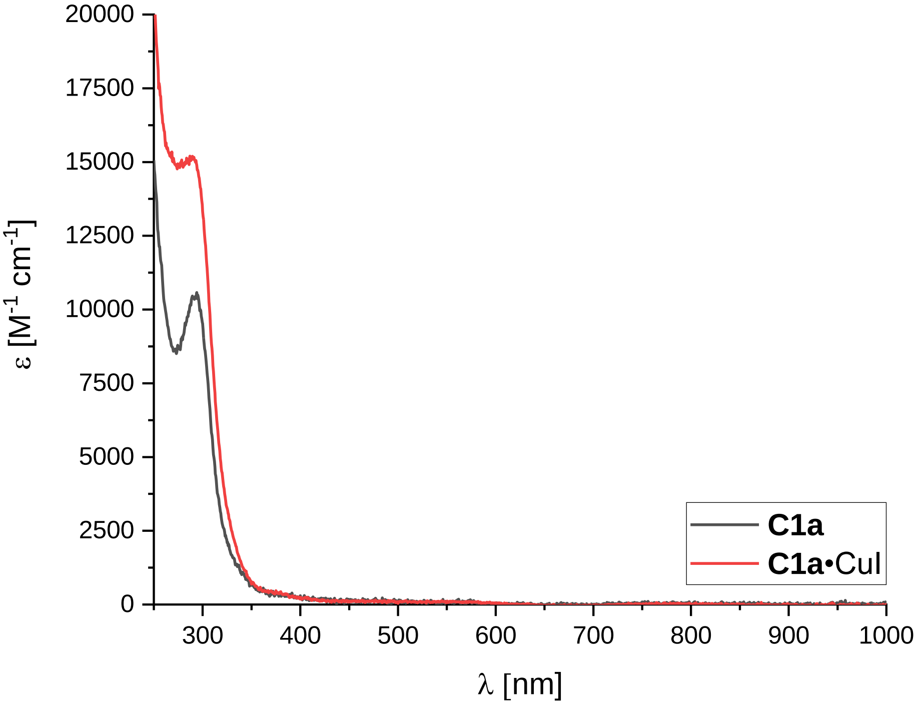

Figure S9: UV/Vis spectra of [Cu(**L1**)I] (**C1a**, black) and [Cu(**L1**)I]•CuI (**C1a**•CuI, red) in tetrahydrofuran at room temperature.

2.2 Spectra of [Cu(L1)Br] (C2a) and [Cu(L1)$_2$]Br (C2b)

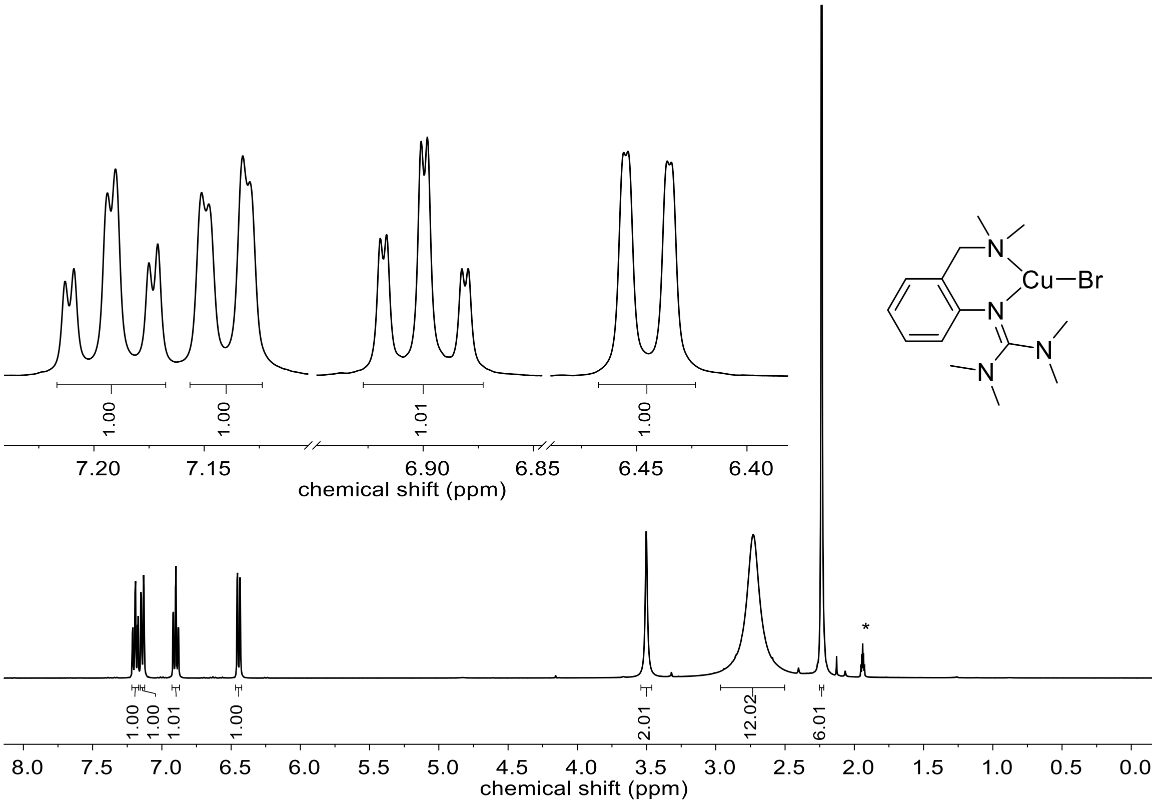

Figure S10: ^{1}H NMR spectrum of [Cu(**L1**)Br] (**C2a**) (* = CD_3CN).

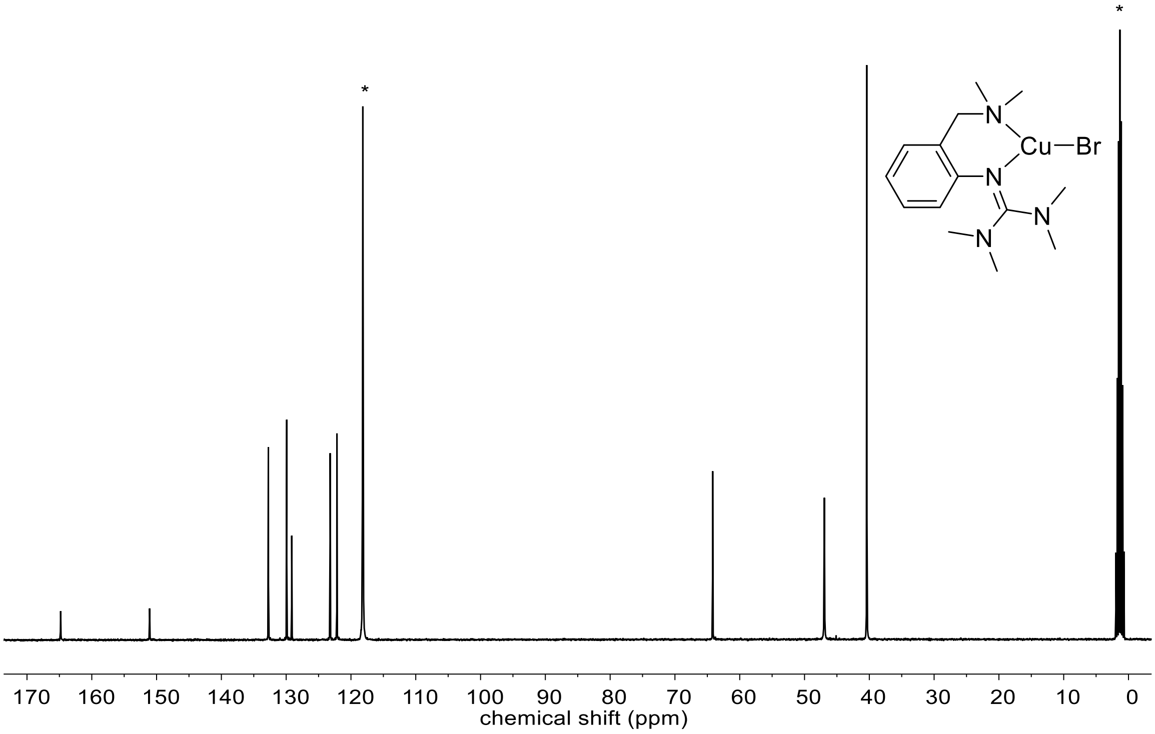

Figure S11: $^{13}C\{^{1}H\}$ NMR spectrum of [Cu(**L1**)Br] (**C2a**) (* = CD_3CN).

Additional information on the NMR spectra of the target compound **C2a** including original data files is available *via* Chemotion Repository: https://dx.doi.org/10.14272/XYBUOSNKFWKMII-UHFFFAOYSA-M.1

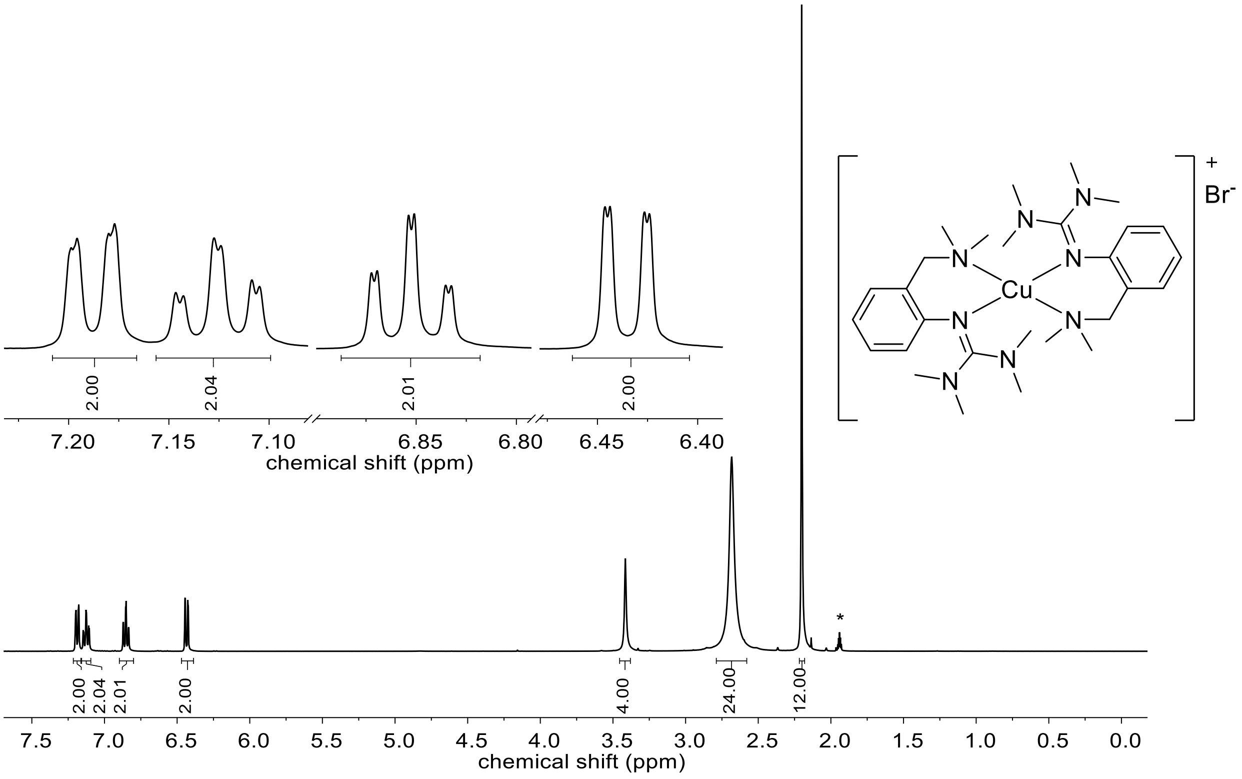

Figure S12: ^{1}H NMR spectrum of $[Cu(\mathbf{L1})_2]Br$ (**C2b**) (* = CD_3CN).

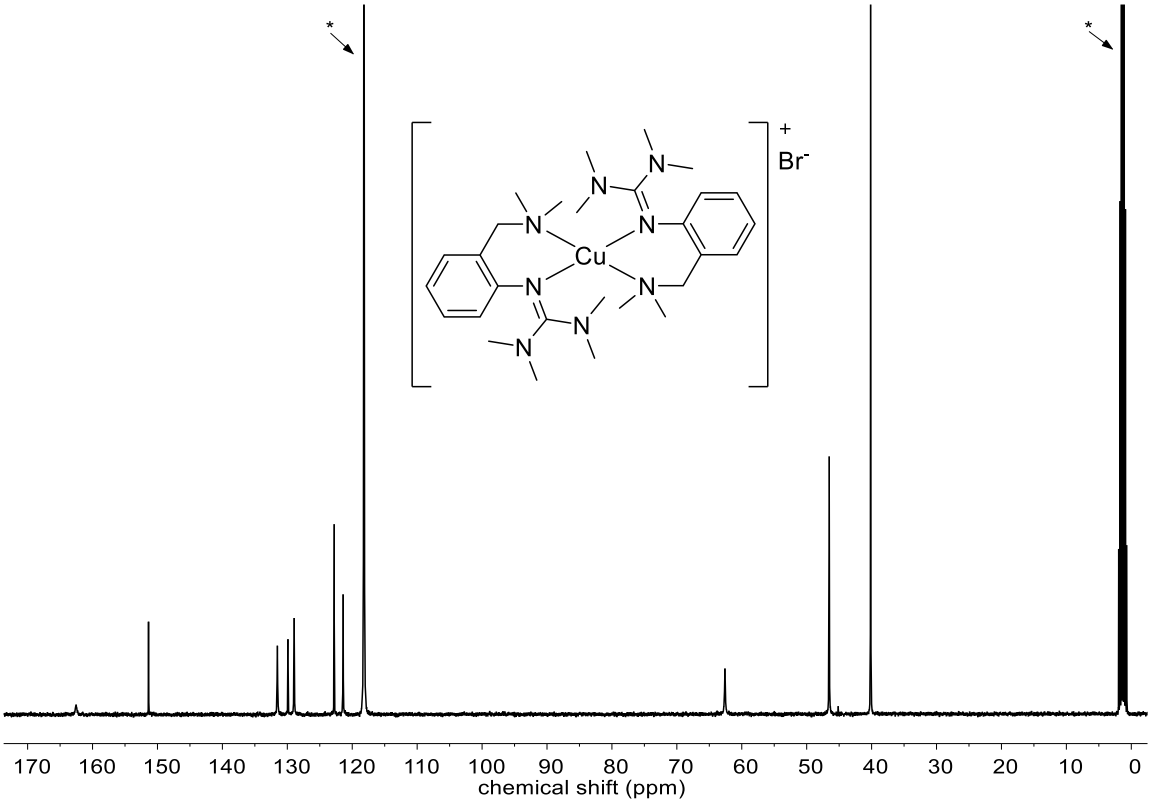

Figure S13: $^{13}C\{^{1}H\}$ NMR spectrum of [Cu(**L1**)$_2$]Br (**C2b**) (* = CD_3CN).

Additional information on the NMR spectra of the target compound **C2b** including original data files is available *via* Chemotion Repository: https://dx.doi.org/10.14272/GGYHSQSQRIVBSO-UHFFFAOYSA-M.1

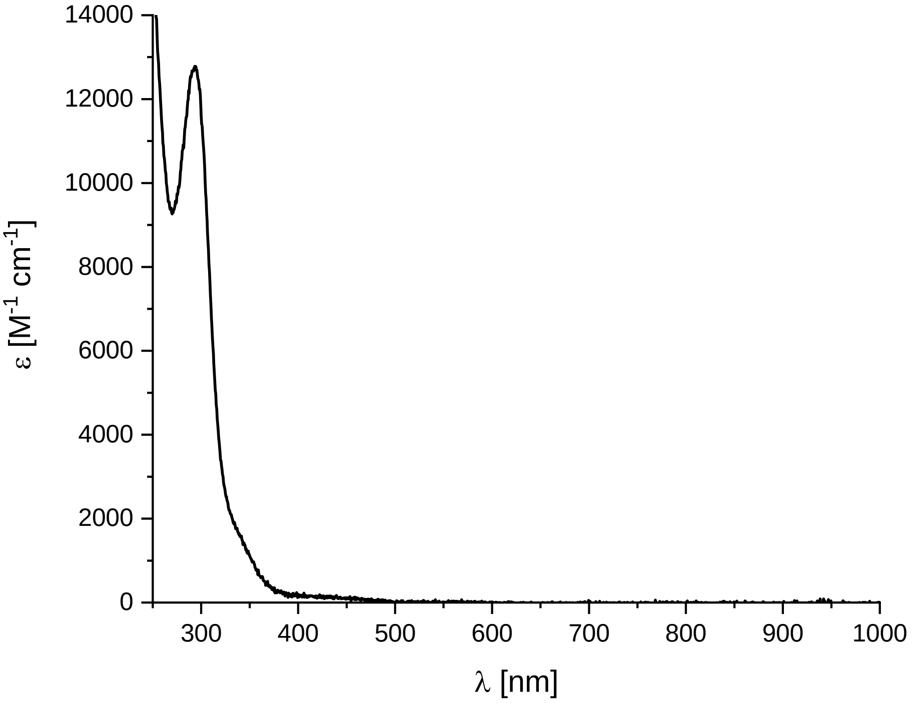

Figure S14: UV/Vis spectrum of [Cu(**L1**)Br] (**C2a**) in tetrahydrofuran at room temperature.

2.3 Spectra of [Cu(L1)Cl] (C3a) and [Cu(L1)₂]Cl (C3b)

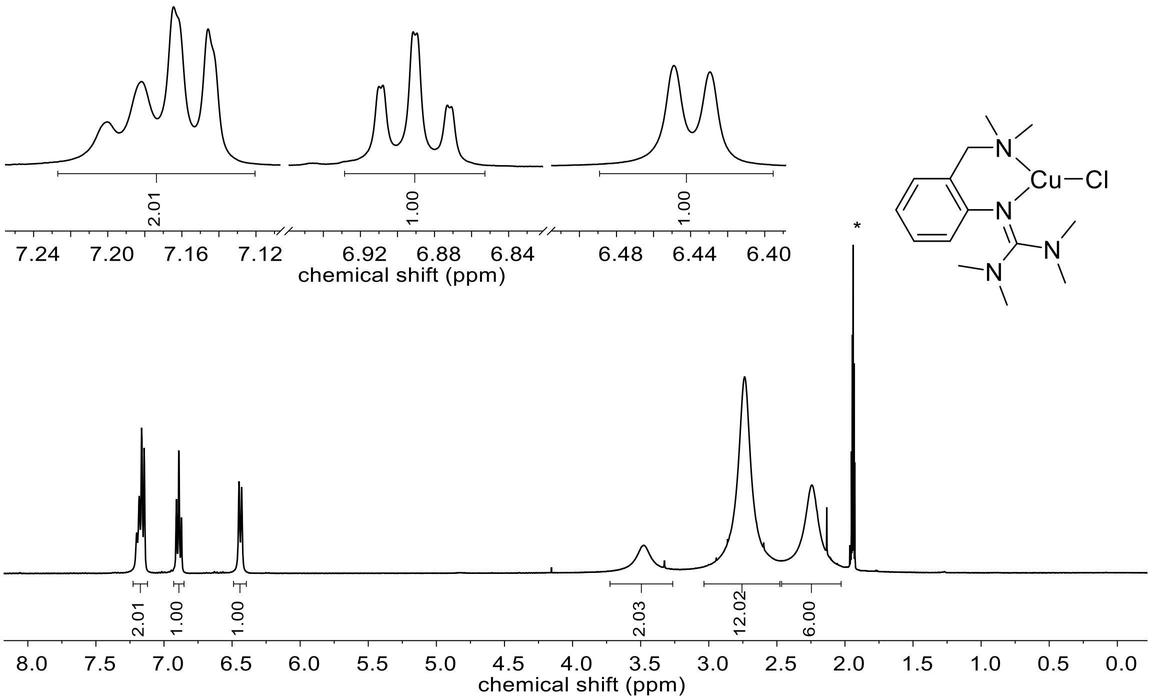

Figure S15: ^{1}H NMR spectrum of [Cu(**L1**)Cl] (**C3a**) (* = CD_3CN).

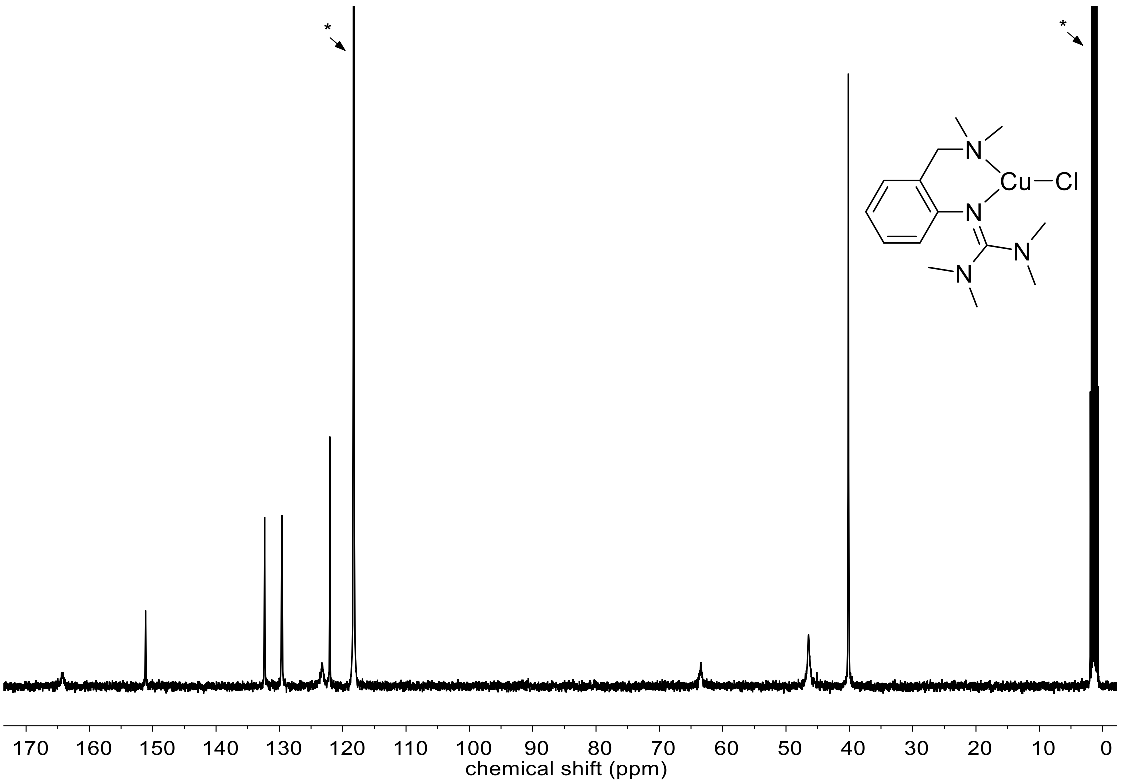

Figure S16: $^{13}C\{^1H\}$ NMR spectrum of [Cu(**L1**)Cl] (**C3a**) (* = CD_3CN).

Additional information on the NMR spectra of the target compound **C3a** including original data files is available *via* Chemotion Repository: https://dx.doi.org/10.14272/DHTDCYWQWFEGRN-UHFFFAOYSA-M.1

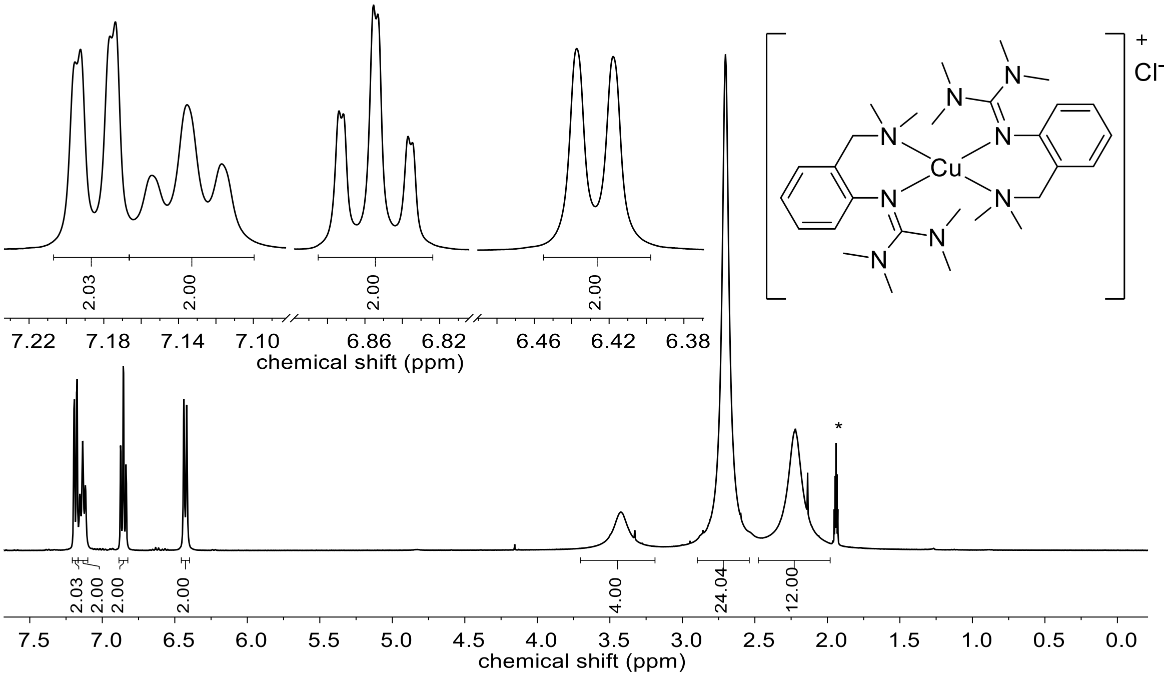

Figure S17: ^{1}H NMR spectrum of [Cu(**L1**)$_2$]Cl (**C3b**) (* = CD_3CN).

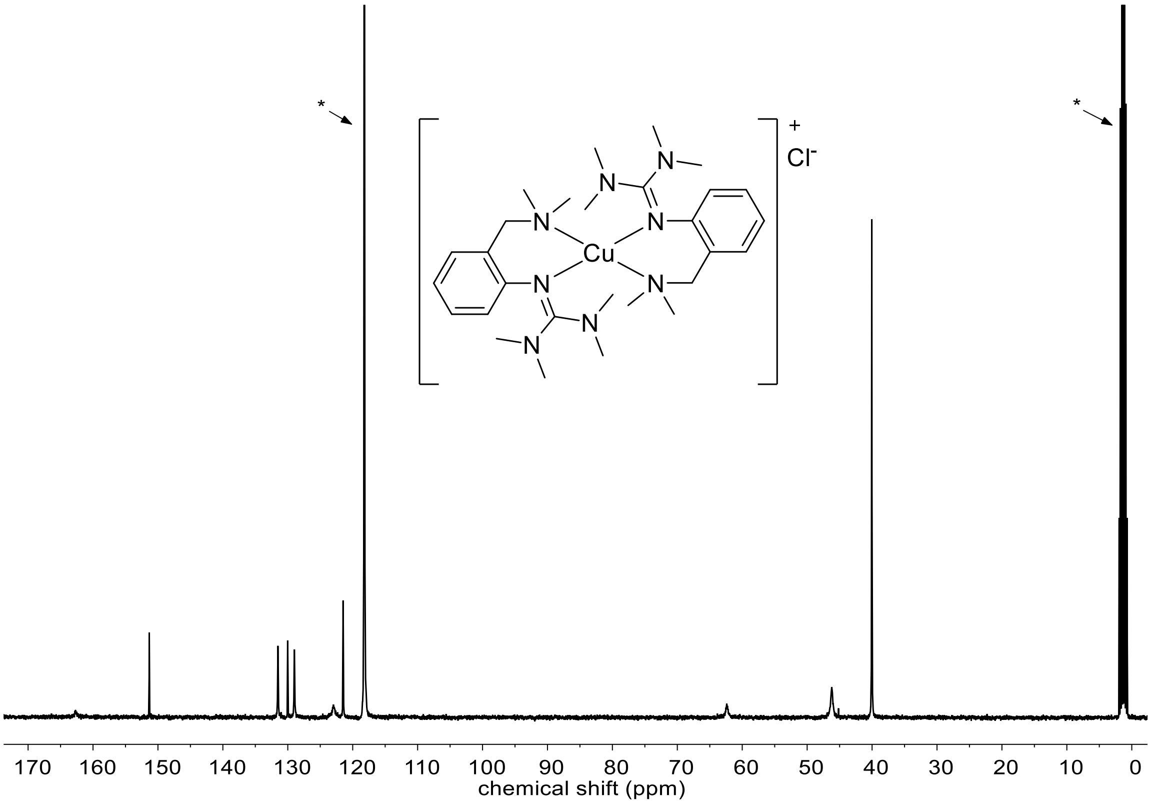

Figure S18: $^{13}C\{^{1}H\}$ NMR spectrum of [Cu(**L1**)$_2$]Cl (**C3b**) (* = CD_3CN).

Additional information on the NMR spectra of the target compound **C3b** including original data files is available *via* Chemotion Repository: https://dx.doi.org/10.14272/GYQYOCAOCTTZAK-UHFFFAOYSA-M.1

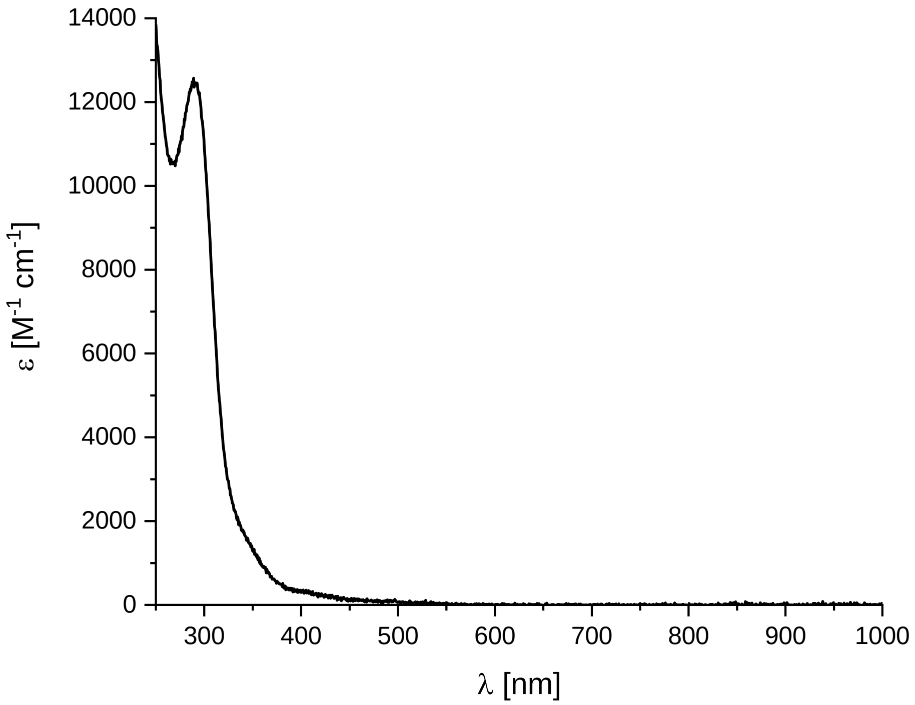

Figure S19: UV/Vis spectrum of [Cu(**L1**)Cl] (**C3a**) in tetrahydrofuran at room temperature.

2.4 Crystallographic Data of [Cu(L1)X] (C1a-C3a)

Table S2: Crystallographic data and parameters of **C1a-C3a**.

	**[Cu(L1)I] (C1a)**	**[Cu(L1)Br](C2a)**	**[Cu(L1)Cl] (C3a)**
Empirical formula	$C_{14}H_{24}CuIN_4$	$C_{14}H_{24}CuBrN_4$	$C_{14}H_{24}CuClN_4$
Formula weight [g mol^{-1}]	438.81	391.82	347.36
T [K]	100	100	100
λ [Å]	0.71073	0.71073	0.71073
Crystal system	Orthorhombic	Orthorhombic	Orthorhombic
Space group	Pbca	Pbca	Pbca
a [Å]	16.4039(17)	16.576(3)	16.690(3)
b [Å]	10.8976(11)	10.647(2)	10.502(2)
c [Å]	19.449(2)	18.781(4)	18.476(4)
α [°]	90	90	90
β [°]	90	90	90
γ [°]	90	90	90
V [Å^3]	3476.8(6)	3314.6(11)	3238.6(11)
Z	8	8	8
ρ_{calc} [g cm^{-3}]	1.677	1.570	1.425
μ [mm^{-1}]	3.029	3.725	1.510
F(000)	1744	1600	1456
Crystal size [mm]	0.220 x 0.210 x 0.060	0.260 x 0.160 x 0.080	0.150 x 0.120 x 0.080
hkl range	±22, ±14, ±26	$-22 \leq h \leq 14$, $-13 \leq k \leq 14$, ±25	$-22 \leq h \leq 15$, $-11 \leq k \leq 14$, $-23 \leq l \leq 24$
Reflections collected	69448	16502	13638
Independent reflections	4549	4053	3967
R_{int}	0.0743	0.0631	0.0531
Number of parameters	187	187	187
R_1 [I≥2σ(I)]	0.0306	0.0455	0.0365
wR_2 (all data)	0.0808	0.0942	0.0732
Goodness-of-fit	1.014	1.002	0.916
Largest diff. peak hole [eÅ^{-3}]	0.733, –0.868	0.813, –0.495	0.373, –0.331
CCDC	2003620	2003621	2003622

3 Characterization of Bis(μ-oxido) Dicopper(III) Species

3.1 Oxygenation of C1a and C1b

Complex **C1a** was oxygenated to give [**O1I**]$^{+}$ (0.5 mM) according to the protocol described in the manuscript at -80 °C and analyzed via UV/Vis spectroscopy (Figure S20).

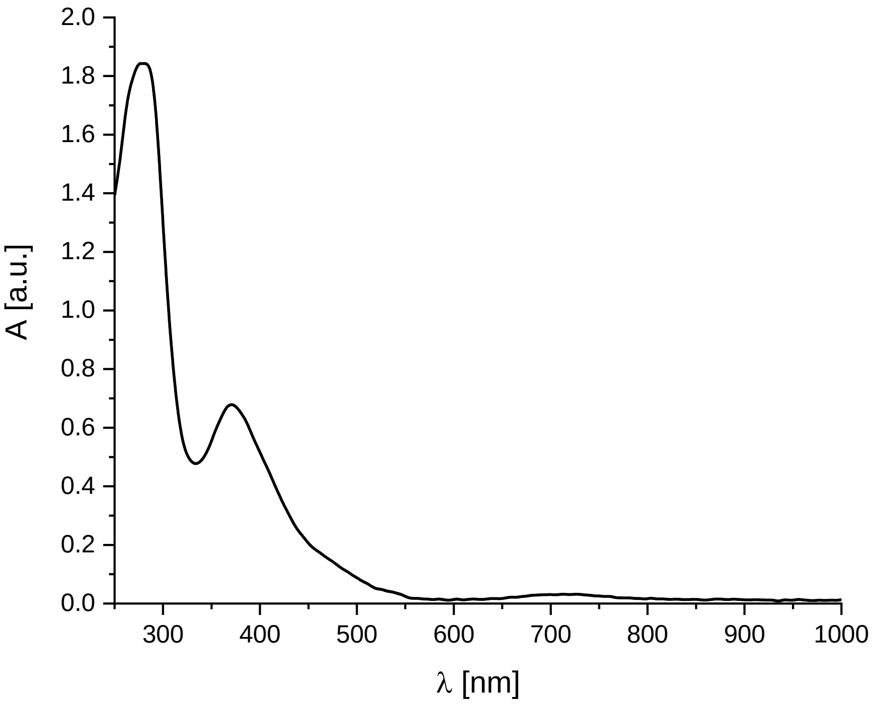

Figure S20: UV/Vis spectrum of the oxygenation of **C1a** in tetrahydrofuran (0.5 mM) at -80 °C after two hours.

The reddish-brown reaction solution revealed two absorption bands at 370 nm and 290 nm, which were stable at -80 °C for at least 6 h. The absorption bands represent a quantity of the formed bis(μ-oxido) complex of approximately 65%.

Oxygenation of bischelate complex **C1b** led to a similar UV/Vis spectrum of the reddish-brown species, except the intensity of the absorption bands is significantly lower revealing only 43% formation of the bis(μ-oxido) complex.

3.2 Oxygenation of C2a and C2b

Complex **C2a** was oxygenated to give [**O1**]Br_2 (0.5 mM) according to the protocol described in the manuscript at -100 °C and analyzed via UV/Vis spectroscopy (Figure S21).

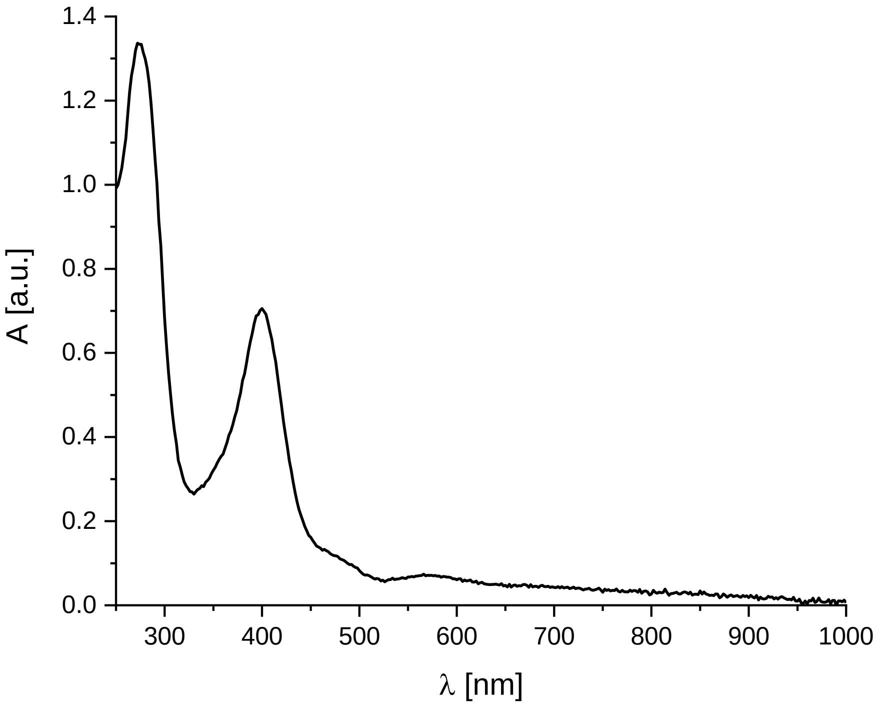

Figure S21: UV/Vis spectrum of the oxygenation of **C2a** in tetrahydrofuran (0.5 mM) at -100 °C after 33 s.

After the addition of **C2a** complex solution, an immediate color change to green was observed, which is similar to [**O1**]$(PF_6)_2$.[1] Absorption bands at 399 nm and 270 nm, documenting 70% quantity of [**O1**]Br_2, were formed immediately at -100 °C and decayed completely within a few minutes. Here, no full formation of the bis(µ-oxido) species could be observed due a faster decay rate compared to the formation rate.

Oxygenation of bischelate complex **C2b** led to a similar UV/Vis spectrum of the greenish species, except the intensity of the absorption bands is significantly lower revealing only 36% formation of the bis(µ-oxido) complex.

3.3 Oxygenation of C3a and C3b

Complex **C3a** was oxygenated to give [**O1**]Cl_2 (0.5 mM) according to the protocol described in the manuscript at -100 °C and analyzed via UV/Vis spectroscopy (Figure S22).

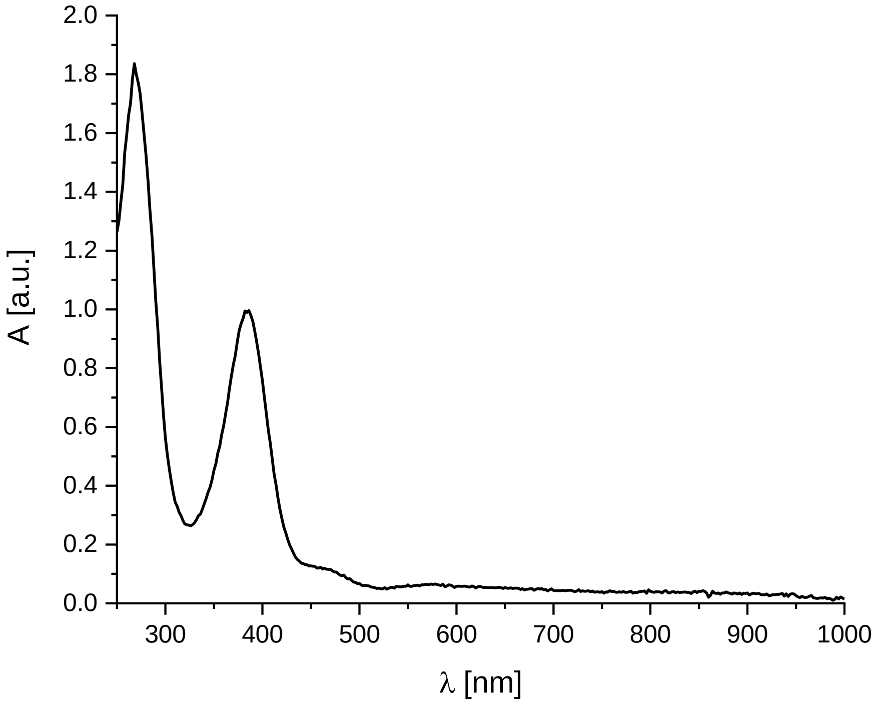

Figure S22: UV/Vis spectrum of the oxygenation of **C3a** in tetrahydrofuran (0.5 mM) at -100 °C after 45 s.

After the addition of **C3a** complex solution, an immediate color change to green was observed, which is similar to [**O1**]$(PF_6)_2$.[1] Absorption bands at 386 nm (21000 M^{-1} cm^{-1}) and 270 nm (50000 M^{-1} cm^{-1}) were formed immediately at -100 °C and decayed quickly afterwards within minutes.

Oxygenation of bischelate complex **C3b** led to a similar UV/Vis spectrum of the greenish species, except the intensity of the absorption bands is significantly lower revealing only 50% formation of the bis(μ-oxido) complex.

3.4 Cryo-UHR-ESI Mass Spectrometry of the Oxygenation Reactions

The respective bis(μ-oxido) species (0.5 mM) was synthesized according to the protocol described in the manuscript and analyzed *via* Cryo-UHR-ESI mass spectrometry.

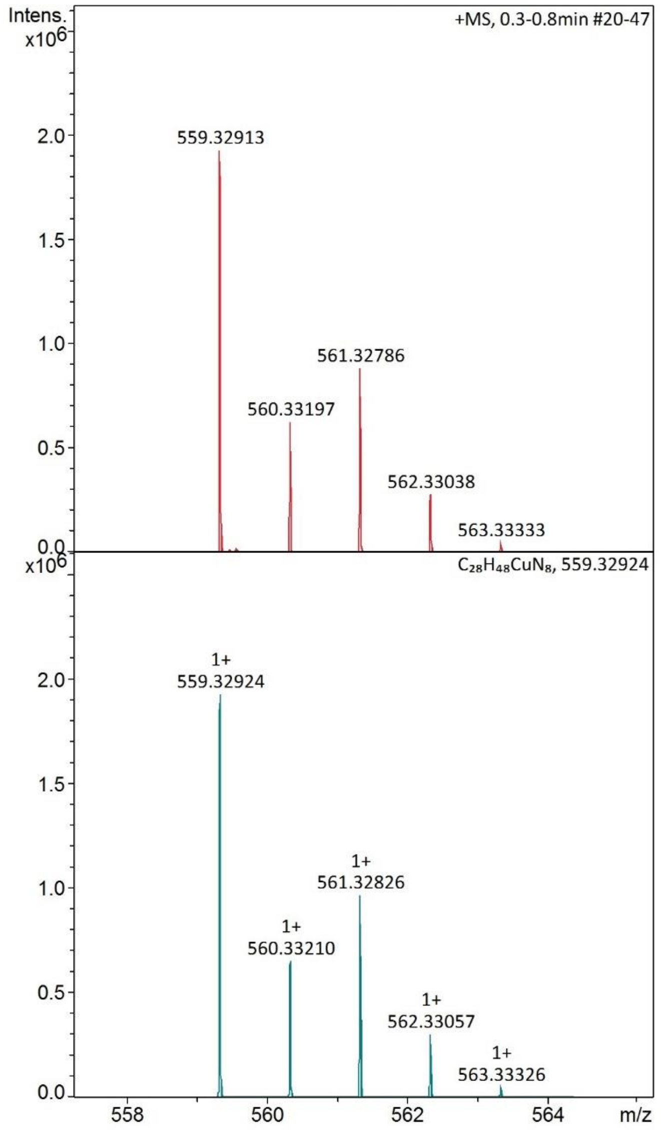

Figure S23: Cryo-UHR-ESI mass spectrometry of [(**L1**)Cu(**L1**)]$^+$ in tetrahydrofuran at –100 °C (top: experimental, bottom: calculated). The isotopic pattern and corresponding m/z value exhibit the mass spectrum of the bischelate complex [(**L1**)Cu(**L1**)]$^+$, which was observed in the oxygenation of **C1a**, **C2a** and **C3a**.

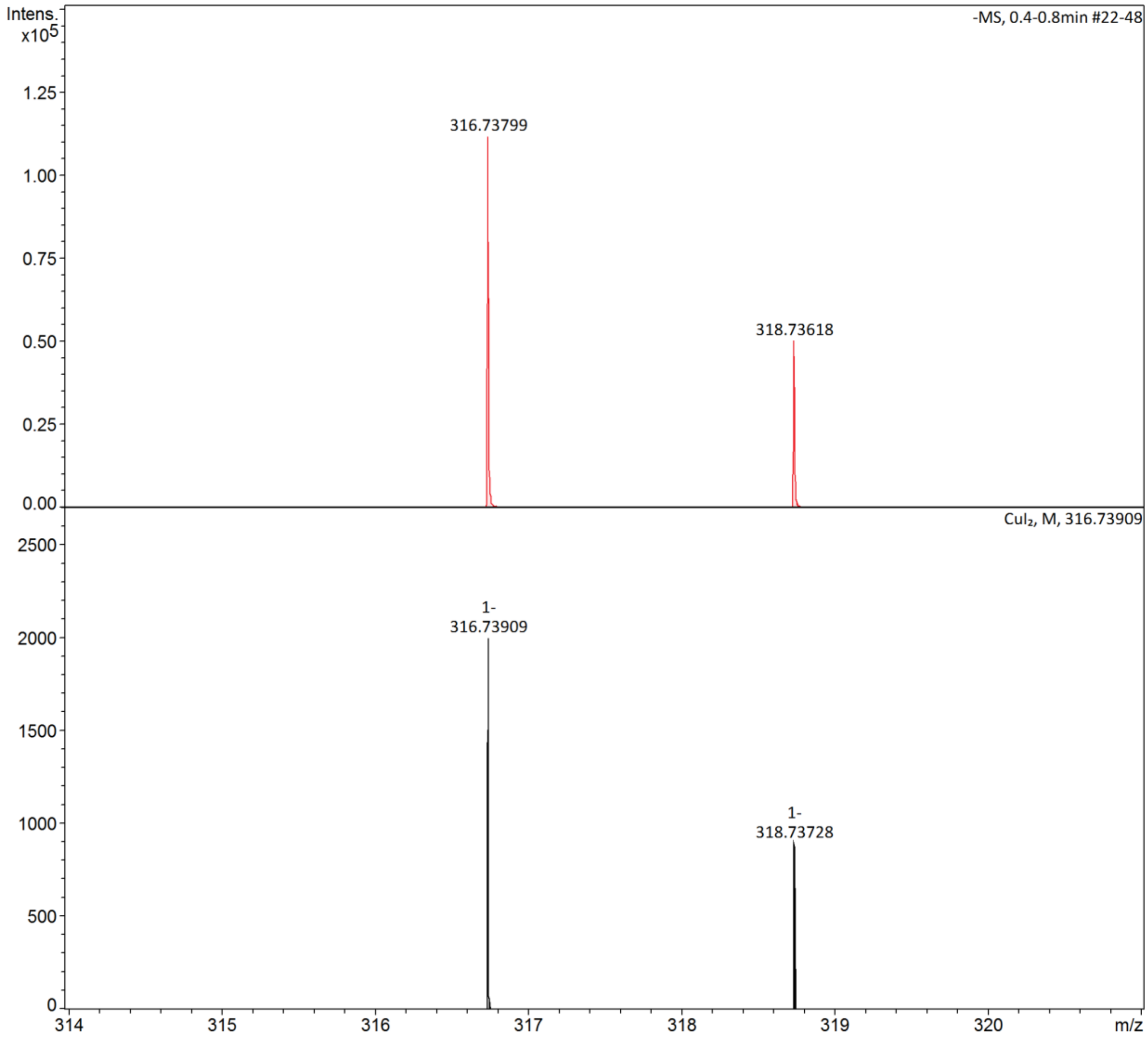

Figure S24: Cryo-UHR-ESI mass spectrometry in the negative mode of the oxygenation reaction of **C1a** as well as **C1a**•CuI in tetrahydrofuran at –100 °C (top: experimental, bottom: calculated). The isotopic pattern and corresponding m/z value exhibit the mass spectrum of the iodidocuprate $[CuI_2]^-$.

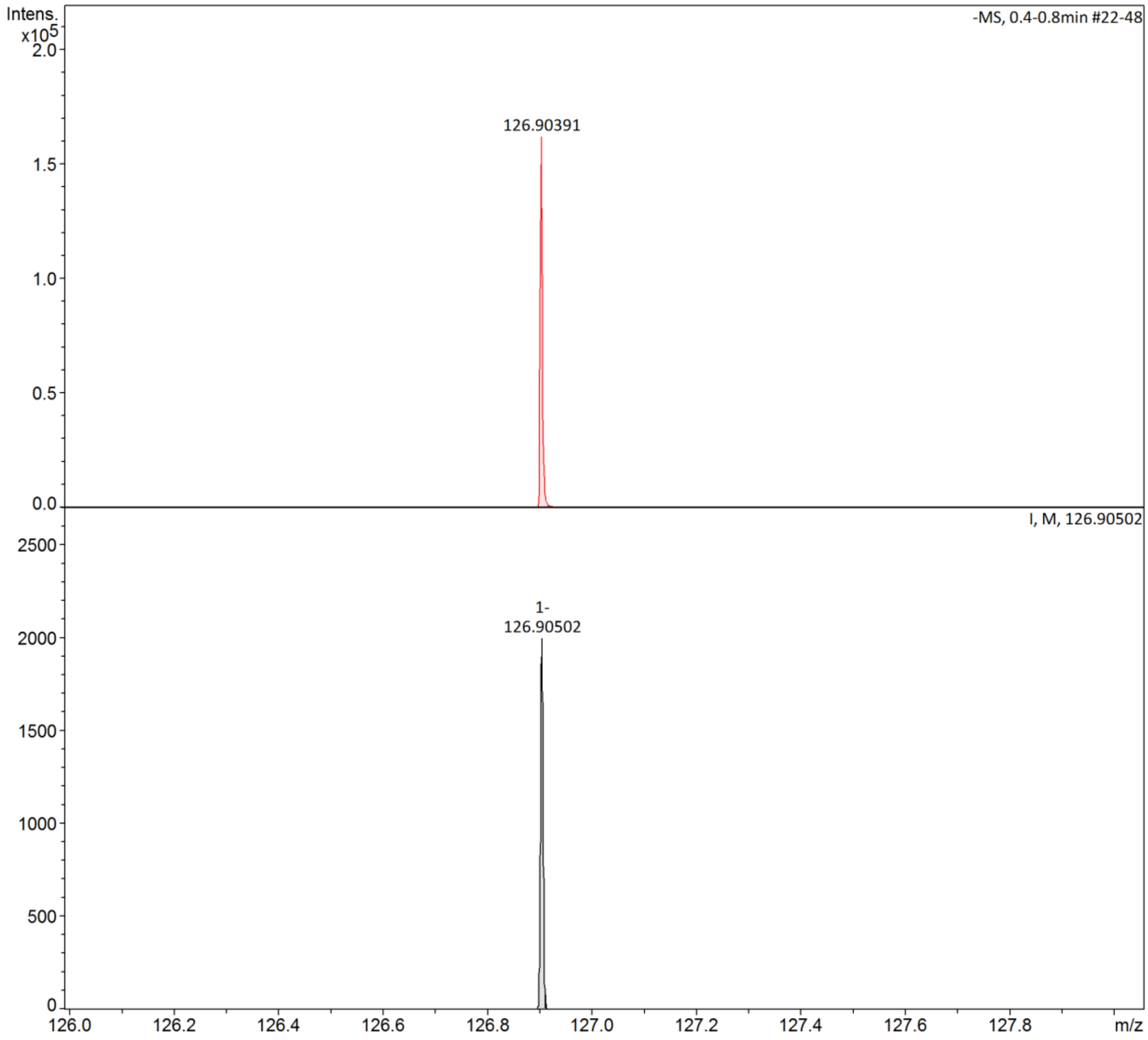

Figure S25: Cryo-UHR-ESI mass spectrometry in the negative mode of the oxygenation reaction of **C1a** as well as **C1a**•CuI in tetrahydrofuran at –100 °C (top: experimental, bottom: calculated). The isotopic pattern and corresponding m/z value exhibit the mass spectrum of the iodide I^-.

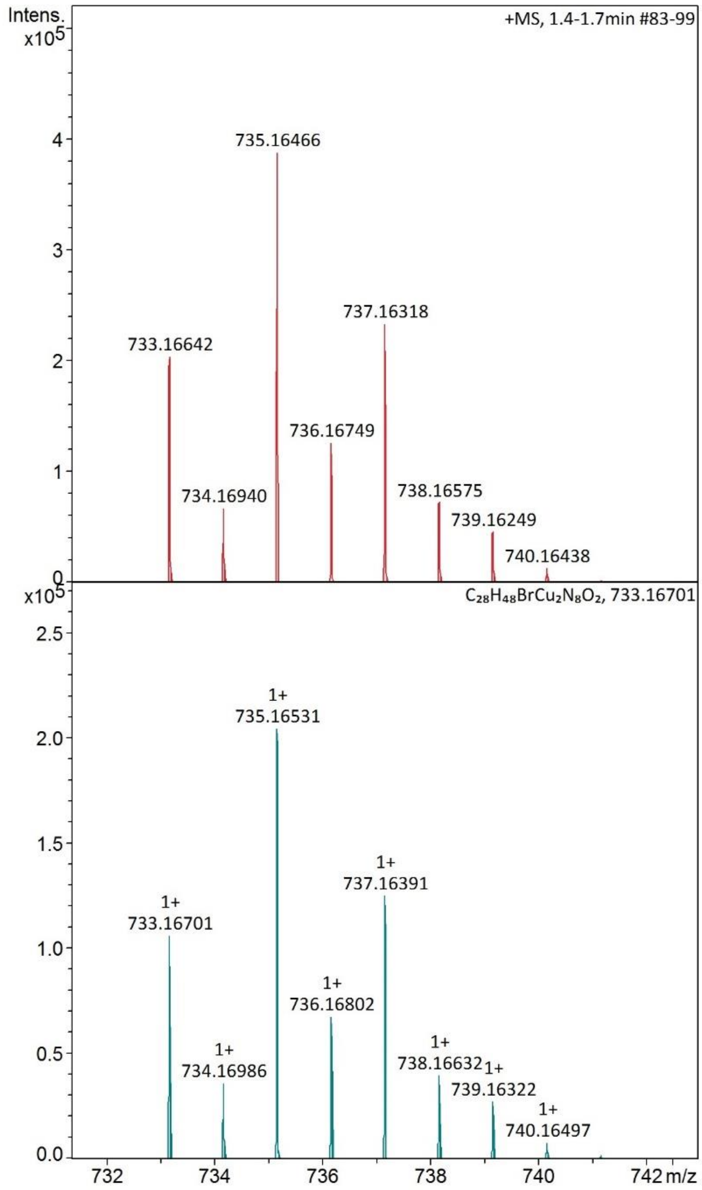

Figure S26: Cryo-UHR-ESI mass spectrometry of [**O1**](Br)$^+$ in tetrahydrofuran at –100 °C (top: experimental, bottom: calculated). The isotopic pattern and corresponding m/z value exhibit the mass spectrum of the monocationic species [**O1**](Br)$^+$, which was observed in the oxygenation of **C2a**.

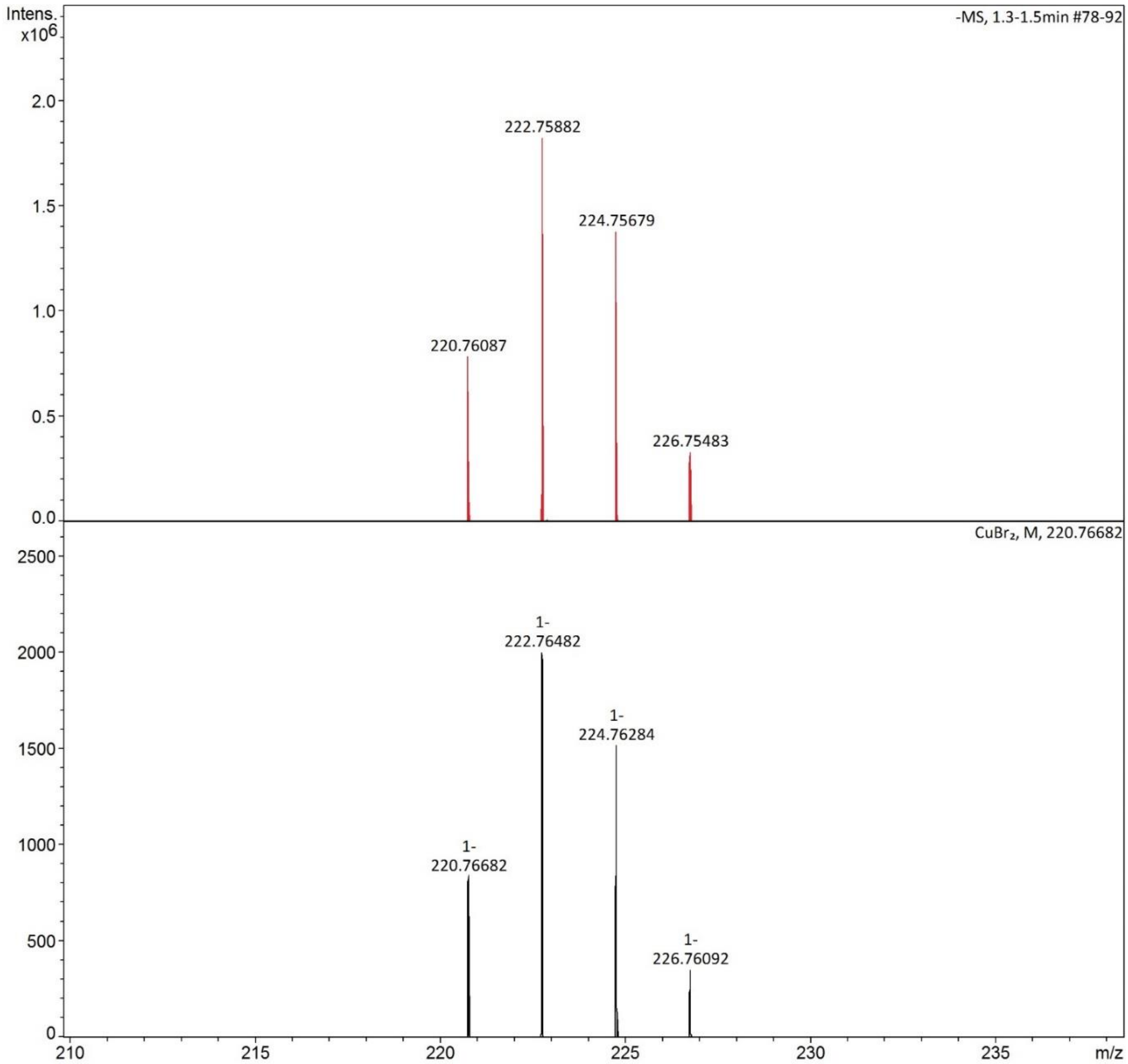

Figure S27: Cryo-UHR-ESI mass spectrometry in the negative mode of the oxygenation reaction of **C2a** in tetrahydrofuran at –100 °C (top: experimental, bottom: calculated). The isotopic pattern and corresponding m/z value exhibit the mass spectrum of the bromidocuprate $[CuBr_2]^-$.

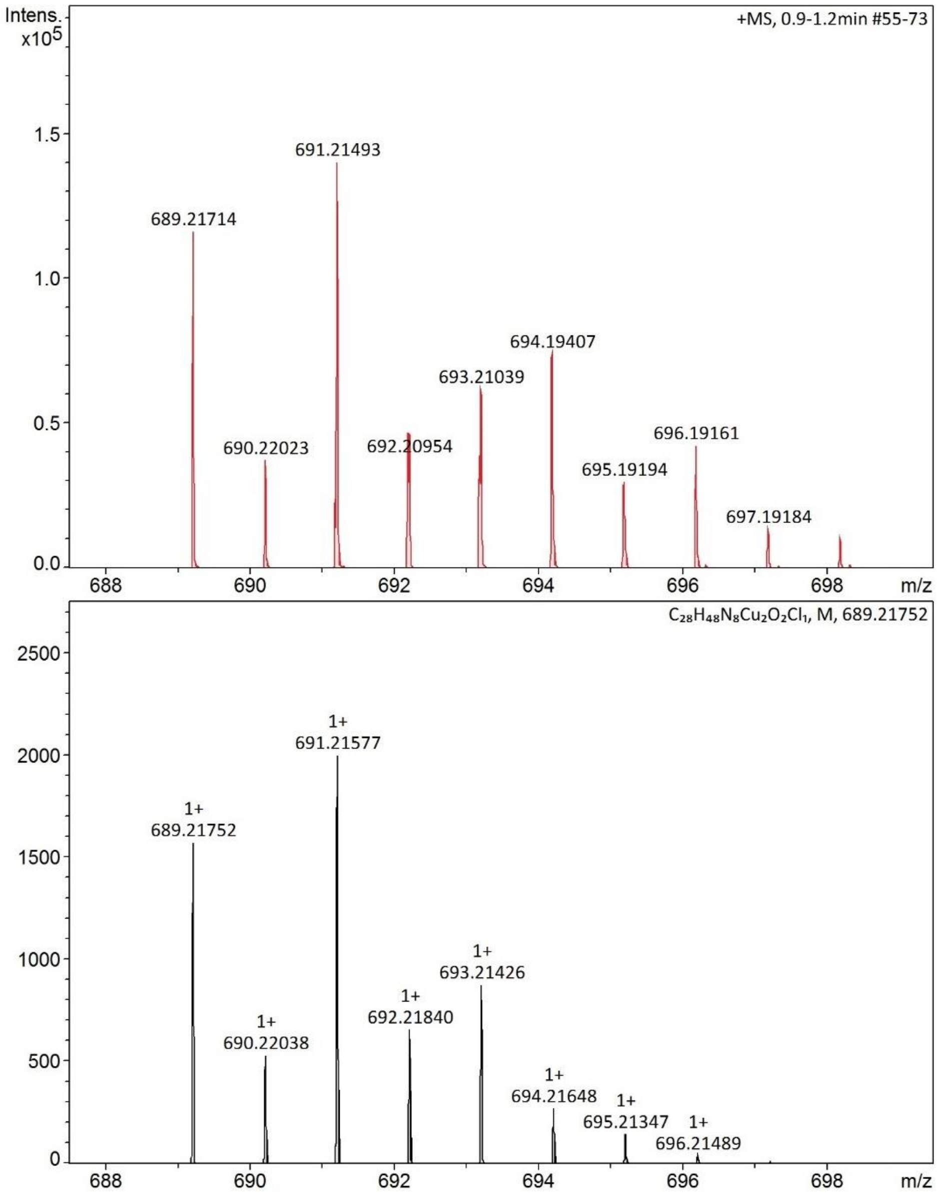

Figure S28: Cryo-UHR-ESI mass spectrometry of [**O1**](Cl)$^+$ in tetrahydrofuran at –100 °C (top: experimental, bottom: calculated). The isotopic pattern and corresponding m/z value exhibit the mass spectrum of the monocationic species [**O1**](Cl)$^+$, which was observed in the oxygenation of **C3a**.

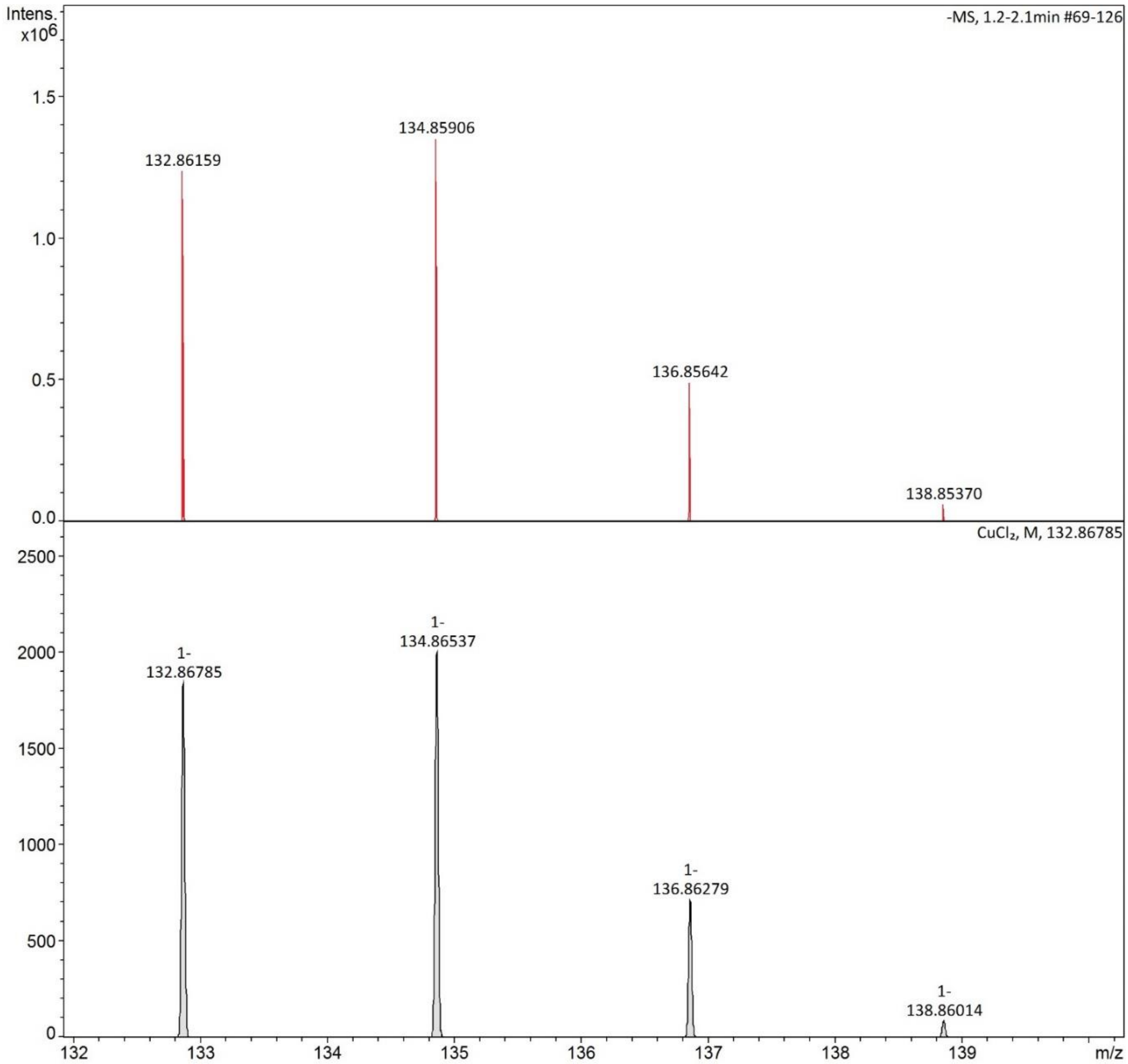

Figure S29: Cryo-UHR-ESI mass spectrometry in the negative mode of the oxygenation reaction of **C3a** in tetrahydrofuran at –100 °C (top: experimental, bottom: calculated). The isotopic pattern and corresponding m/z value exhibit the mass spectrum of the chloridocuprate $[CuCl_2]^-$.

3.5 Titration of [O1I]$^+$ with Ligand L1

Complex **C1a** was oxygenated to give [**O1I**]$^+$ (1.25 mM) according to the protocol described in the manuscript. Excess of O_2 was removed by three cycles of evacuation and purging with N_2. A fivefold stock solution of **L1** (15.5 mg, 2.5 µmol, 5.0 eq) in acetonitrile (0.5 mL) was prepared and one-fifth of it positioned in a Hamilton syringe. The titrant was added stepwise in 0.1 mL (1.0 eq) steps. The titration experiment was followed by UV/Vis spectroscopy (Figure S32). After stabilization of the optical spectrum, the next aliquot of **L1** was injected.

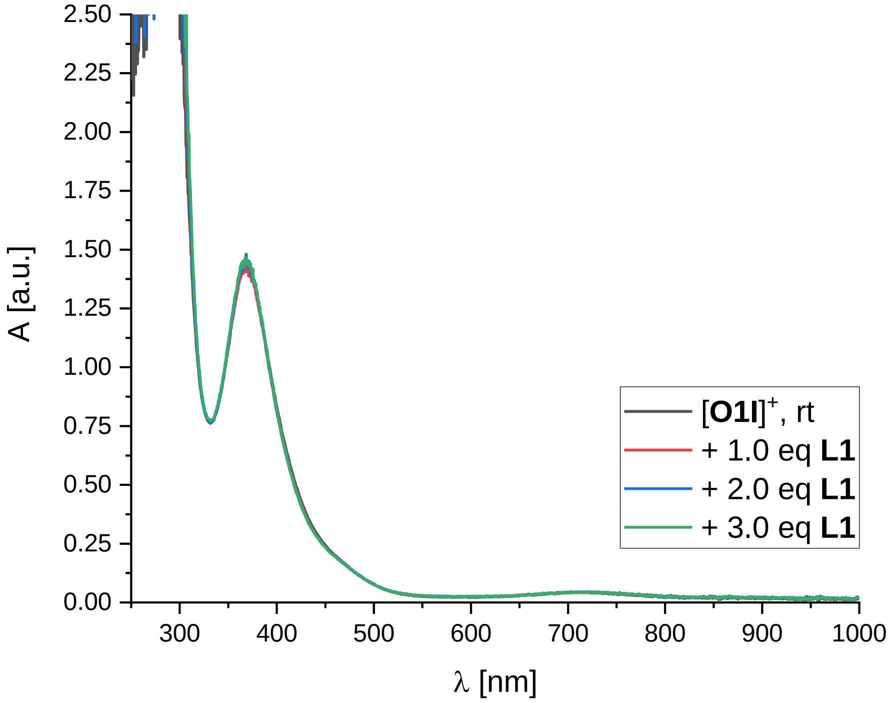

Figure 30: UV/Vis spectra of oxygenated **C1a** to give [**O1I**]$^+$ (1.25 mM) and the titration with **L1** in tetrahydrofuran at room temperature.

No color change was observed during titration. The absorption band at 370 nm remained constant upon addition of equivalents of hybrid guanidine ligand **L1**.

3.6 Titration of [O1I]$^+$ with Iodide Source Bu_4NI

Complex **C1a** was oxygenated to give [**O1I**]$^+$ (0.5 mM) according to the protocol described in the manuscript. Excess of O_2 was removed by three cycles of evacuation and purging with N_2. A tenfold stock solution of Bu_4NI (18.5 mg, 50.0 µmol, 10.0 eq) in acetonitrile (1.0 mL) was prepared and one-tenth of it positioned in a Hamilton syringe. The titrant was added stepwise in 0.1 mL (1.0 eq) steps. The titration experiment was followed by UV/Vis spectroscopy (Figure S31). After stabilization of the optical spectrum, the next aliquot of Bu_4NI was injected.

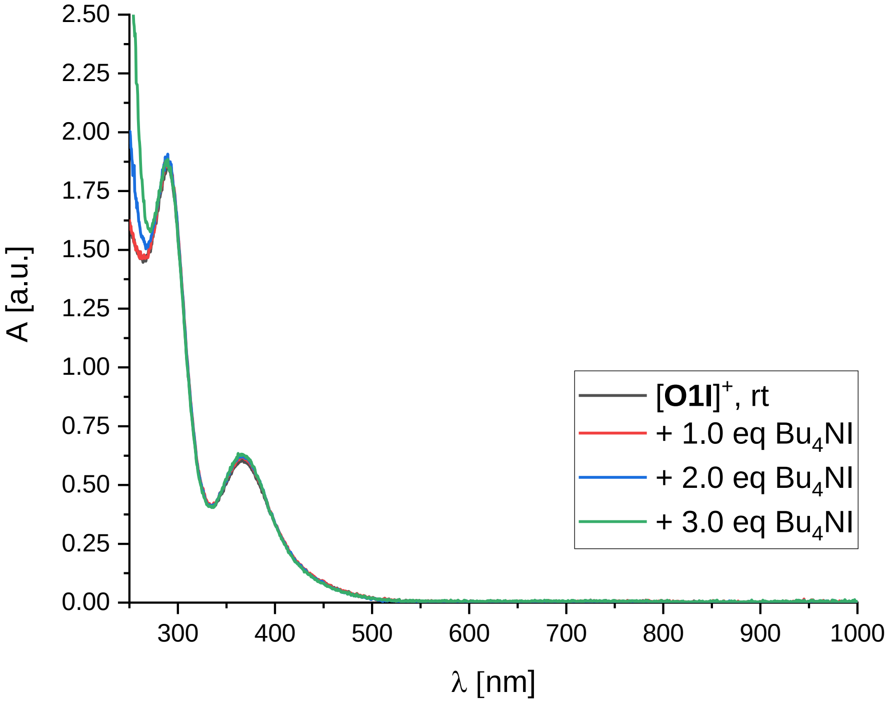

Figure S31: UV/Vis spectra of the titration of [**O1I**]$^+$ (0.5 mM) with Bu_4NI in tetrahydrofuran at room temperature.

No color change was observed. The absorption band at 370 nm remained constant upon addition of equivalents of tetrabutylammonium iodide.

3.7 Titration of [O1I]$^+$ with Copper Source [Cu(MeCN)$_4$]PF$_6$

Complex **C1a** was oxygenated at -80 °C to give [**O1I**]$^+$ (0.5 mM) according to the protocol described in the manuscript. Excess of O_2 was removed by three cycles of evacuation and purging with N_2. A tenfold stock solution of [Cu(MeCN)$_4$]PF$_6$ (18.6 mg, 50.0 µmol, 10.0 eq) in acetonitrile (1.0 mL) was prepared and one-tenth of it positioned in a Hamilton syringe. The titrant was added stepwise in 0.1 mL (1.0 eq) steps. The titration experiment was followed by UV/Vis spectroscopy (Figure S32). After stabilization of the optical spectrum, the next aliquot of [Cu(MeCN)$_4$]PF$_6$ was injected.

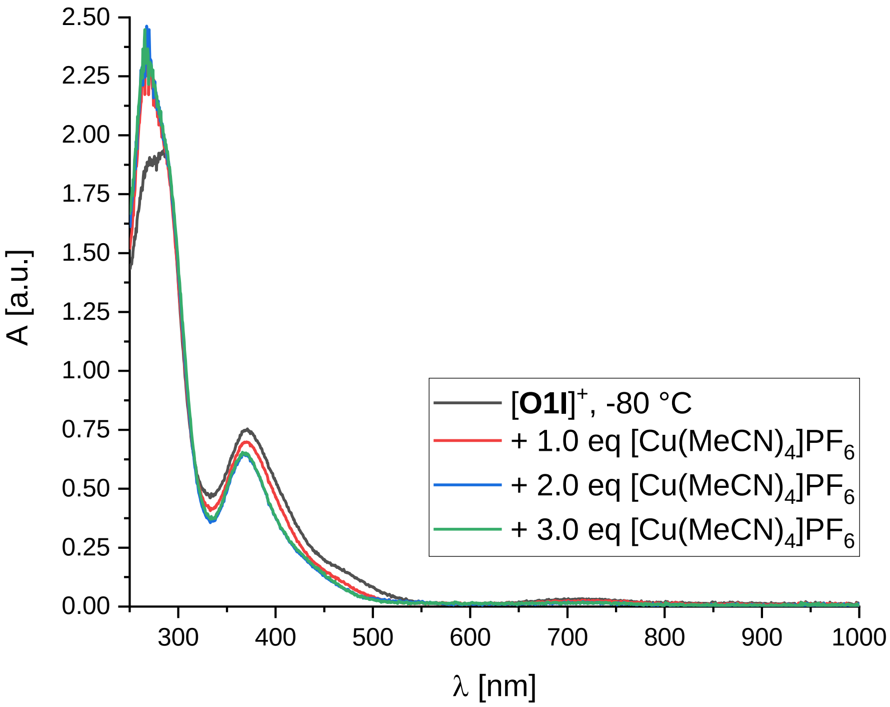

Figure S32: UV/Vis spectra of the titration of [**O1I**]$^+$ (0.5 mM) with [Cu(MeCN)$_4$]PF$_6$ in tetrahydrofuran at -80 °C.

The reddish-brown solution of [**O1I**]$^+$ discolored to yellowish-brown upon addition of the first aliquot of [Cu(MeCN)$_4$]PF$_6$ within 20 min. A slight decay of the absorption band at 370 nm was observed. Adding a second equivalent of [Cu(MeCN)$_4$]PF$_6$ resulted in a yellowish-green solution accompanied by a marginal blue-shift to 368 nm within 30 min. The absorption band remained constant upon addition of a third equivalent of [Cu(MeCN)$_4$]PF$_6$ over a period of 30 min.

3.8 Titration of [O1](PF$_6$)$_2$ with Bromide Source Bu$_4$NBr

The titration experiment was conducted according to the protocol described in the manuscript at -100 °C using tetrabutylammonium bromide and followed by UV/Vis spectroscopy (Figure S33).

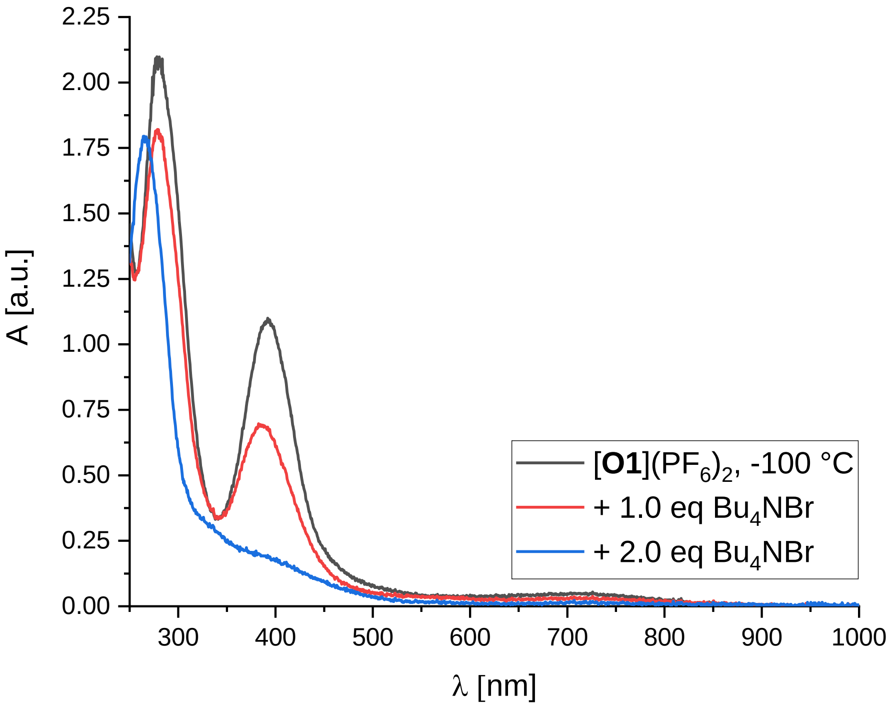

Figure S33: UV/Vis spectra of the salt metathesis of [**O1**](PF$_6$)$_2$ (0.5 mM) with Bu$_4$NBr in tetrahydrofuran at –100 °C.

Upon addition of 1.0 equivalent of Bu$_4$NBr the reaction solution discolored from khaki to brownish yellow. The absorption band at 390 nm shifted to 386 nm within minutes and remained constant after 10 min. Adding a second equivalent of Bu$_4$NBr to the reaction solution led to a renewed color change to yellow. The oxido band at 386 nm fully decayed after 10 min, showing the high reactivity of the oxido species, as observed for the direct oxygenation of **C2a**.

3.9 Titration of [O1](PF_6)$_2$ with Chloride Source Bu_4NCl

The titration experiment was conducted according to the protocol described in the manuscript using tetrabutylammonium chloride and followed by UV/Vis spectroscopy (Figure S34).

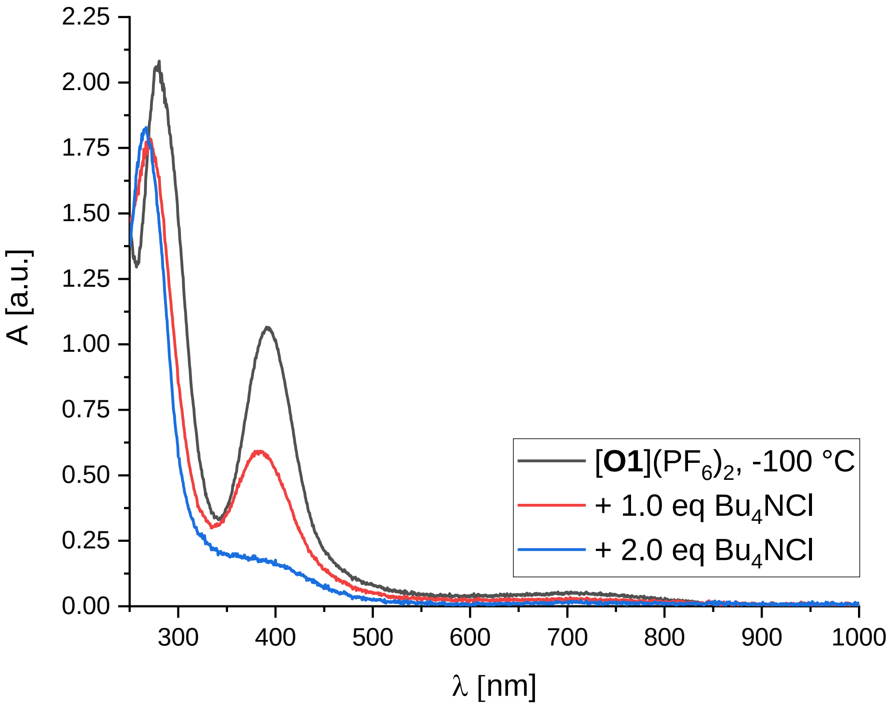

Figure S34: UV/Vis spectra of the salt metathesis of [**O1**](PF_6)$_2$ (0.5 mM) with Bu_4NCl in tetrahydrofuran at –100 °C.

Upon addition of 1.0 equivalent of Bu_4NCl the reaction solution discolored from khaki to brownish orange. The absorption band at 390 nm shifted to 384 nm within minutes and remained constant after 10 min. Adding a second equivalent of Bu_4NCl to the reaction solution led to a renewed color change to yellow. The oxido band at 384 nm fully decayed after 13 min, showing the high reactivity of the oxido species, as observed for the direct oxygenation of **C3a**.

3.10 Stability of [O1I](CuI_2) towards H_2O

Complex **C1a** was oxygenated to give [**O1I**](CuI_2) (0.5 mM) according to the protocol described in the manuscript. Excess of O_2 was removed by three cycles of evacuation and purging with N_2. Degassed H_2O (0.1 mL, 1 vol%) was added and the experiment was followed by UV/Vis spectroscopy (Figure S35). After stabilization of the optical spectrum, the next aliquot of degassed H_2O (1.0 mL, 10 vol%) was injected.

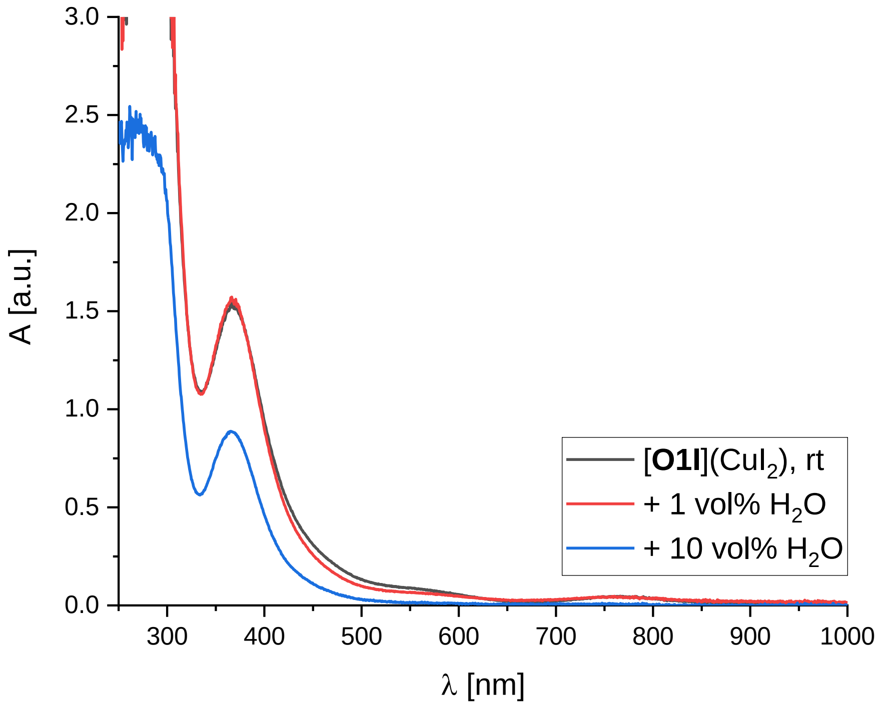

Figure S35: UV/Vis spectra of the stability of [**O1I**](CuI_2) (1.25 mM) in the presence of H_2O in tetrahydrofuran at room temperature.

Upon addition of 1 vol% of H_2O the absorption band at 370 nm remained constant and no color change was observed within 10 minutes. Addition of further amount of H_2O (10 vol%) led to a drop in intensity by half within 10 minutes and an immediate discoloration of the reaction solution to yellow was observed, showing a moderate stability of oxido species towards a small amount of water.

4 Catalytic Reactivity of [O1I](CuI_2)

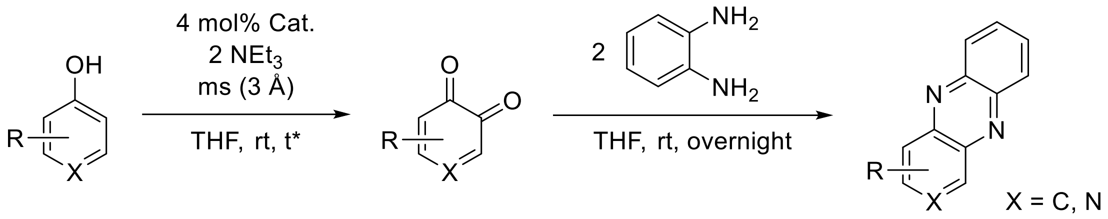

Scheme S1: Oxygenation of phenolic substrates mediated by [**O1I**](CuI_2) and subsequent reaction with 1,2-phenylenediamine.

The amount of solvent for the substrate solutions varied due to solubility limitations (Table S3).

Table S3: Substrate solutions used for oxygenation of phenolic substrates mediated by [**O1I**](CuI_2).

substrate	solvent	volume [mL]
2-naphthol	tetrahydrofuran	1.0
3-quinolinol	methanol	4.0
4-methoxy phenol	tetrahydrofuran	1.0
5-indolol	tetrahydrofuran	4.0
6-quinolinol	methanol	4.0
7-indolol	tetrahydrofuran	4.0

4.1 Reaction of [O1I](CuI_2) with Phenols

All reactions were conducted according to the protocol described in the manuscript.

Reaction of [**O1I**](CuI_2) with 4-methoxy phenol led to an immediate color change of the solution to reddish-brown. Cryo-UHR-ESI measurements of the reaction solution using 4-methoxy phenol revealed a m/z value of 139.0569 (calculated 139.0390) which was attributed to the quinone [M+H]+, 161.10730 (calculated 161.0209) for [M+Na]+, and a m/z value of 287.16339 (calculated 287.08899) which was attributed to the o-coupled catechol [M+Na]+.

5 Control Experiments

5.1 Reaction of [O1I](CuI_2) with Triethylamine

Complex **C1a** was oxygenated to give [**O1I**](CuI_2) (0.5 mM) according to the protocol described in the manuscript. After addition of triethylamine (0.07 mL) no immediate color change was observed. The reaction was followed by UV/Vis spectroscopy. The decrease of the absorption band at 370 nm was observed over time (Figure S36). After 80 min the reaction solution discolored slightly to orange.

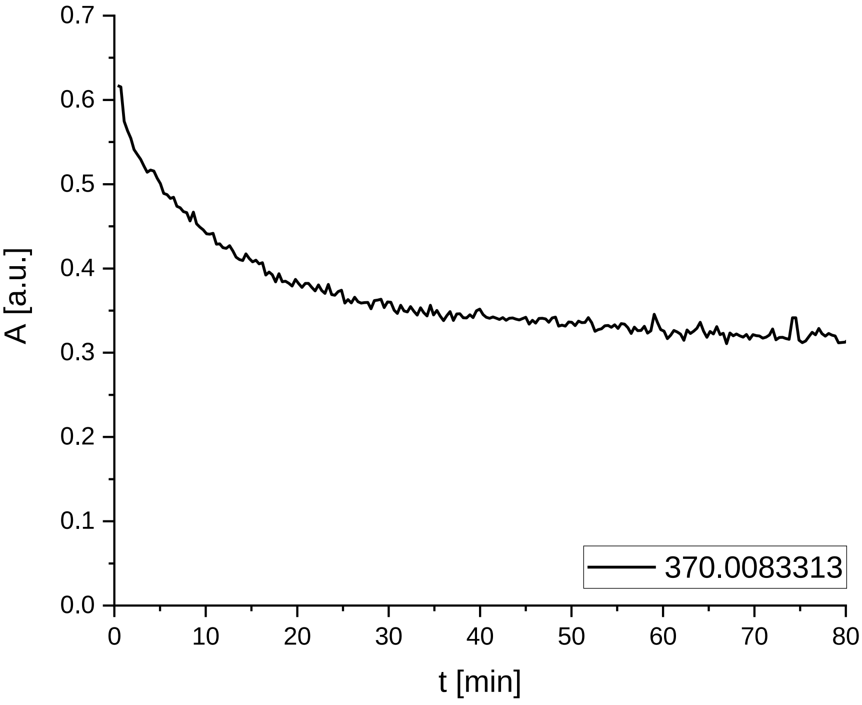

Figure S36: UV/Vis spectrum of the reaction of [**O1I**](CuI_2) (0.5 mM) with triethylamine in tetrahydrofuran at room temperature monitored at 370 nm.

5.2 Reaction of [O1I](CuI_2) with 1,2-Phenylenediamine

Complex **C1a** was oxygenated to give [**O1I**](CuI_2) (0.5 mM) according to the protocol described in the manuscript. Reaction of [**O1I**]$^+$ with 1,2-phenylenediamine led to an immediate color change to violet. The reaction was followed by UV/Vis spectroscopy (Figure S37). At room temperature, the decrease of the absorption band at 370 nm and simultaneously the formation of absorption bands at 550 nm and 950 nm were observed, similar to those observed previously [1].

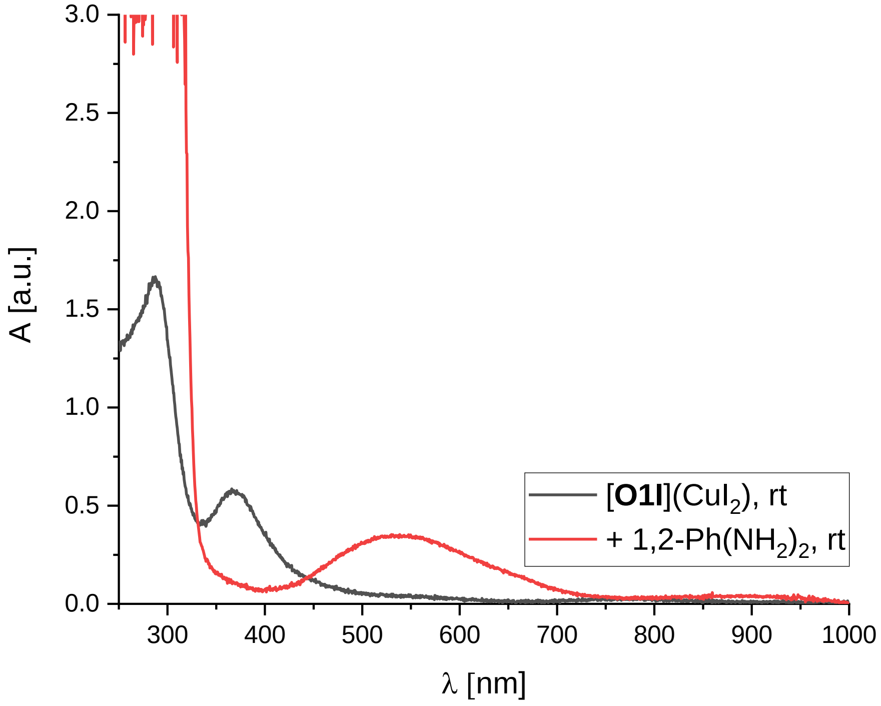

Figure S37: UV/Vis spectra of the reaction of 1,2-phenylenediamine with [**O1I**](CuI_2) (0.5 mM) in tetrahydrofuran at room temperature.

5.3 Reaction of Copper Iodide with 2-Naphthol in the Presence of O_2 and Subsequent Reaction with 1,2-Phenylenediamine

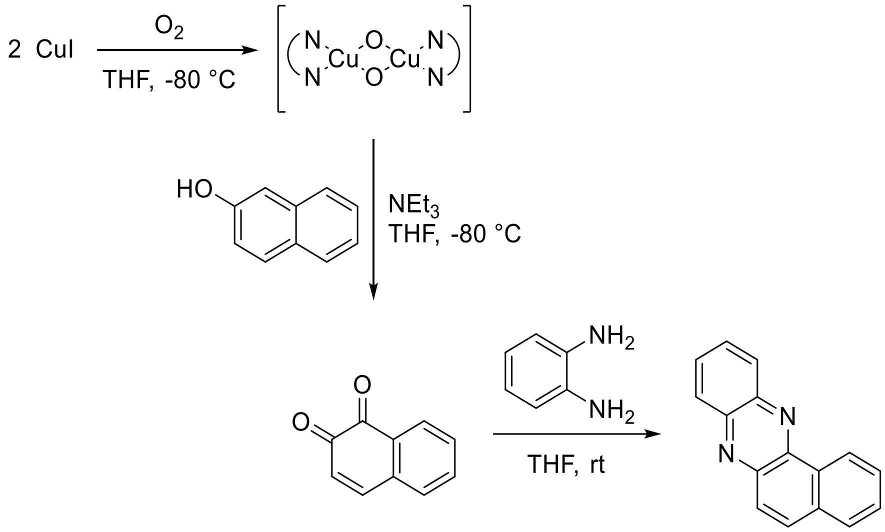

Scheme S2: Reaction of copper iodide (1.0 mM) with 2-naphthol in the presence of O_2 in tetrahydrofuran at -80 °C and subsequent reaction with 1,2-phenylenediamine.

The control experiment was conducted according to the protocol described in the manuscript without the ligand to proof the necessity of a stabilizing ligand system for the catalyst. Reaction of copper iodide with molecular oxygen only revealed a small visible absorbance at 270 nm in the UV/Vis spectrum, indicating no formation of a bis(μ-oxido) dicopper(III) species (Figure S38). Addition of a 2-naphthol solution led to the formation of an absorption band at 330 nm within two hours, which was referred to the substrate itself. Subsequent reaction with 1,2-phenylenediamine resulted in the formation of an absorption band at 570 nm at -80 °C. Upon warming to room temperature, absorption bands at 360, 380, 402 and 555 nm were observed. After an acidic workup, the ^1H NMR spectrum revealed a substrate-to-product ratio of 97:3 (2-naphthol-to-benzo[a]phenazine ratio).

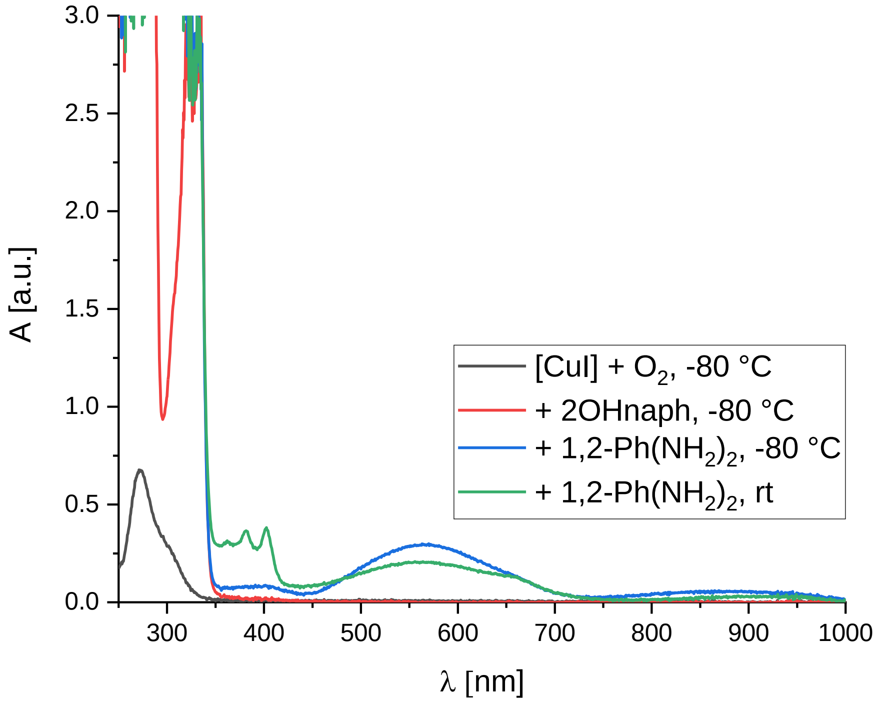

Figure S38: UV/Vis spectra of the reaction of copper iodide (1.0 mM) with 2-naphthol in the presence of O_2 in tetrahydrofuran at -80 °C and subsequent reaction with 1,2-phenylenediamine.

6 TD-DFT Calculations on $[O1]^{2+}$ and $[O1I]^{+}$

LUMO+3

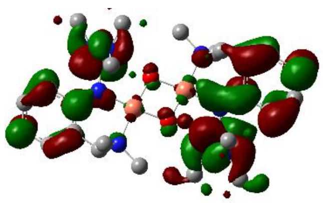

LUMO+2

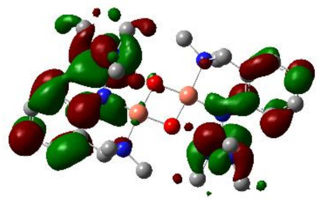

LUMO+1

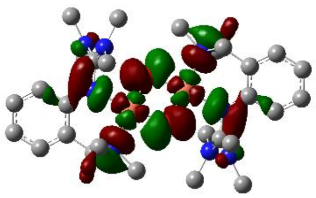

LUMO

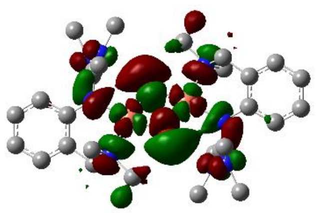

HOMO

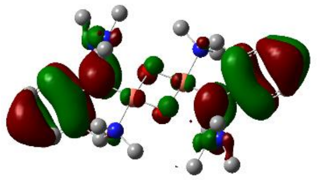

HOMO-1

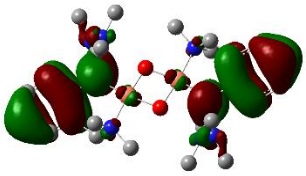

HOMO-2

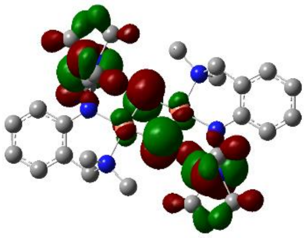

HOMO-3

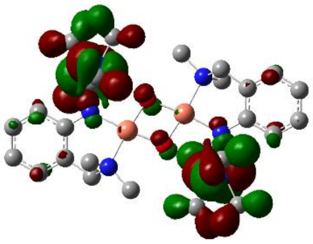

HOMO-4

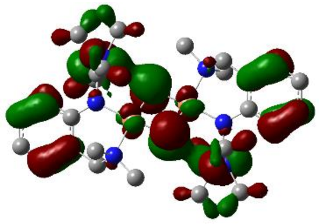

HOMO-5

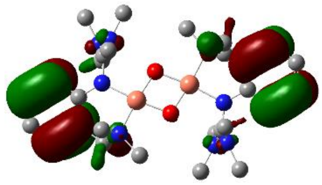

HOMO-6

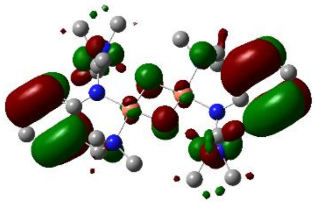

HOMO-7

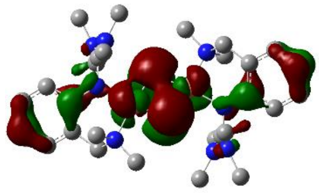

HOMO-8

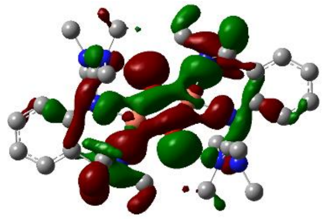

HOMO-9

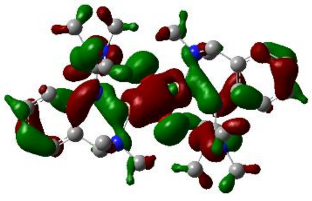

HOMO-10

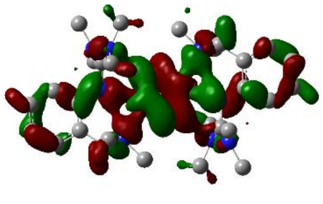

HOMO-11

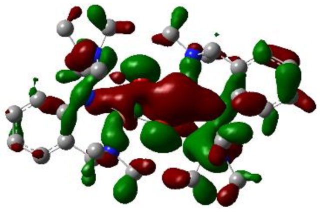

HOMO-12

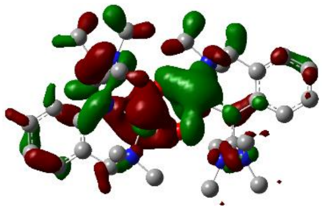

HOMO-13

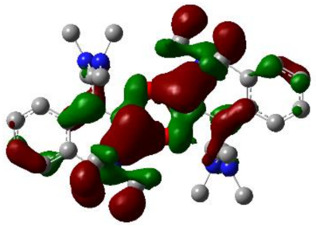

HOMO-14

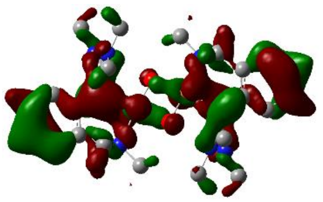

HOMO-15

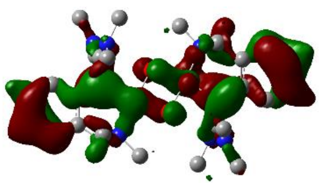

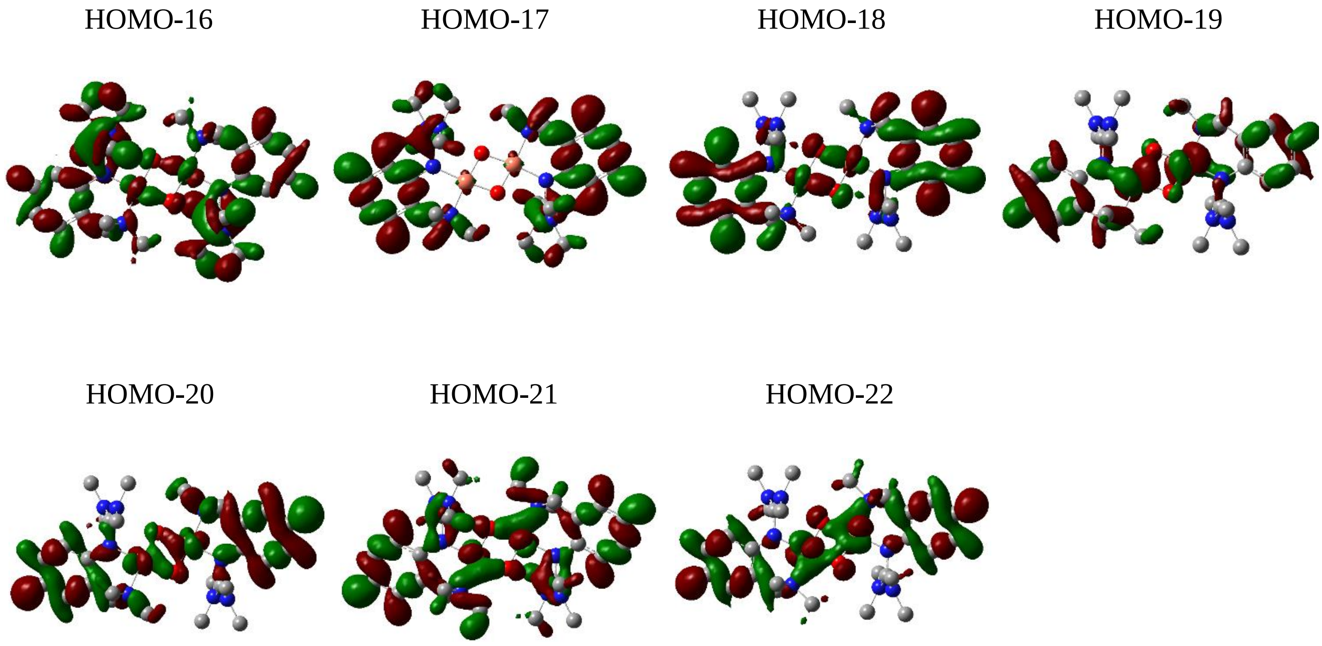

Figure S39: Selected molecular orbitals of [**O1**]$^{2+}$.

Table S4: MO energies of [**O1**]$^{2+}$.

MO	E [H]	e [eV]	MO composition
LUMO+3	-0.06817	-1.85500114	π*(benza) and π*(gua)
LUMO+2	-0.06866	-1.86833472	π*(benza) and π*(gua)
LUMO+1	-0.16427	-4.47001668	π_σ* - Cu d - N
LUMO	-0.17384	-4.73042978	σ* - Cu d - N
HOMO	-0.23284	-6.33590238	π*(benza) - π(gua) - π_v (nb)
HOMO-1	-0.23558	-6.41046161	π*(benza) and π*(gua)
HOMO-2	-0.26339	-7.16721065	Cu d - π_v* and LP N(amine)
HOMO-3	-0.26584	-7.23387858	LP N(amine) and π_v (nb)
HOMO-4	-0.27086	-7.3704798	Cu d - π_v* and π*(benza) and LP N(amine)
HOMO-5	-0.27311	-7.43170545	LP(benza)
HOMO-6	-0.27349	-7.44204579	LP(benza) and π_v*
HOMO-7	-0.2915	-7.9321231	Cu d - π_v
HOMO-8	-0.29791	-8.10654817	Cu d + σ* - N
HOMO-9	-0.30912	-8.41158797	Cu d + σ + N
HOMO-10	-0.31692	-8.62383689	Cu d + π_σ + N
HOMO-11	-0.32454	-8.83118776	Cu d - σ*(tilted) + N(gua)
HOMO-12	-0.32508	-8.84588191	Cu d (tilted) + π_σ + N
HOMO-13	-0.32832	-8.93404685	N(amine) + Cu d + π_σ*(tilted)
HOMO-14	-0.33167	-9.02520504	Cu d + N(gua) and benza and π_v*
HOMO-15	-0.3328	-9.05595392	Cu d + N(gua) and π(benza) and π_v*
HOMO-16	-0.35684	-9.71011598	σ(benza) and π(gua) and d - π_σ*(tilted)
HOMO-17	-0.35664	-9.7046737	σ(benza) and σ(gua)
HOMO-18	-0.36021	-9.80181839	σ(benza) and Cu d and tilted π_σ
HOMO-19	-0.36713	-9.99012128	σ(benza) and Cu d
HOMO-20	-0.36875	-10.0342038	σ(benza) and Cu d + π_σ
HOMO-21	-0.36933	-10.0499864	σ(benza) and Cu d - π_σ*
HOMO-22	-0.37183	-10.1180149	σ(benza) and Cu d and σ

LUMO+3 LUMO+2 LUMO+1 LUMO

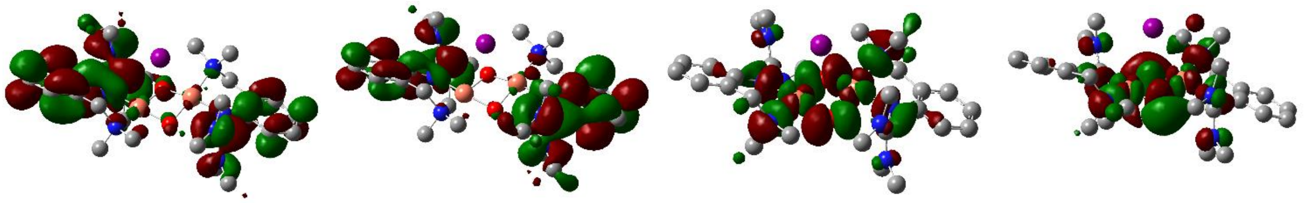

HOMO HOMO-1 HOMO-2 HOMO-3

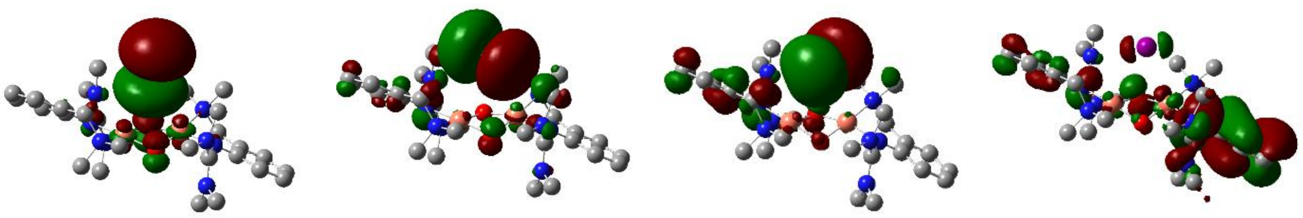

HOMO-4 HOMO-5 HOMO-6 HOMO-7

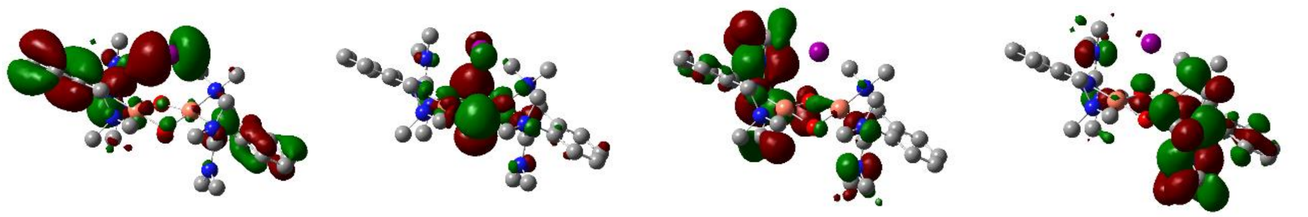

HOMO-8 HOMO-9 HOMO-10 HOMO-11

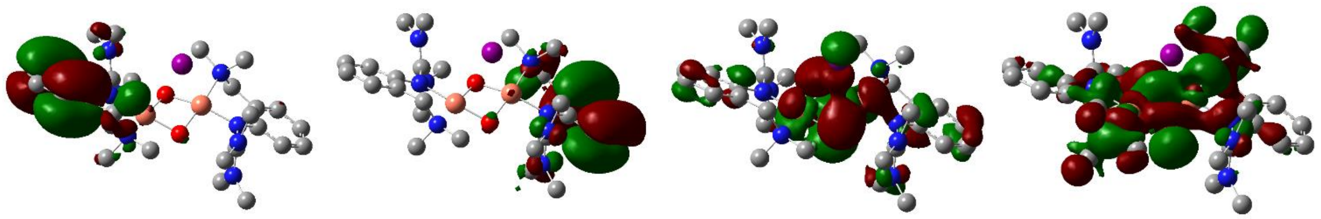

HOMO-12 HOMO-13 HOMO-14 HOMO-15

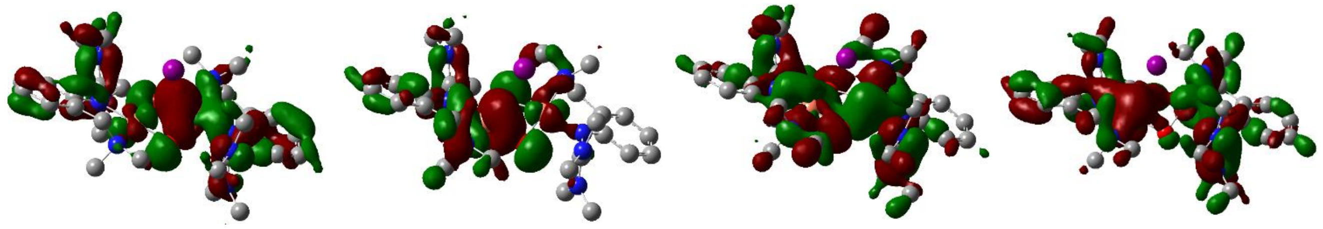

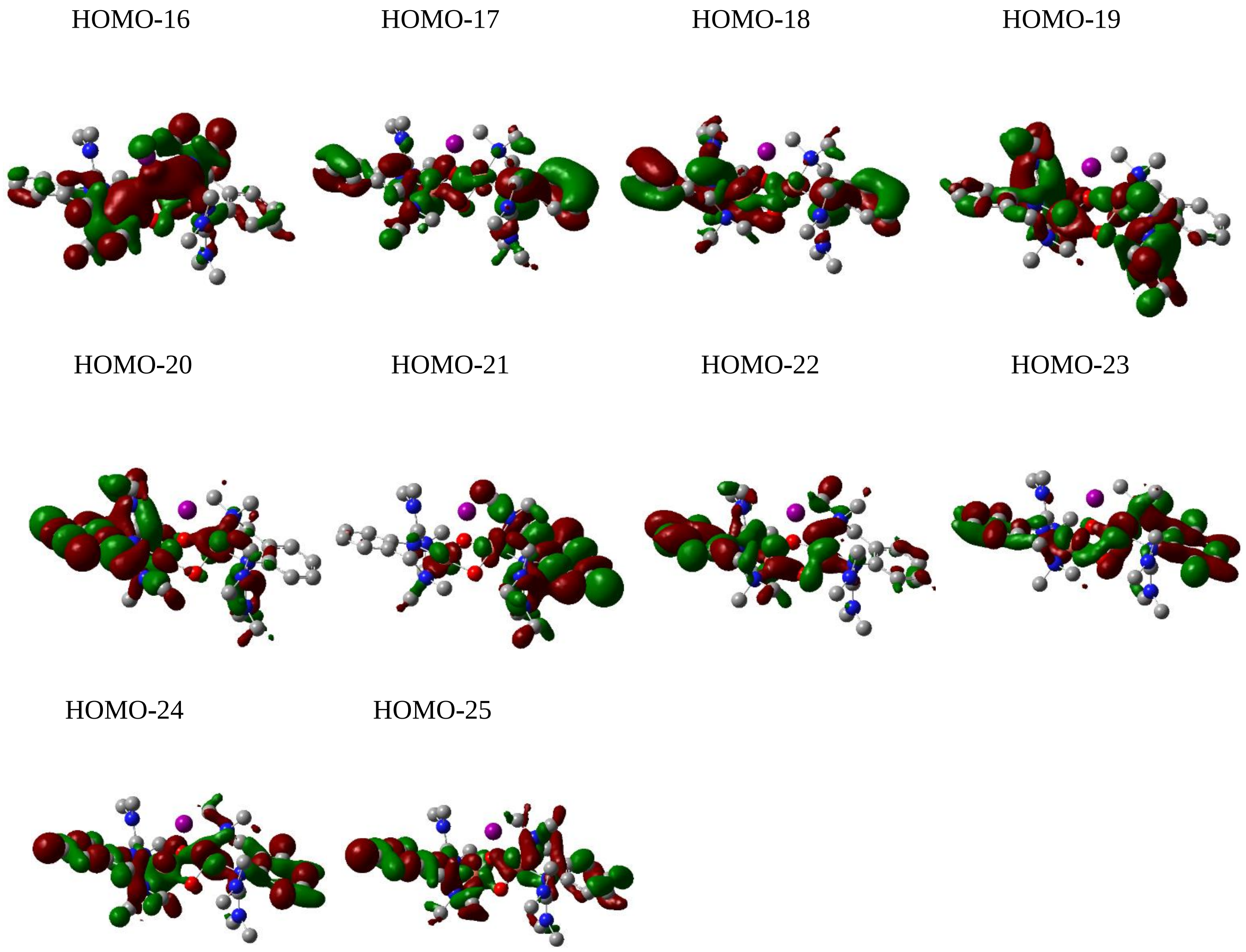

Figure S40: Selected Molecular orbitals of [**O1I**]$^{+}$.

Table S5: MO energies of [**O1I**]$^{+}$.

MO	E [H]	e [eV]	MO composition
LUMO+3	-0.0525	-1.4285985	π*(benza) and π*(gua)
LUMO+2	-0.05323	-1.44846282	π*(benza) and π*(gua)
LUMO+1	-0.14543	-3.9573539	π_σ* - Cu d - N
LUMO	-0.14997	-4.08089366	σ* - Cu d - N
HOMO	-0.20653	-5.61997044	LP(I)
HOMO-1	-0.20996	-5.71330554	LP(I)
HOMO-2	-0.2108	-5.73616312	LP(I)
HOMO-3	-0.2171	-5.90759494	π*(benza) and π(gua)
HOMO-4	-0.22128	-6.02133859	π*(benza) and LP(I)
HOMO-5	-0.24055	-6.54570227	Cu d - π_v*
HOMO-6	-0.24961	-6.79223755	π*(gua)
HOMO-7	-0.25232	-6.86598045	π*(gua)
HOMO-8	-0.25928	-7.05537179	π*(benza)
HOMO-9	-0.26017	-7.07958994	π*(benza)
HOMO-10	-0.26822	-7.29864171	Cu d - π_v + LP(I)
HOMO-11	-0.27974	-7.61211704	Cu d + σ* - N
HOMO-12	-0.28927	-7.87144168	Cu d - π_σ(tilted) + N
HOMO-13	-0.29086	-7.9147078	linear combination of π_σ(tilted) and σ + N
HOMO-14	-0.30291	-8.24260517	Cu d - σ(tilted) + N
HOMO-15	-0.30487	-8.29593952	linear combination π_v and π_σ bonding with Cu d and + N
HOMO-16	-0.30895	-8.40696203	π_σ*(tilted) + N + LP(I) + d
HOMO-17	-0.31488	-8.56832563	N(gua) + Cu d - π_v(tilted) and π(benza)
HOMO-18	-0.31624	-8.60533314	Cu d - π_v(tilted) + N(gua) and π(benza)
HOMO-19	-0.33517	-9.12044494	Cu d - π_v(tilted) + N and π(gua)
HOMO-20	-0.34031	-9.26031153	σ(benza) and Cu d
HOMO-21	-0.3428	-9.32806792	σ(benza) and σ(gua) and Cu d
HOMO-22	-0.34647	-9.42793376	σ(benza) and σ(gua) and Cu d
HOMO-23	-0.34705	-9.44371637	σ(benza) and Cu d
HOMO-24	-0.35065	-9.54167741	σ(benza) and Cu d
HOMO-25	-0.35158	-9.56698401	σ(benza) and Cu d and N

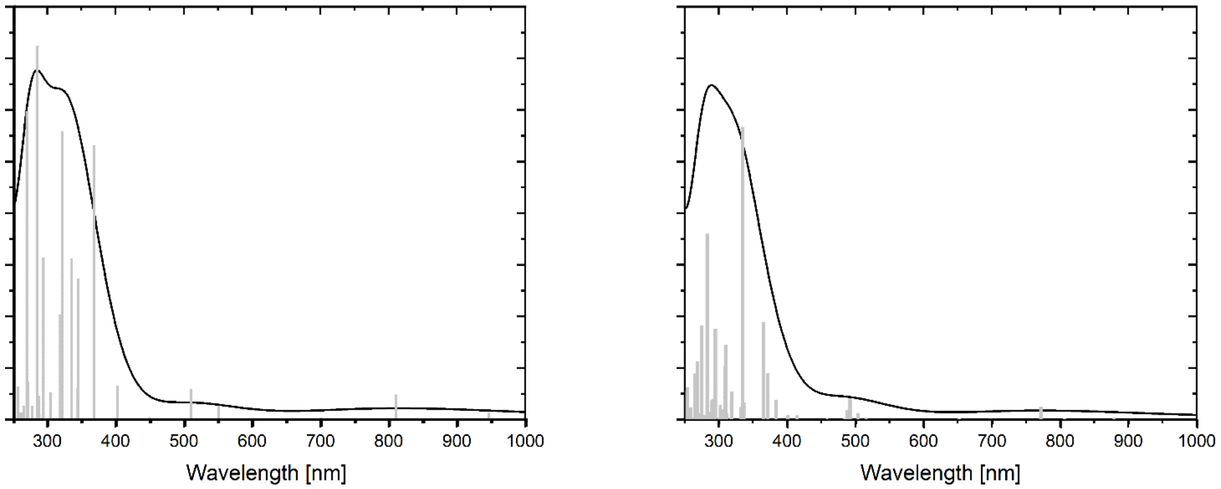

Figure S41: TD-DFT-predicted optical spectra of [**O1**]$^{2+}$ (left) and [**O1I**]$^{+}$ (right) with TPSSh/def2-TZVP with solvent THF and GD3BJ.

7 References

1. Paul M, Teubner M, Grimm-Lebsanft B, Golchert C, Meiners Y, Senft L, Keisers K, Liebhäuser P, Rösener T, Biebl F, Buchenau S, Naumova M, Murzin V, Krug R, Hoffmann A, Pietruszka J, Ivanović-Burmazović I, Rübhausen M, Herres-Pawlis S (2020) Chem Eur J. 26:7556-7562.
